# Tissue flow acts as a guidance cue for immune cell polarization and directional migration

**DOI:** 10.1038/s41556-026-02058-9

**Published:** 2026-09-07

**Authors:** Hoang Anh Le, Jonas Hartmann, Ricard Alert, Roberto Mayor

**Affiliations:** 1 Department of Cell and Developmental Biology, University College London, London, UK; 2 Max Planck Institute for the Physics of Complex Systems, Dresden, Germany; 3 Center for Systems Biology Dresden, Dresden, Germany; 4 Cluster of Excellence Physics of Life, Dresden, Germany; 5 Departament de Física de la Matèria Condensada, Universitat de Barcelona, Barcelona, Spain; 6 University of Barcelona Institute of Complex Systems (UBICS), Barcelona, Spain; 7 Institució Catalana de Recerca i Estudis Avançats (ICREA), Barcelona, Spain; 8 Center for Integrative Biology, Faculty of Sciences, Universidad Mayor, Santiago, Chile

**Keywords:** Collective cell migration, Embryology, Amoeboid migration, Monocytes and macrophages

## Abstract

Embryonic myeloid (EM) cells are the first immune-cell population to emerge during development and must disperse throughout the embryo to act as the first line of defence against infection. Although EM cells migrate directionally toward wounds, how they navigate through unwounded tissues during early colonization remains unclear. Here we show that EM cell dispersion in *Xenopus* embryos is driven, at least in part, by cell-on-cell migration, an important yet underappreciated phenomenon, guided by dynamic tissue flows. We have established a new ex vivo EM cell migration system that allows for live imaging, computational analyses and optogenetic manipulation. We find that local ectodermal tissue flows repolarize EM cell protrusions and bias their directional migration. Disrupting these flows, both ex vivo and in vivo, either genetically or mechanically, impairs EM cell dispersion. Our findings reveal that mechanical cues generated by surrounding tissue flows coordinate immune-cell migration during development, highlighting an overlooked mechanism by which collective tissue dynamics guide individual cell behaviour.

## Abstract (web summary)

Le et al. show that in *Xenopus* embryos, local ectodermal tissue flows provide a directional cue that polarizes and guides embryonic myeloid cells migration via cell-on-cell interactions.

## Main

When studying cell migration, we often disproportionally focus on cell–matrix interactions^1–7^. However, within the crowded tissue environment in vivo, migrating cells also encounter other cells. Historically, studies on cell–cell interactions have been more successful in vivo. In *Drosophila melanogaster*, border cells form E-cadherin adhesions with surrounding nurse cells to migrate^8,9^. In zebrafish embryos, the posterior lateral line primordium (pLLP) cells form focal adhesions to the basal surface of the skin as it moves^10–13^. Studies in this regard have laid the foundation for an important but often overlooked concept, whereby cells can serve as a living substrate for other cells to interact with and migrate along^13–16^, a phenomenon called cell-on-cell migration.

In vertebrates, embryonic myeloid (EM) cells are among the first to populate early embryos. They clear apoptotic corpses^17,18^, respond to bacterial infections^17^, are essential to organ development^19–21^, and form various tissue-resident macrophage lineages^22,23^. In *Xenopus laevis* embryos, EM cells originate from the anterior ventral blood island (aVBI) near the head (the equivalent of the anterior lateral plate mesoderm (ALPM) in zebrafish and the yolk sac in mammals^24–26^). EM cells initiate their dispersion around stage 20, just after the closure of the neural folds, and populate the entire embryo by stage 27—one of the final stages in the early tailbud phase. Unlike other migratory embryonic cell populations, such as the neural crest, which migrate collectively^27–29^, EM cells disperse as single cells^25^. They move along the anterior–posterior and ventral–dorsal axes. Traditionally, the mechanisms governing single-cell migration have often been explored in the context of cancer, where cells use a chemical gradient (such as lysophosphatidic acid^30,31^) or a stiffness gradient^32^ as guidance cues. However, the mechanisms that guide single immune-cell dispersion, such as within a developing embryo, remain largely unexplored.

**Fig. 1:**
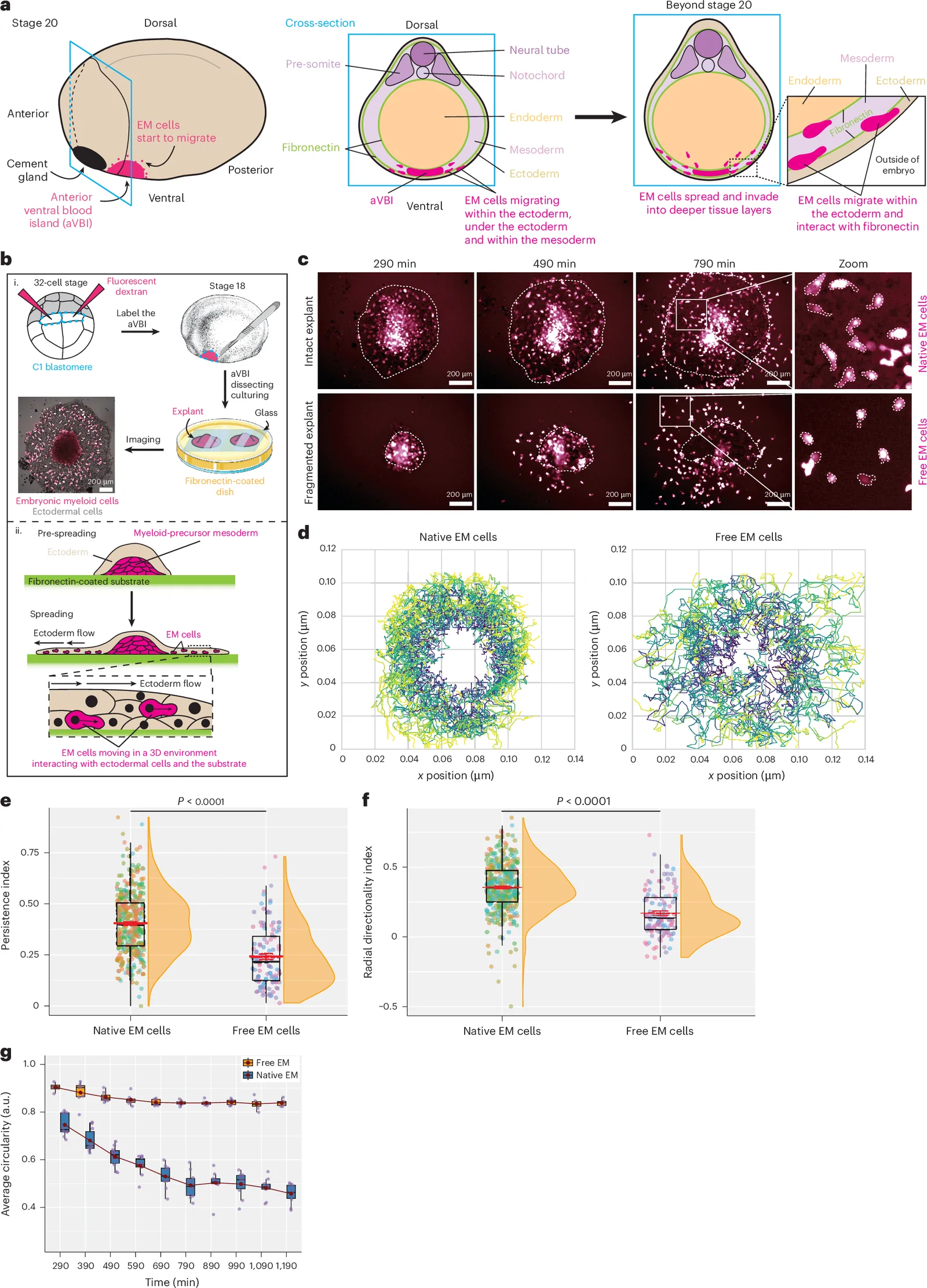
The ectoderm enables EM cells’ polarity and radial migration. **a**, Schematic diagram showing the anatomy of a stage-20 *X. laevis* embryo and the location of the aVBI. A cross-section (cyan) through the anterior part of the embryo showing the different tissue layers. Single EM cells disperse from the aVBI and migrate within the ectoderm, under the ectoderm, and between mesodermal cells, and later invade into the deeper endoderm layer. A fibronectin layer (green) sits between the ectoderm and the mesoderm, and between the mesoderm and the endoderm. A zoom-in of the cross-section shows the anatomical organization of EM cells (pink) in relation to the three germ layers and the fibronectin matrix. Migrating EM cells interact with surrounding ectodermal cells and with the fibronectin matrix layer. **b**, (i) Schematic workflow for the ex vivo culturing of the aVBI from *X. laevis* embryos. (ii) Schematic depiction of the organization of an aVBI explant spreading on a fibronectin-coated substrate. The myeloid-precursor mesoderm (pink) sits at the centre of the explant and gives rise to EM cells, and the ectoderm layer sits on top. As the ectoderm layer spreads, EM cells move through the ectoderm in a 2.5D–3D environment and interact with both the surrounding ectodermal cells and the fibronectin substrate (green) underneath, as seen in vivo. **c**, Comparing the morphology of EM cells in a native explant with an ectodermal layer (native EM cells) versus EM cells outside the explant (free EM cells). Cells were labelled with fluorescent dextran (magenta). Explant outlines and cell outlines are shown (dotted white). Scale bars, 200 μm. See Supplementary Video 3 for further details. **d**, Migration trajectory plots of native EM cells versus free EM cells. A lighter colour means further away from the starting point. **e**, Box plots comparing the average migration persistence index (a.k.a. persistence) between native EM cells (mean = 0.405, *n* = 379 cells, *N* = 10 embryos) and free EM cells (mean = 0.243, *n* = 114 cells, *N* = 7 embryos). Two-tailed unpaired *t*-test. Mean ± s.e.m. **f**, Box plots comparing the average radial directionality index between native EM cells (mean = 0.355, *n* = 379 cells, *N* = 10 embryos) and free EM cells (mean = 0.17, *n* = 114 cells, *N* = 7 embryos). Two-tailed unpaired *t*-test. Mean ± s.e.m. **g**, Comparison of the average circularity index between native EM cells (*N* = 10 embryos) and free EM cells (*N* = 7 embryos). The trend line (red) connects mean values. Box plots show the interquartile range (IQR) with minimum = Q1 − 1.5 × IQR and maximum = Q3 + 1.5 × IQR and a half violin plot showing the data distribution.

Early *Xenopus* embryos lack a functional circulatory system, similar to zebrafish^17^, so dispersing EM cells can migrate in between and underneath ectodermal cells, in between mesodermal cells, and later invade into the deeper endodermal tissue^24–26^ (Fig. 1a). Cross-sectional images of later-stage embryos show confined bundles of fibronectin separating the ectoderm from the mesoderm, and the mesoderm from the endoderm^33–35^, rather than interdigitating between these cells, suggesting that EM-cell dispersion through tissue is primarily a cell-on-cell migration process. To dissect the mechanism of how EM cells navigate through dense tissue, we have developed an ex vivo culturing system from *Xenopus* embryos that preserves some aspects of the natural tissue anatomy and the developmental dynamics of the EM cells while also being highly reproducible and easy to set up. Using live imaging, we show that the ectoderm is necessary to maintain the normal polarized morphology and directional migration of EM cells. Correlation analyses, mathematical modelling and optogenetic experiments reveal that EM-cell protrusion dynamics and directionality are physically biased by the local flow of neighbouring ectodermal cells. Disrupting this flow ex vivo or in vivo substantially disrupts EM-cell dispersion. Overall, our results demonstrate that tissue flow can act as a mechanical cue to guide the migration of embedded single migrating immune cells.

## Results

### The ectoderm enables EM cell polarity and radial migration

**Extended Data Fig. 1:**
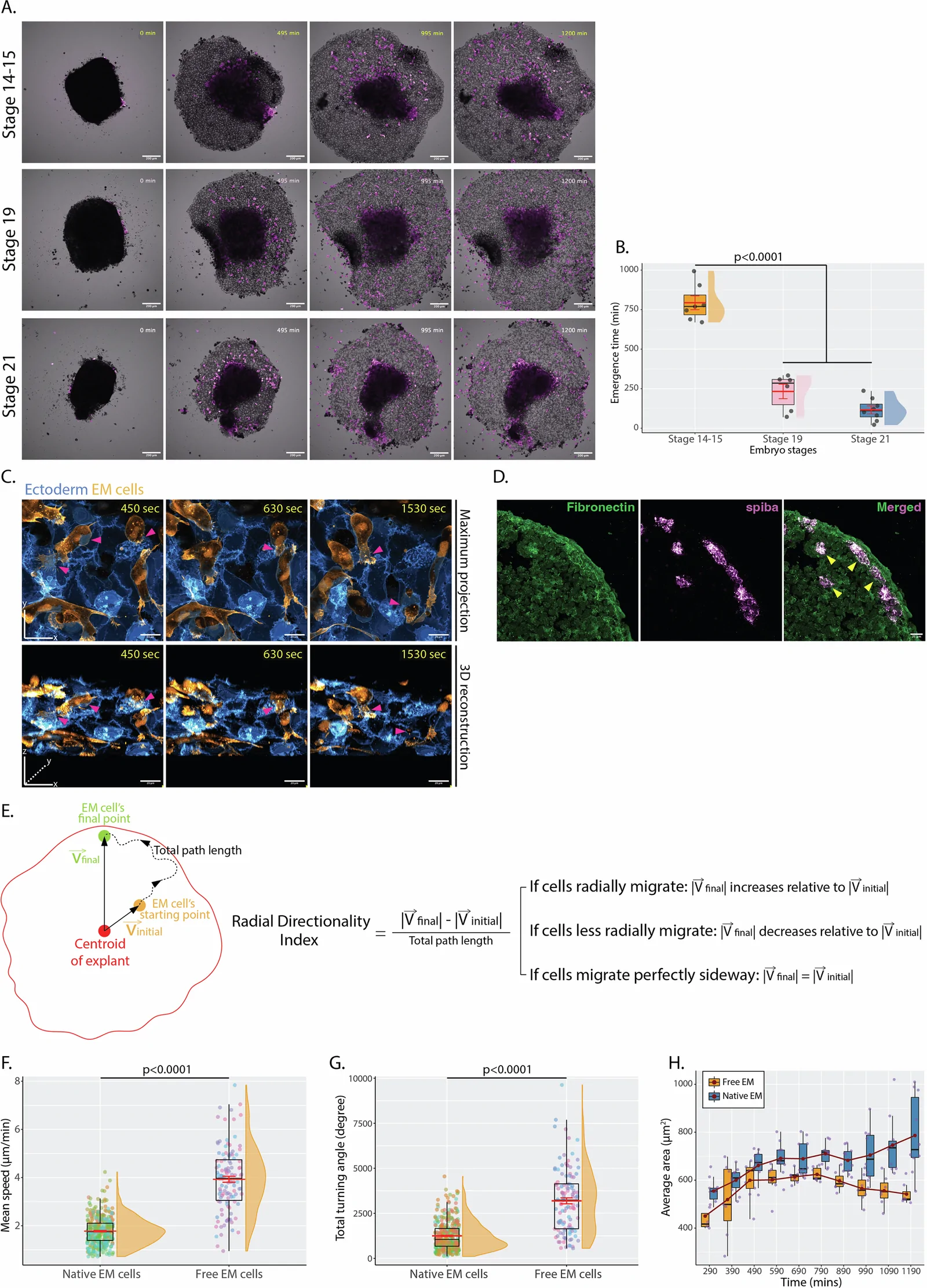
EM cells preserve their normal development within the explant culture. **A**. Representative image montage of aVBI explants with labelled EM cells (magenta) dissected from embryonic stage 14–15, 19, or 21. EM cells retained their normal development within the dissected explants and emerged from the mesoderm tissue later in early-stage explants compared to later-stage ones. Scale bar = 200 μm. Supplementary Video 1. **B**. Box plots comparing the average emergence time from the mesoderm of EM cells in aVBI explants dissected from stage 14-15 embryos (Mean = 794 min, N = 7 explants), stage 19 embryos (Mean = 232 min, N = 6 explants), and stage 21 embryos (Mean = 116 min, N = 8 explants). ANOVA with Tukey’s multiple comparison test. Mean ± SEM. **C**. Representative maximally projected and tilted 3D reconstructed images showing native EM cells (Ruby-LifeAct, orange) within an intact ectoderm layer (RFP-membrane, blue) *ex vivo*. EM cells are free to migrate in all 3 dimensions and make extensive contacts with surrounding ectodermal cells. Arrows point to the area of EM cells above or below ectodermal cells. Scale bar = 20 μm. Supplementary Video 2. **D**. Representative Immunostaining-FISH images of a cryosection of a stage 25 embryo showing the natural distribution of EM cells *in vivo*. EM cells (arrows) migrate underneath the ectoderm and invade into the deeper tissue layers in later stages. EM cells were visualised with *spiba* FISH (magenta), fibronectin in green. Scale bar = 25 μm. Reproducibility was confirmed from at least 12 different embryos mixed from 3 different female frogs in 2 different days. **E**. Schematic diagram explaining the strategy to calculate Radial Directionality Index. Detailed explanation in the method section. **F**. Box plots comparing the average speed between native EM cells (Mean = 1.776 μm/min, n = 339 cells, N = 10 embryos) and free EM cells (Mean = 3.927 μm/min, n = 111 cells, N = 7 embryos). Two-tailed unpaired t-test. Mean ± SEM. **G**. Box plots comparing the average total turning angle between native EM cells (Mean = 1252.37, n = 379 cells, N = 10 embryos) and free EM cells (Mean = 3194.43, n = 114 cells, N = 7 embryos). Two-tailed unpaired t-test. Mean ± SEM. **H**. Box plots comparing the average cell area between native EM cells (N = 10 embryos) and free EM cells (N = 7 embryos) over time. Two-tailed unpaired t-test. Mean ± SEM. Mean values at each time point are connected by a red line. All box plots show the IQR with minimum = Q1 – 1.5*IQR, and maximum = Q3 + 1.5*IQR and a half violin plot showing data distribution.

We first established an ex vivo culture of EM cells (Fig. 1b(i)). We labelled the EM cells by microinjecting fluorescent dextran into two C1 blastomeres^36,37^. The aVBI^24–26,37^, which contains both ectoderm and mesoderm layers, was dissected and cultured on a fibronectin-coated dish. As the ectoderm adhered and spread, EM cells also radiated from the mesodermal tissue at the centre of the explant (Fig. 1b(ii)). To check whether these EM cells developed normally, we compared their relative emergence time from explants from embryos of different stages (14–15, 19 and 21)^38^. We found that stage-14–15 EM cells emerged around 6–8 h later than those from stages 19 and 21, which is roughly equal to the time difference found in embryos grown at 16.5 °C (Extended Data Fig. 1a,b and Supplementary Video 1). High-resolution imaging showed that EM cells within explants migrated in a 2.5-dimensional (2.5D) to 3D environment within the ectoderm (Extended Data Fig. 1c, magenta arrows and Supplementary Video 2). EM cells were often seen moving up and down and making frequent contacts with the surrounding ectodermal cells and the fibronectin substrate underneath, similar to their distribution in vivo (Extended Data Fig. 1d). In our ex vivo model, the EM precursor cells (Fig. 1b(ii), pink cluster), which are a part of the mesodermal tissue, are less motile than the ectodermal layer, and thus remain at the centre of the explant. When EM cells emerge from this mesodermal tissue, they enter the environment of a moving ectodermal layer. These observations suggest that our explant system faithfully recapitulates the natural development of EM cells and the interactions between EM cells and ectodermal cells found in vivo.

Given the striking radial migration pattern of EM cells following the spreading ectoderm, we first asked how EM cells would behave in the absence of the ectoderm. We fragmented the explants into smaller pieces to release EM cells into the surrounding space outside. We refer to them as free EM cells (Fig. 1c and Supplementary Video 3). Free EM cells are much less persistent and less radially directional than native EM cells (Fig. 1d–f and Extended Data Fig. 1e). Free EM cells also present characteristics of low-adhering cells, with round cell bodies and a jumping motion^39,40^, whereas native EM cells move in a more streamlined manner. This is reflected in a higher average speed as well as a higher total turning angle for free EM cells (Extended Data Fig. 1f,g). Shape analysis also showed that free EM cells remained relatively round, with a smaller cell area, whereas native EM cells were more elongated with higher average cell area over time (Fig. 1g and Extended Data Fig. 1h).

**Fig. 2:**
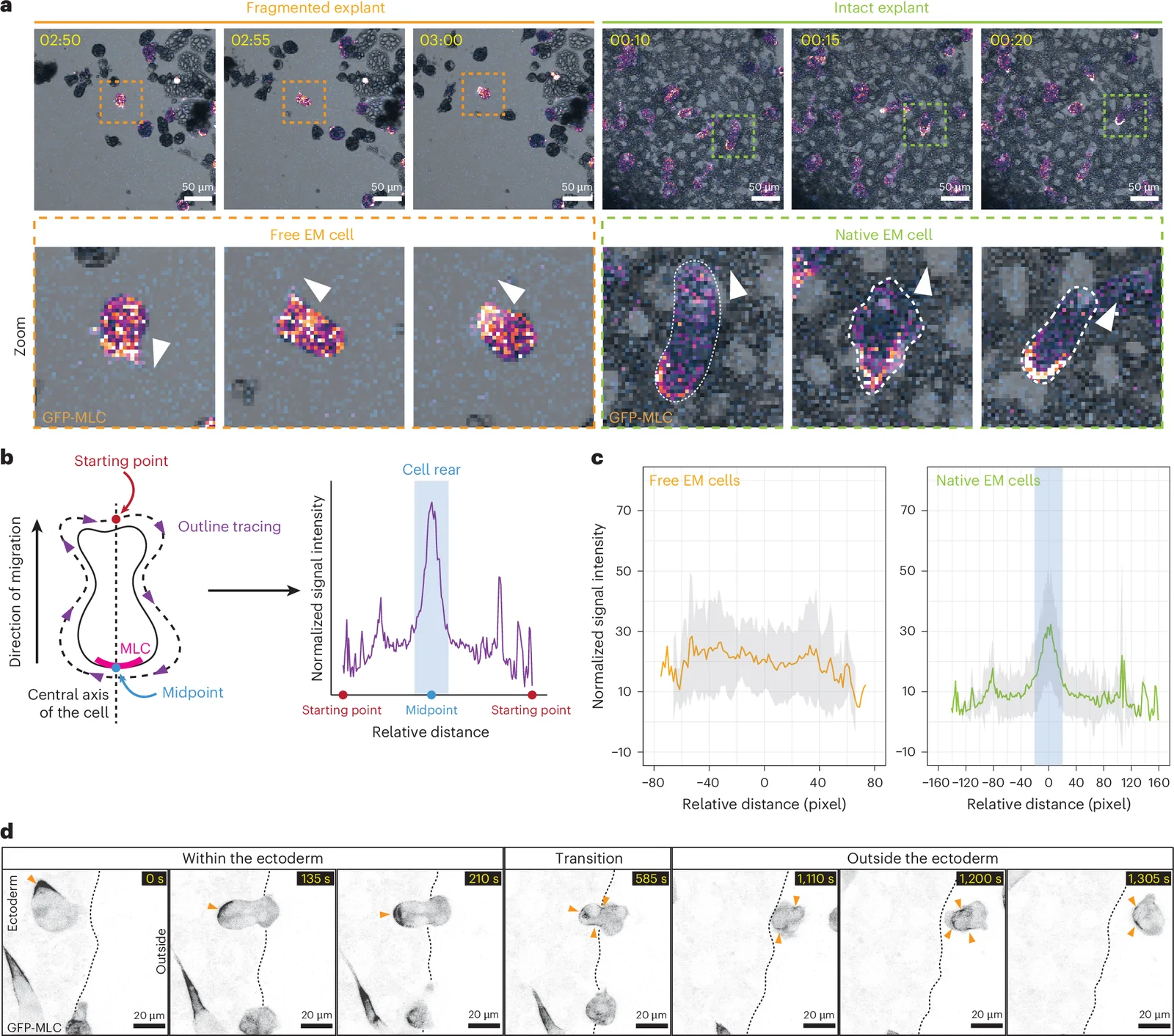
EM cells display a polarized distribution of MLC signal inside intact ectoderm. **a**, Representative images showing the distribution of GFP-MLC signal in free EM cells versus native EM cells. White arrows indicate the direction of the main protrusion. Time in minutes in yellow. Scale bars, 50 μm. See Supplementary Video 4 for details. **b**, Schematic workflow showing how the GFP-MLC signal was quantified. **c**, Average distribution of GFP-MLC signal ± s.d. in free EM cells (*n* = 33 cells) versus native EM cells (*n* = 71 cells). The blue shaded band indicates the cell rear. **d**, Representative image montage showing the transition of the GFP-MLC signal from a polarized distribution inside the ectoderm to a scattered distribution outside the ectoderm. The explant boundary is shown as a black dotted line. Time in seconds in yellow. Scale bars, 20 μm. See Supplementary Video 5 for details.

The difference in shape suggests a change in polarity. Accordingly, we next expressed green fluorescent protein-tagged myosin light chain (GFP-MLC) in EM cells (Fig. 2a and Supplementary Video 4) and found an almost random GFP-MLC distribution in free EM cells, whereas native cells had a clear polarized rearward signal (Fig. 2b–d and Supplementary Video 5).

Overall, these observations suggest a role of the ectoderm in enabling the polarized morphology and the striking radial migration of EM cells ex vivo.

### Artificial compression cannot completely recapitulate native EM cells’ morphology and radial migration

**Fig. 3:**
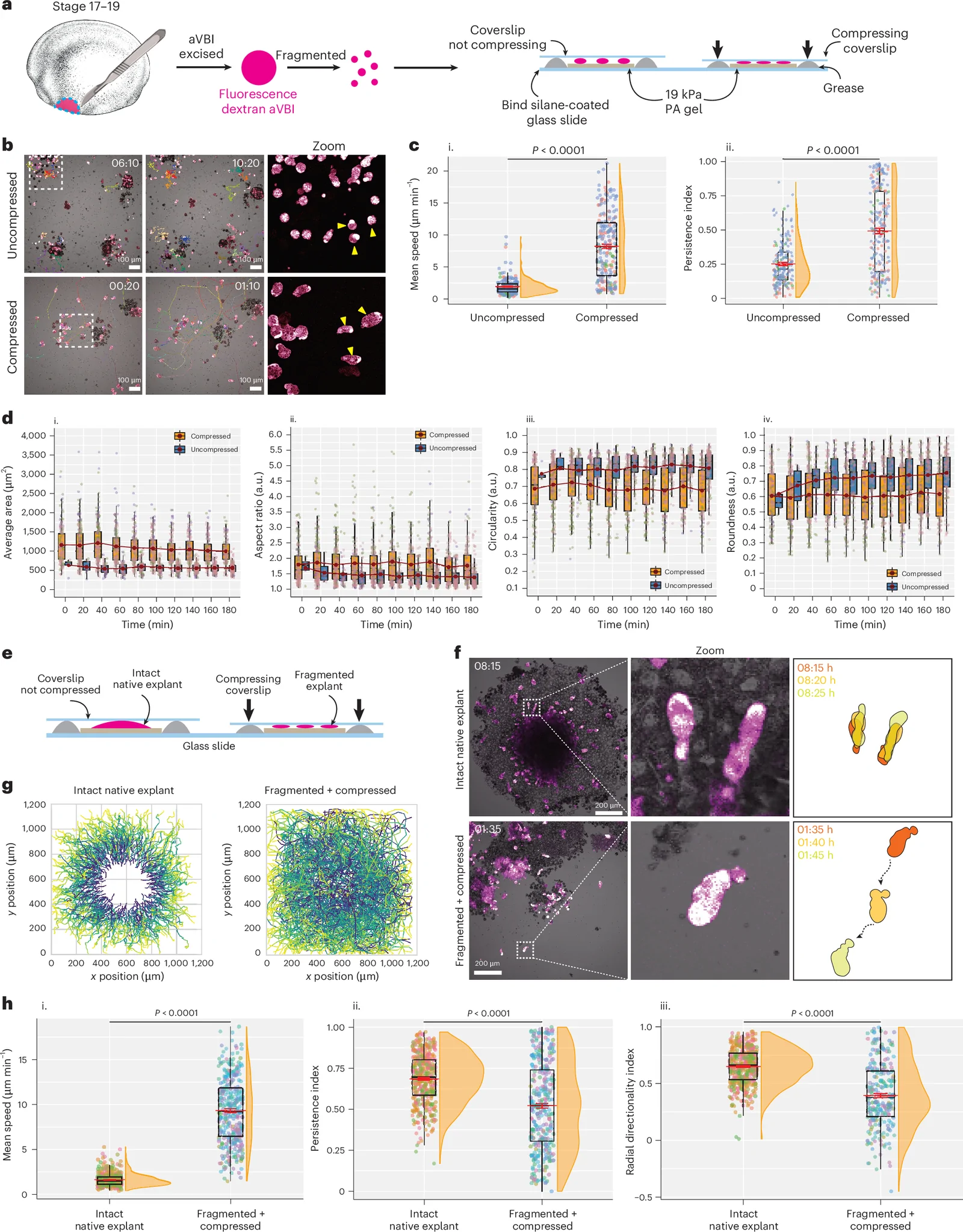
Compression cannot recapitulate native EM cells’ morphology and radial migration. **a**, Schematic workflow showing how EM cells were artificially compressed on polyacrylamide gels. **b**, Representative image montage comparing the different morphologies of uncompressed versus compressed EM cells (magenta). Uncompressed EM cells assume a rounder shape, whereas compressed EM cells are flat and elongated, with a small, round rear end. Scale bars, 100 μm. See Supplementary Video 6 for details. **c**, (i) Box plots comparing the average speed between uncompressed (mean = 1.947 µm min^−1^, *n* = 189 cells) and compressed free EM cells (mean = 8.234 µm min^−1^, *n* = 213 cells). Two-tailed unpaired *t*-test. Mean ± s.e.m. (ii) Box plots comparing the average persistence index between uncompressed (mean = 0.252, *n* = 189 cells) and compressed free EM cells (mean = 0.493, *n* = 213 cells). Two-tailed unpaired *t*-test. Mean ± s.e.m. **d**, Morphology indexes between uncompressed (blue; *n* = 5 to 135 cells) and compressed EM cells (orange; *n* = 111 to 192 cells) over time: (i) average area, (ii) aspect ratio, (iii) circularity and (iv) roundness. On average, compressed EM cells have larger average area and larger aspect ratio (more elongated) than uncompressed EM cells. In contrast, uncompressed EM cells are more circular and rounder than compressed EM cells. These trends were maintained throughout the imaging period. The trend line (red) connects mean values. **e**, Schematic representation of the experiment comparing EM cell migration for normal intact explants and under compressed conditions. In the intact explant condition, the glass coverslip was not pushed down. **f**, Representative images showing the morphologies of native EM cells versus compressed EM cells. Cells from both conditions were more elongated, but their exact morphology and migration behaviour remained different. Compressed EM cells possessed a distinctively small and round rear end, whereas native EM cells lacked this. Compressed EM cells were also a lot faster than native EM cells, as shown in the overlaying colour map. Scale bars, 200 μm. See Supplementary Video 7 for details. **g**, Migration trajectory plots of native EM cells within intact explants versus free compressed EM cells. A lighter colour means further away from the starting point. **h**, (i) Box plots comparing the average speed between native EM cells in intact native explants (mean = 1.635 µm min^−1^, *n* = 414 cells) and free compressed EM cells (mean = 9.323 µm min^−1^, *n* = 335 cells). Two-tailed unpaired *t*-test. Mean ± s.e.m. (ii) Box plots comparing the average persistence index between native EM cells in intact native explants (mean = 0.685, *n* = 414 cells) and free compressed EM cells (mean = 0.522, *n* = 335 cells). Two-tailed unpaired *t*-test. Mean ± s.e.m. (iii) Box plots comparing the average radial directionality index between native EM cells in intact native explants (mean = 0.65, *n* = 414 cells, *N* = 12 embryos) and free compressed EM cells (mean = 0.395, *n* = 247 cells, *N* = 20 embryos). For free compressed EM cells, only those that derived from the same cluster were quantified for their radial directionality indexes. Two-tailed unpaired *t*-test. Mean ± s.e.m. All box plots show the IQR with minimum = Q1 − 1.5 × IQR, and maximum = Q3 + 1.5 × IQR and a half violin plot showing the data distribution.

**Extended Data Fig. 2:**
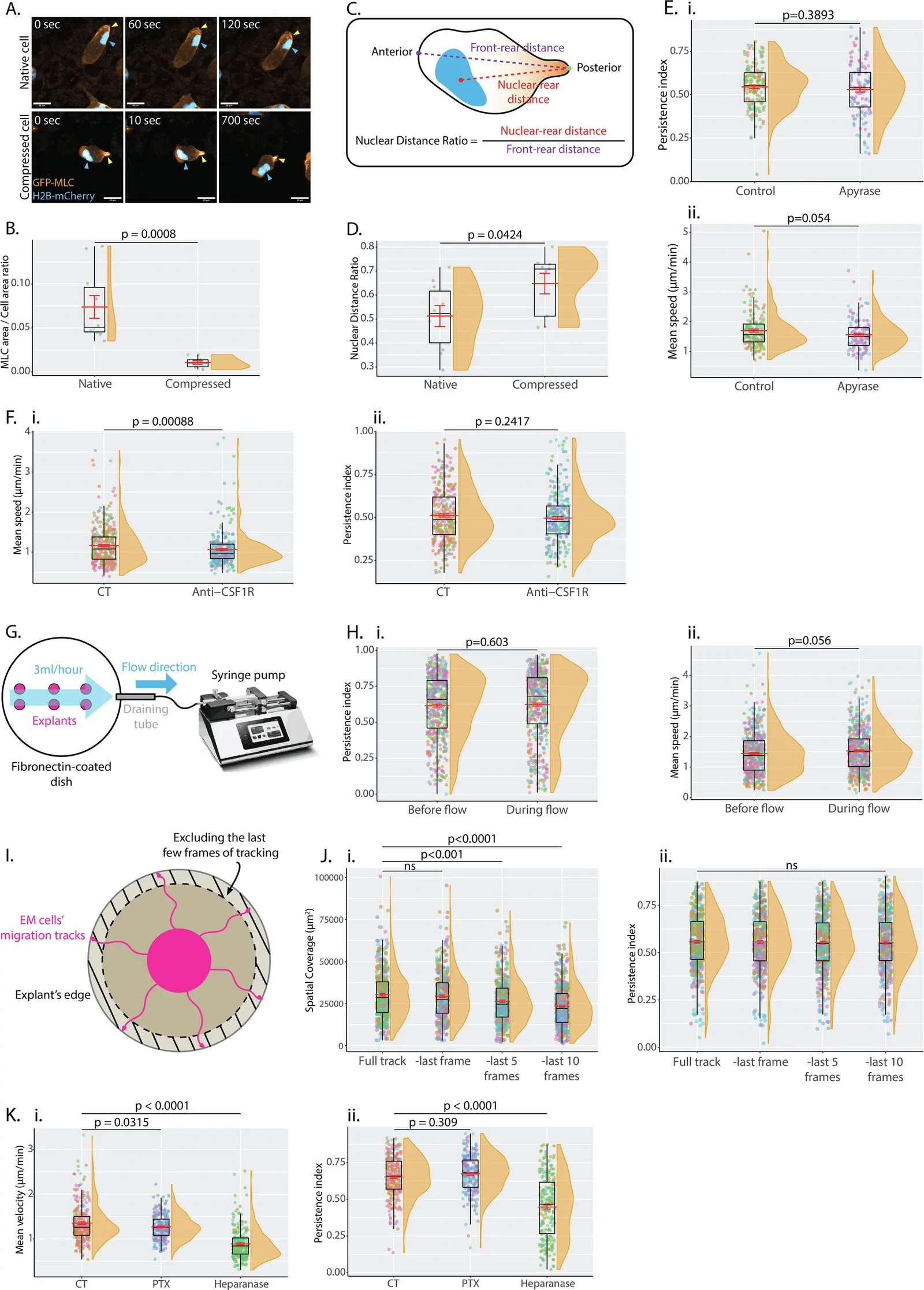
EM cells under compression are morphologically different from native EM cells. Chemotaxis does not play a significant role in EM cell spreading *ex vivo*. **A**. Representative super-resolution image montage comparing the morphology between a native EM cell (top panel) versus an EM cell under compression (bottom panel). Native EM cells have a more elongated oval shape with a broader MLC signal at the rear and a more rearward nucleus (blue) position. Compressed EM cells display a larger front protrusion and a small compact rear with a concentrated MLC distribution and a more front-locating nucleus. Scale bar = 20 μm. Supplementary Video 8. **B**. Box plots comparing the average normalised MLC signal area between native EM cells (Mean = 0.074, n = 10 cells) and free EM cells (Mean = 0.011, n = 9 cells). Two-tailed unpaired t-test. Mean ± SEM. **C**. Schematic representation of how the position of the nucleus was quantified. The distance from the centre of the nucleus to the rear of the cell was measured (red dotted line). The distance from the rear to the furthest point of the main front protrusion (purple dotted line) was measured. The Nuclear Distance Ratio is calculated as a ratio between these two distances. **D**. Box plots comparing the average Nuclear Distance Ratio between native EM cells (Mean = 0.512, n = 10 cells) and free EM cells (Mean = 0.648, n = 9 cells). Two-tailed unpaired t-test. Mean ± SEM. **E**. i. Box plots comparing the average migration persistence index of EM cells in Control explants (Mean = 0.545, n = 150 cells, N = 5 embryos) or in the presence of the ATPase Apyrase (Mean = 0.530, n = 120 cells, N = 4 embryos). Two-tailed unpaired t-test. Mean ± SEM. ii. Box plots comparing the average speed of EM cells in Control explants (Mean = 1.7 μm/min, n = 150 cells, N = 5 embryos) or in the presence of the ATPase Apyrase (Mean = 1.56 μm/min, n = 120 cells, N = 4 embryos). Two-tailed unpaired t-test. Mean ± SEM. Supplementary Video 9. **F**. i. Box plots comparing the average speed of EM cells in Control (Mean = 1.17 μm/min, n = 300 cells, N = 15 embryos), and in anti-CSF1R antibody-treated (Mean = 1.06 μm/min, n = 280 cells, N = 14 embryos) conditions. Two-tailed unpaired t-test. Mean ± SEM. ii. Box plots comparing the average migration persistence index of EM cells in Control (Mean = 0.512, n = 300 cells, N = 15 embryos), and in anti-CSF1R antibody-treated (Mean = 0.497, n = 280 cells, N = 14 embryos) conditions. Two-tailed unpaired t-test. Mean ± SEM. Supplementary Video 10. **G**. Schematic diagram showing the set-up for the flow experiment. The explants were imaged for 3 h before the flow started at a rate of 3 ml/hour for the next 3 h. Detailed descriptions in the Materials and Methods section. **H**. i. Box plots comparing the average migration persistence index of EM cells in the absence (Mean = 0.616, n = 502 cells) and in the presence (Mean = 0.623, n = 502 cells) of a constant flow. Two-tailed unpaired t-test. Mean ± SEM. ii. Box plots comparing the average speed of EM cells in the absence (Mean = 1.446 μm/min, n = 502 cells) and in the presence (Mean = 1.528 μm/min, n = 502 cells) of a constant flow. Two-tailed unpaired t-test. Mean ± SEM. Supplementary Video 11. **I**. Schematic diagram showing the analysis strategy to dissect the contribution of any possible chemotactic effects near the explant’s edge. The persistence index was calculated for the entire migration track, the entire migration track minus the last time point, the last five time points, or the last ten time points (the greyed-out area). **J**. i. Box plots comparing the average spatial coverage index of EM cells calculated from the entire track (Mean = 30375, n = 396 cells, N = 8 embryos), the entire track minus the last time point (Mean = 29482, n = 396 cells, N = 8 embryos), the entire track minus the last five time points (Mean = 26475, n = 396 cells, N = 8 embryos), and the entire track minus the last ten time points (Mean = 23508, n = 396 cells, N = 8 embryos). ANOVA with Tukey’s multiple comparison test. Mean ± SEM. ii. Box plots comparing the average migration persistence index of EM cells calculated from the entire track (Mean = 0.558, n = 396 cells, N = 8 embryos), the entire track minus the last time point (Mean = 0.556, n = 396 cells, N = 8 embryos), the entire track minus the last five time points (Mean = 0.553, n = 396 cells, N = 8 embryos), and the entire track minus the last ten time points (Mean = 0.553, n = 396 cells, N = 8 embryos). ANOVA with Tukey’s multiple comparison test. Mean ± SEM. **K**. i. Box plots comparing the average speed of EM cells in Control (Mean = 1.35 μm/min, n = 260 cells, N = 13 embryos), pertussis toxin (PTX)-treated (Mean = 1.27 μm/min, n = 220 cells, N = 12 embryos), or Heparanase-treated (Mean = 0.886 μm/min, n = 220 cells, N = 11 embryos) conditions. ANOVA with Tukey’s multiple comparison test. Mean ± SEM. ii. Box plots comparing the average migration persistence index of EM cells in Control (Mean = 0.652, n = 260 cells, N = 13 embryos), pertussis toxin (PTX)-treated (Mean = 0.643, n = 220 cells, N = 12 embryos), or Heparanase-treated (Mean = 0.419, n = 220 cells, N = 11 embryos) conditions. ANOVA with Tukey’s multiple comparison test. Mean ± SEM. Supplementary Video 12. All box plots show the IQR with minimum = Q1 – 1.5*IQR, and maximum = Q3 + 1.5*IQR and a half violin plot showing data distribution. ns = non-significant (p > 0.05).

As the native EM cells were more elongated than the free EM cells, we hypothesized that this could be due to the compression exerted by the ectodermal layer. We used a glass coverslip to gently compress free EM cells cultured on a 19-kPa polyacrylamide gel crosslinked with fibronectin^41,42^ (Fig. 3a). Compressed EM cells exhibited a flatter appearance with a large frontal protrusion, and were significantly faster and more persistent than uncompressed cells (Fig. 3b–d and Supplementary Video 6), in agreement with previous reports^43–45^. This prompted us to ask whether compression alone could recapitulate the distinctive radial migration pattern seen in native EM cells. We performed a side-by-side comparison of compressed versus native EM cells (Fig. 3e). We noticed that while native EM cells still had their streamlined shape, compressed cells had the very stereotypical morphology of a migrating amoeboid cell, with a small round rear end and a prominent frontal protrusion (Fig. 3f and Supplementary Video 7)^45–47^. High-resolution imaging showed that although both conditions have a rear GFP-MLC signal distribution, compressed EM cells appeared to have a much more compact GFP-MLC signal, unlike the broader, more spread-out signal seen in native EM cells (Extended Data Fig. 2a,b and Supplementary Video 8). Compressed EM cells often had their nucleus at the front instead of being closer to the back as in native EM cells, which is indicative of increased actomyosin contractility^48–50^ (Extended Data Fig. 2c,d). Compressed EM cells were also significantly faster, possibly due to their exclusive amoeboid mode of migration^51^, but surprisingly, they remained less persistent and less radially directional than native EM cells (Fig. 3g,h(i,ii,iii)).

Overall, these data show that compression alone cannot explain the streamlined morphology and the radial migration behaviour seen in native EM cells, and suggest other factors are at play.

### Ectodermal tissue flow guides EM cells’ directional migration

We next asked whether the radial migration of EM cells could be explained by chemotaxis. Much like at a wound site, we first hypothesized that the edge of dissected explants might release adenosine triphosphate (ATP) to attract EM cells to migrate outwards radially. Treating explants with the ATPase apyrase affects neither the persistence nor the speed of EM cells (Extended Data Fig. 2e(i,ii) and Supplementary Video 9), suggesting that ATP is unlikely to be involved. Another major chemotactic molecule for macrophages is the macrophage colony-stimulating factor (M-CSF or CSF1)^52^. However, treatment with an anti-CSF1 receptor antibody^53,54^ only resulted in a marginal reduction in the overall speed, with no significant change in the persistence index of EM cells (Extended Data Fig. 2f(i,ii) and Supplementary Video 10). This suggests that CSF1 is unlikely to contribute significantly to the observed directional migration of EM cells ex vivo. However, ATP and CSF1 are only two of the many possible secreted chemoattractants^55,56^. So, to tackle the problem in a more general manner, we introduced a constant flow of liquid by slowly draining out the solution at a rate of 3 ml h^−1^ (Extended Data Fig. 2g and Supplementary Video 11). We reasoned that if any secreted chemoattractant molecules were present, they would be washed out, and this should alter EM cells’ migration behaviour. However, we did not detect any significant change in either the persistence index or the mean speed (Extended Data Fig. 2h(i,ii)). To further rule out any possible attraction coming from the explant edge, we reasoned that if the attraction coming from the edge was notable, excluding EM cell tracking near the edge would significantly decrease the overall persistence index (Extended Data Fig. 2i). Excluding the last frame, the last five frames, or the last ten frames of the migration trajectories of all EM cells indeed reduced the overall spatial coverage^57,58^ as expected, but did not affect their overall persistence index (Extended Data Fig. 2j(i,ii)). General inhibition of GPCR signalling, another major chemotaxis pathway^59^, by pertussis toxins (PTX) only marginally reduced EM cells’ mean speed, but again did not change their persistence index (Extended Data Fig. 2k(i,ii) and Supplementary Video 12). Although it is possible that there might be incomplete inhibition, the observed effects are small enough to suggest that chemotaxis is probably not a major player controlling the radial dispersion of EM cells ex vivo.

**Fig. 4:**
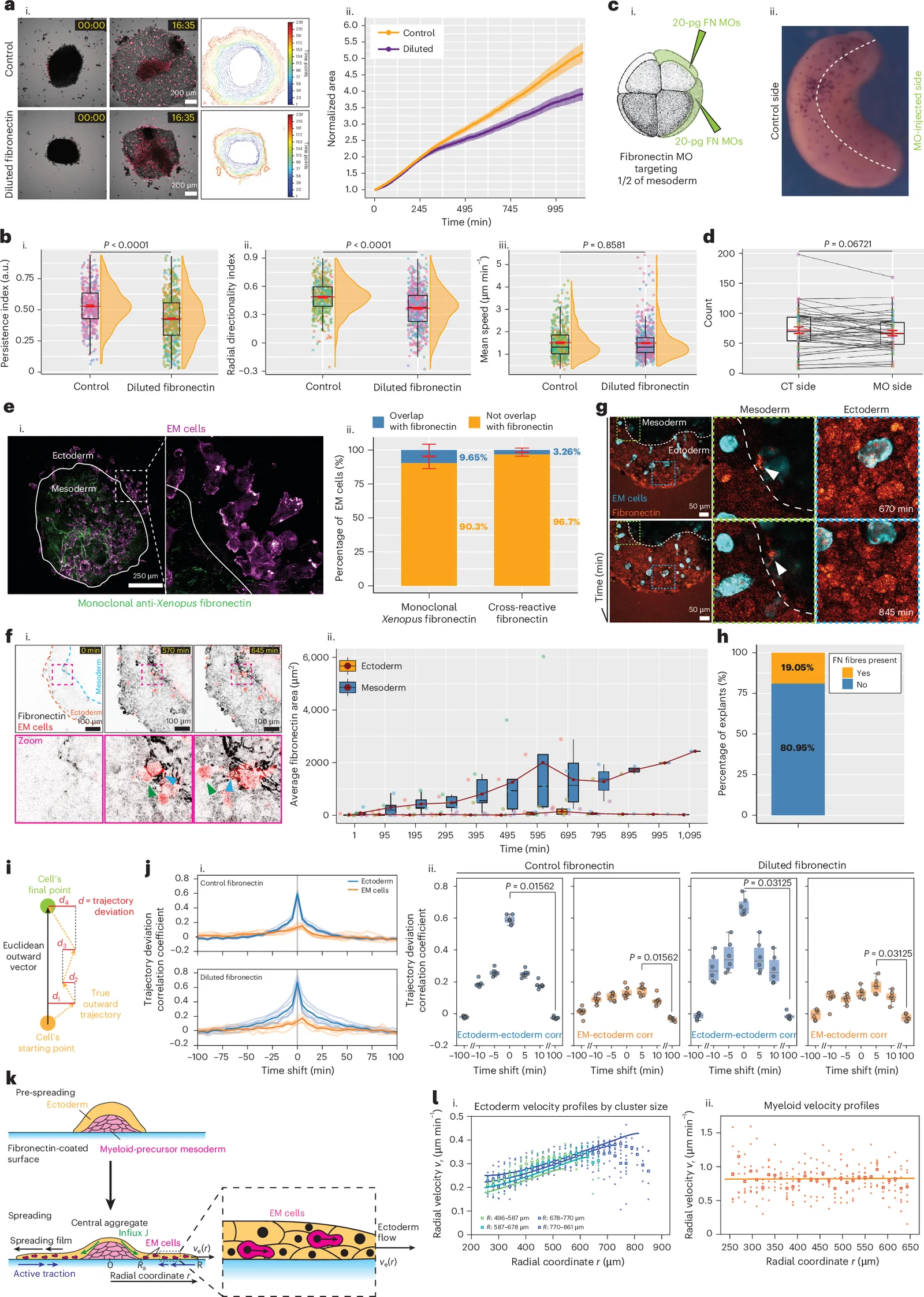
Ectodermal tissue flow guides EM cell directional migration. **a**, (i) Representative image montage comparing a stable (normal fibronectin) versus an unstable (diluted fibronectin) explant spreading. Temporal colour code images show the difference in spreading area between the two conditions. (ii) Line graph showing the normalized spreading area over time. Control *N* = 11 embryos, diluted *N* = 16 embryos. Mean ± s.e.m. Scale bars, 200 μm. See Supplementary Video 13 for details. **b**, (i) Box plots comparing the average persistence index of EM cells between control (mean = 0.529, *n* = 393 cells, *N* = 12 embryos) and the diluted fibronectin condition (mean = 0.428, *n* = 557 cells, *N* = 18 embryos). Two-tailed unpaired *t*-test. Mean ± s.e.m. (ii) Box plots comparing the average radial directionality index of EM cells between control (mean = 0.487, *n* = 453 cells, *N* = 13 embryos) and diluted fibronectin condition (mean = 0.367, *n* = 557 cells, *N* = 18 embryos). Two-tailed unpaired *t*-test. Mean ± s.e.m. (iii) Box plots comparing the average speed of EM cells between control (mean = 0.141 μm min^−1^, *n* = 453 cells, *N* = 13 embryos) and diluted fibronectin condition (mean = 0.139 μm min^−1^, *n* = 557 cells, *N* = 18 embryos). Two-tailed unpaired *t*-test. Mean ± s.e.m. **c**, (i) Schematic diagram showing half of the mesoderm is targeted with fibronectin morpholinos (MO) at the 8-cell stage. (ii) A representative in situ hybridization photo showing the ventral side of an embryo, with fibronectin morpholinos injected into half of its mesodermal tissue. The embryo bends towards the injected side. EM cells are seen as dark coloured spots. **d**, Box plots comparing the number of single EM cells outside of the aVBI between the control (CT, mean = 71.9 cells, *N* = 46 embryos) and fibronectin morpholino-injected side (MO, mean = 66.7 cells, *N* = 46 embryos). Two-tailed paired *t*-test. Mean ± s.e.m. **e**, (i) Representative tile scanning of a whole aVBI explant labelled with a monoclonal *Xenopus*-specific fibronectin antibody (green) and Ruby-LifeAct (magenta) for EM cells. White solid lines indicate the mesoderm region. The zoom image shows that most of the EM cells migrating within the ectoderm have no contact with any the fibronectin fibres, which are mostly concentrating within the mesoderm layer. Scale bar, 250 μm. (ii) Bar charts showing the percentage of EM cells overlapping with any fibronectin fibres in the ectoderm in both monoclonal (9.65% overlap and 90.3% non-overlap, *N* = 3 explants) and cross-reactive (3.26% overlap and 96.7% non-overlap, *N* = 4 explants) antibody conditions. In both cases, most EM cells are not seen associated with any fibronectin fibres in the ectoderm (orange). Mean ± s.e.m. **f**, (i) Representative image montage of aVBI explant live imaging of fibronectin fibres labelled with AlexaFluor555-tagged anti-*Xenopus* fibronectin (black fibres, cyan arrowheads) and fluorescent dextran-injected EM cells (red, green arrowheads). EM cells are only seen moving through and interacting with the fibronectin mesh inside the mesoderm, but not when they are within the ectoderm portion of the explant. Scale bars, 100 μm. See Supplementary Video 18 for details. (ii) Box plots quantifying the amount of visible fibronectin fibres between the mesoderm (orange) and the ectoderm (cyan) for each explant over time (*N* = 5 explants). Box plots show median lines, and red dots represent mean values. Most, if not all, of the fibronectin fibres are confined within the mesoderm and do not extend outwards into the ectoderm. **g**, Representative image montage of an aVBI explant cultured on a Rhodamine-fibronectin-coated surface (red). EM cells are in cyan. The green dotted box highlights the mesoderm area that contains fibronectin fibres (white arrowheads). The cyan dotted box shows the ectoderm area that contains no visible fibronectin fibres. Scale bars, 50 μm. See Supplementary Video 19 for details. **h**, Box plot showing that only 19.05% (*N* = 4 explants) of explants cultured on Rhodamine-fibronectin substrate have visible fibronectin fibres, compared to 80.95% (*N* = 17 explants) of explants that do not have any visible fibronectin fibres. **i**, Schematic diagram outlining the strategy to measure the degree of trajectory alignment between ectodermal cells and EM cells. The trajectory deviation is defined as the difference between the true outward trajectory of a cell (ectodermal cells or EM cells; orange) and its Euclidean outward vector (overall outward trajectory; black). The measurement is designed to exclude any biases that may derive from the natural radial spreading of the explants. **j**, (i) Line plots of the time-shift correlations of trajectory deviations between ectodermal cells and the average local ectodermal cells (blue line) or between EM cells and the average local ectodermal cells (orange line) in both control (*N* = 7 explants) and diluted fibronectin conditions (*N* = 6 explants). A 5-min shift was present between the correlation peak for EM cells versus the correlation peak for ectodermal cells, indicating a 5-min delay in increased trajectory alignment from the EM cells. (ii) Boxplot representation derived from the trajectory line plots showing the average values around the peak for each line (*N* = 7 explants in control fibronectin and *N* = 6 explants in diluted fibronectin). Two-tailed Wilcoxon signed-rank tests. See Supplementary Video 20 for details. **k**, Schematic diagram of an aVBI explant before and during spreading on a fibronectin-coated surface (cyan). The ectodermal tissue (yellow) resides on top, and the mesoderm that contains EM cells (pink) sits underneath. The ectodermal tissue wets and spreads outwards (spreading film arrow) with a radial velocity field *v*_e_(*r*). There is an influx *J* of cells coming from the central aggregate, of radius *R*_a_, which contributes to the spreading of the ectodermal layer, which extends up to a radius *R*. The active traction force (blue arrows) between the ectodermal cells and the substrate is stronger closer to the tissue edge. EM cells migrate within the moving ectoderm and make contact with both the substrate and the surrounding ectodermal cells. **l**, Comparison between model predictions and experimental data for the radial velocity profiles of ectodermal cells (i) and EM cells (ii). (i) The active wetting model (solid lines) fits the experimentally measured radial velocity profile *v*_*r*_(*r*) of ectodermal cells (scatter points) for increasing tissue size from ~250 to ~850 µm, where cells could be experimentally tracked. (ii) The response-saturation model for flow-induced polarization of EM cells (equation 3, solid line) captures the uniform velocity profile of the EM cell population measured experimentally (scatter points), indicating that the EM cell response to ectodermal flow is saturated in the region where cell tracks could be obtained. Parameter values are given in the Methods section. All box plots show the IQR with minimum = Q1 − 1.5 × IQR and maximum = Q3 + 1.5 × IQR and a half violin plot showing the data distribution.

**Extended Data Fig. 3:**
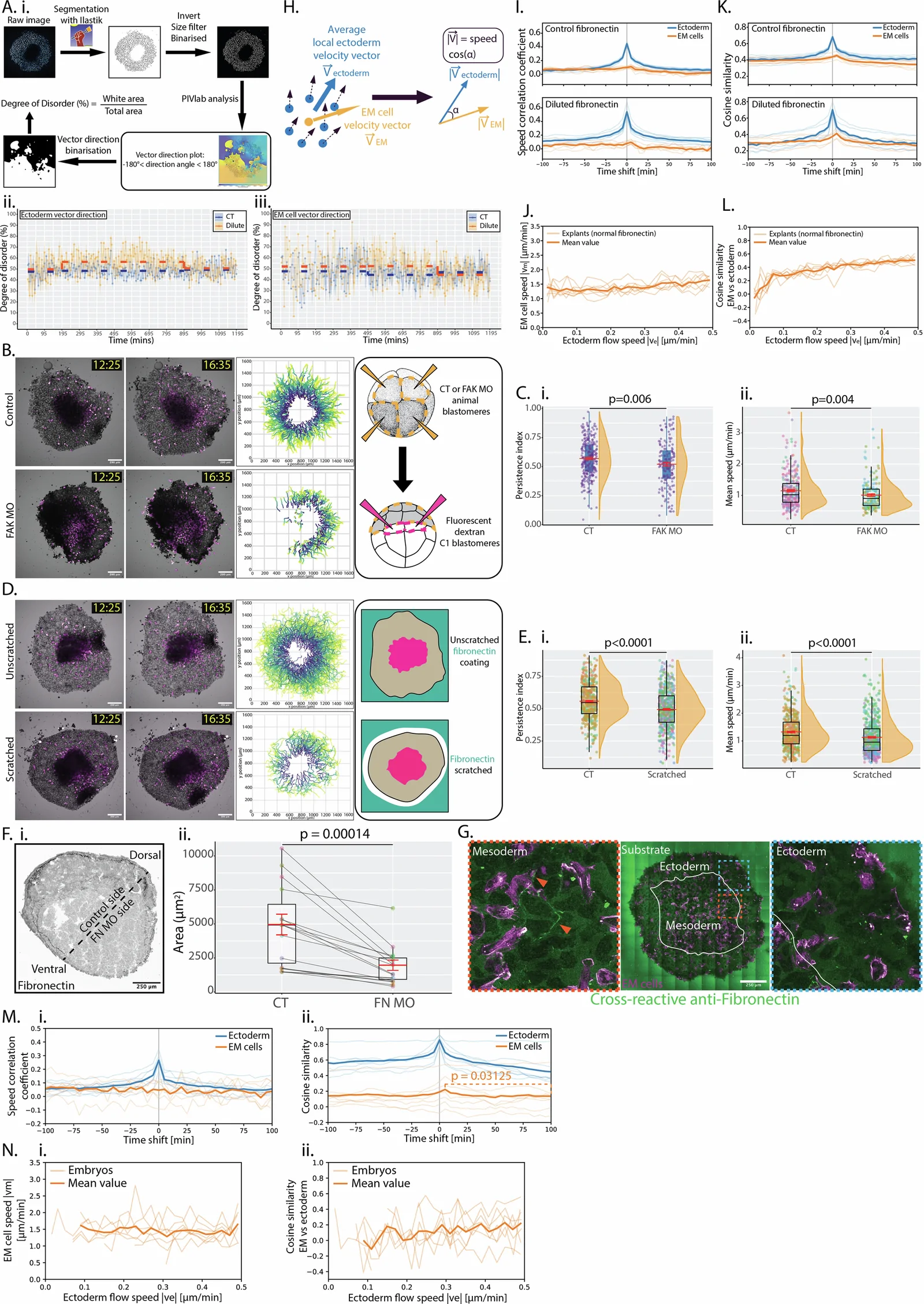
Ectodermal tissue flow influences EM cell directionality. **A**. i. Schematic diagram explaining the strategy to quantify the degree of disorder of vector direction between the control and the diluted fibronectin condition. The same method was applied to quantify EM cell migration. Detailed explanation can be found in the Materials and Methods section. Line graphs comparing the degree of disorder in the migration of ii.) the ectoderm or iii.) EM cells between the control (blue) and the diluted fibronectin (orange) condition. The average degree of disorder (thick blue dotted line) in the control condition remains relatively constant throughout the imaging period for both the ectoderm and EM cells. In contrast, the average degree of disorder (thick orange dotted line) in the diluted fibronectin condition was relatively higher than that of the control condition, and only went down to baseline at 895 min, which coincided with the maximum spreading area of the explant. Supplementary Videos 14 and 15. **B**. Representative images of control versus FAK MO-injected explants coupled with migration trajectory plots. Embryos were double injected with 5 ng of FAK MO to the animal blastomeres and fluorescent dextran to the C1 blastomeres to label the EM cells. Scale bar = 200 μm. Supplementary Video 16. **C**. i. Box plots comparing the average migration persistence index of EM cells migrating in control explants (Mean = 0.521, n = 280 cells, N = 6 embryos) and FAK MO explants (Mean = 0.57, n = 166 cells, N = 6 embryos). Two-tailed unpaired t-test. Mean ± SEM. ii. Box plots comparing the average speed of EM cells migrating in control explants (Mean = 1.147 μm/min, n = 280 cells, N = 6 embryos) and FAK MO explants (Mean = 1.006 μm/min, n = 166 cells, N = 6 embryos). Two-tailed unpaired t-test. Mean ± SEM. **D**. Representative images of control versus fibronectin-scratched explants coupled with migration trajectory plots. Explants were cultured on normal unscratched fibronectin or with fibronectin being scratched around the edge. Scale bar = 200 μm. Supplementary Video 17. **E**. i. Box plots comparing the average migration persistence index of EM cells migrating in control explants (Mean = 0.557, n = 487 cells, N = 17 embryos) and fibronectin-scratched explants (Mean = 0.494, n = 564 cells, N = 21 embryos). Two-tailed unpaired t-test. Mean ± SEM. ii. Box plots comparing the average speed of EM cells migrating in control explants (Mean = 1.328 μm/min, n = 489 cells, N = 17 embryos) and fibronectin-scratched explants (Mean = 1.135 μm/min, n = 564 cells, N = 21 embryos). Two-tailed unpaired t-test. Mean ± SEM. **F**. i. Representative immunofluorescence image showing the presence or absence of fibronectin fibres (black signal) between the control and Fibronectin morpholino-injected side in a cryosection from an embryo. ii. Box plots comparing the area of fibronectin between the control side (Mean = 4956 μm^2^, N = 15 slices) and the fibronectin morpholino-injected side (Mean = 1956 μm^2^, N = 15 slices). Two-tailed paired t-test. Mean ± SEM. Scale bar = 250 μm. **G**. Representative tile scanning of a whole aVBI explant labelled with a cross-reactive fibronectin antibody (green) for bovine fibronectin, which was used to coat the dish, and Ruby-LifeAct (magenta) for EM cells. The white solid line indicates the mesoderm region. The zoom images show fibronectin fibres only present in the mesoderm (orange arrows) but not in the ectoderm, and EM cells do not contact these fibronectin fibres in the ectoderm. Reproducibility was confirmed from 11 separate explants collected from 3 separate female frogs in 2 separate occasions. **H**. Schematic diagram explaining the parameters used for correlation analyses. A Gaussian kernel was centred on a given EM cell. From here, the average velocity vector was inferred for the local ectodermal cells. The correlation between the magnitude (speed) or the angle (cosine similarity) of the velocity vectors of the ectoderm and the EM cells was calculated. For cosine similarity, a value of 1.0 indicates identical directionality, 0.0 indicates orthogonal directionality, and -1.0 indicates opposite directionality. **I**. Line plots of the time-shift correlations between ectodermal cell speeds and average local ectodermal speeds (blue line) or between EM cell speeds and average local ectodermal speeds (orange line) in control (N = 7 explants) and diluted fibronectin (N = 6 explants) conditions *ex vivo*. **J**. Line plot of EM cell speed averaged within bins of local ectoderm speed *ex vivo* (N = 7 explants). The flat profile indicates that increasing local ectoderm speed is not associated with increasing EM cell speed. **K**. Line plots of the time-shift mean cosine similarity (cos(α)) between ectodermal cell directionality and the average local ectoderm directionality (blue line) or between EM cell directionality and the average local ectodermal directionality (orange line) in control (N = 7 explants) and diluted fibronectin (N = 6 explants) conditions *ex vivo*. A peak for the orange line with a 5-minute shift indicates a delayed response of EM cells’ directionality in response to ectodermal cells’ directionality. **L**. Line plot of cosine similarity between EM cell directionality and local ectodermal directionality averaged within bins of local ectoderm speed in normal fibronectin condition *ex vivo* (N = 7 explants). The increasing profile indicates that increasing local ectodermal speed is associated with greater co-alignment of EM cell directionality with local ectodermal directionality. This increase approaches a plateau at higher ectodermal speeds. **M**. i. Speed correlation analysis shows no correlation between the speed of EM cells and the speed of ectodermal cells (orange line) *in vivo*, which is consistent with the *ex vivo* data (N = 7 embryos). ii. Cosine similarity analysis shows a statistically significant correlation between EM cells and ectodermal cells (orange line) *in vivo*. This suggests that there is a degree of alignment between the angleαof direction of EM cells and ectodermal cells. A peak for the orange line with a 5-minute shift indicates a delayed response of EM cells’ directionality in response to ectodermal cells’ directionality (N = 7 embryos). Two-tailed Wilcoxon signed-rank tests. Supplementary Video 21. **N**. i. Line plot of EM cell speed averaged within bins of local ectoderm speed *in vivo* (N = 7 embryos). The flat profile indicates that increasing local ectoderm speed is not associated with increasing EM cell speed. ii. Line plot of cosine similarity between EM cell directionality and local ectodermal directionality averaged within bins of local ectoderm speed *in vivo* (N = 7 embryos). The increasing profile indicates that increasing local ectodermal speed is associated with greater co-alignment between EM cell directionality and the local ectodermal directionality, but this quickly saturates at a certain ectodermal speed. All box plots show the IQR with minimum = Q1 – 1.5*IQR, and maximum = Q3 + 1.5*IQR and a half violin plot showing data distribution.

We next consider the direct interaction between EM cells and ectodermal cells as a possible mechanism. To this end, we treated our explants with heparanase, an enzyme that digests the sugar heparan sulfate that is attached to many transmembrane proteins, which mediates cell–cell interactions and haptotatic signalling^60–63^. Indeed, EM cells in heparanase-treated explants have a significantly slower mean speed and persistence index compared to untreated explants (Extended Data Fig. 2k(i,ii) and Supplementary Video 12). This suggests that direct interactions between EM cells and ectodermal cells may be important. We then hypothesized that the outward flow of the spreading ectodermal tissue could play a role. To test this, we plated aVBI explants on a low-fibronectin surface^64^. In this condition, the ectoderm struggled to stably spread and exhibited frequent retractions and slippages, which resulted in a smaller normalized spread area over time compared to the control (Fig. 4a(i,ii) and Supplementary Video 13). We quantified the degree of disorder caused by the reduced fibronectin using Ilastik^65^ and particle image velocimetry^66,67^ (Extended Data Fig. 3a(i,ii,iii) and Supplementary Videos 14 and 15). Indeed, both ectodermal tissue and EM cells displayed a more disordered flow field in the low-fibronectin condition compared to the control, represented by a higher average line (the orange dotted line compared to the blue dotted line) in both plots (Extended Data Fig. 3a(ii,iii)). Importantly, EM cells in the low-fibronectin condition became less persistent compared to the control, but their migration speed was not affected (Fig. 4b(i,ii,iii)), suggesting that lowering fibronectin did not directly affect the capacity of EM cells to migrate. We also disrupted ectodermal spreading by either inhibiting focal adhesion kinase (FAK)—a key cell–matrix adhesion molecule with a translation-blocking morpholino—or by scratching away the fibronectin around the explant. All of this resulted in a decreased persistence index, while also decreasing the average speed of native EM cells (Extended Data Fig. 3b–e and Supplementary Videos 16 and 17). These data suggest that destabilizing the ectodermal flow impacts the directional migration of native EM cells.

One hypothesis for how the ectodermal flow could influence EM cell migration is through the remodelling of fibronectin. We first depleted fibronectin in one-half of each embryo using a morpholino and compared the number of dispersed EM cells in the control and injected sides (Fig. 4c(i)). Embryos bent towards the injected side, similar to those injected with fibrillin morpholinos^35,68^ (Fig. 4c(ii)). Pairwise comparison between the two sides showed no statistically significant difference in the number of EM cells (Fig. 4d), suggesting that fibronectin is not required for in vivo EM cell dispersion. This is consistent with previous studies showing immune cells can still migrate even when focal adhesions cannot be formed^69,70^. We confirmed the efficacy of the knockdown using immunofluorescence (Extended Data Fig. 3f(i,ii)). Although these observations indicate that EM cells’ dispersion is unlikely to require fibronectin in vivo, we nevertheless examined the idea of fibronectin remodelling by the ectoderm ex vivo. We fixed and stained aVBI explants with a *Xenopus*-specific anti-fibronectin antibody to assess whether the ectoderm could remodel endogenous fibronectin (Fig. 4e(i)). We found that the endogenous fibronectin was located exclusively within the mesodermal layer with virtually no overlap with EM cells in the ectoderm region (Fig. 4e(ii)). We next stained the explants with a cross-reactive antibody that recognizes the exogenous bovine fibronectin used for coating (Extended Data Fig. 3g). The entire surface was homogeneously stained except for only a few fibronectin fibres located exclusively in the mesoderm, which has already been described as a site of fibronectin remodelling^71^ (Extended Data Fig. 3G). Most of the EM cells do not colocalize with any anti-fibronectin signal (Fig. 4e(ii)). We then performed live cell imaging using a fluorescently labelled *Xenopus*-specific fibronectin monoclonal antibody. The antibody, again, showed a strong signal for fibrous fibronectin in the mesoderm of the explants. Labelled EM cells were seen interacting with these fibronectin fibres, but once they left the mesoderm, no fibres were seen associating with these cells (Fig. 4f(i) and Supplementary Video 18). Quantifying the amount of fibronectin within the mesoderm versus the ectoderm shows that virtually all fibronectin fibres remain within the mesoderm, even as time progresses, and the explants expand (Fig. 4f(ii)). We then plated aVBI explants onto a Rhodamine-fibronectin-coated surface to look for signs of exogenous fibronectin remodelling (Fig. 4g). Most explants showed no sign of fibronectin remodelling apart from a small portion of explants that appeared to contain some fibrous structures (Fig. 4g,h, white arrowheads and Supplementary Video 19). However, these fibres were rare and confined to the mesoderm, with little to no interaction with the EM cells. If the ex vivo radial migration of EM cells were to be explained by the ectoderm remodelling of fibronectin, we would have expected to observe a more extensive network of fibres associated with the EM cells, particularly in the ectoderm region. However, this was not the case in all the experiments we performed. We thus conclude that fibronectin does not play a major role in EM cells’ migration in vivo, and that ex vivo, the ectoderm layer does not exhibit any apparent fibronectin remodelling during EM cells’ migration.

To assess the direct mechanical link between ectoderm spreading and EM cell migration, we performed migration correlation analyses between the ectoderm and EM cells ex vivo. We first calculated the trajectory deviation of both ectodermal cells and EM cells relative to their respective Euclidean outward trajectories (Fig. 4i). Unlike raw movement vectors, trajectory deviations take into account the explant’s radial geometry, allowing for an unconfounded assessment of local correlations between EM cells and ectoderm movements (Extended Data Fig. 3h). We performed correlation analyses between ectodermal cells and the local ectoderm averages as an internal control (blue line) and between EM cells and the local ectoderm averages (orange line) (Fig. 4j(i)). As expected for collectively migrating cells, there was a strong correlation between ectodermal cells and their neighbours, represented by a prominent peak (blue line) at 0 min. Interestingly, a smaller but statistically significant peak was also observed for the correlation between EM cells and the neighbouring ectodermal cells (orange line) (Fig. 4j(i,ii) and Supplementary Video 20). There was a shift of ~5 min between the two correlation peaks, suggesting a delay in the response from EM cells to changes in the local ectodermal directionality. This hinted at a causal relationship between local ectodermal flow and EM-cell trajectories. The detected correlation was reproducibly present in both the control and diluted fibronectin conditions, which suggests that this correlation was an innate relationship between EM cells and the ectodermal cells rather than the result of experimental conditions. We found similar behaviour in the cosine similarity (the angles between vectors) between EM cells and the local ectodermal cells, but no correlation was found in the speed component (Extended Data Fig. 3i–l and Supplementary Video 20). To check whether these correlations are also present in vivo, we applied the same analysis pipeline to live embryos. We found no speed correlation but detected a statistically significant correlation in the directionality between the two cell populations (Extended Data Fig. 3m,n and Supplementary Video 21), consistent with previous results. These observations show that ectodermal tissue flow can influence EM cells’ directionality, but not their speed, both ex vivo and in vivo.

We proposed a theoretical model whereby the ectodermal flow polarizes EM-cell migration. We assumed that EM cells are leaving the mesodermal tissue at the centre and moving within an ectoderm that spreads with a velocity field *v*_e_(*r*) (Fig. 4k). We first modelled ectoderm spreading using the theory of active wetting^64,72^. This theory assumes that the tissue behaves as a liquid of viscosity *η* that spreads driven by active traction forces of magnitude *T*_a_ generated by polarized cells at the tissue edge (Fig. 4k). Thus, force balance imposes that

1 $${{\nabla }}\cdot {{\sigma }}=-{T}_{{\rm{a}}}{\bf{p}},$$

where

2 $${{\sigma }}=\eta \left[{{\nabla }}{{\boldsymbol{v}}}_{{\rm{e}}}+{\left({{\nabla }}{{\boldsymbol{v}}}_{{\rm{e}}}\right)}^{\rm{T}}\right]$$

is the viscous stress tensor, and **p** is the ectoderm polarity field, which is 1 at the explant edge and decays to zero towards the interior with decay length *L*_c_. We extend the active wetting model by including an influx *J* of ectoderm cells from the central aggregate, located at *r* ≤ *R*_a_, into the peripheral film defined by *R*_a_ ≤ *r* ≤ *R* (Fig. 4k). Such an influx was observed in previous tissue-wetting experiments^73^ and considered in models of the tissue area dynamics^74,75^. Solving this model (Methods), we predicted that the ectoderm spreads with a radial velocity that increases from the centre towards the edge of the tissue, and that this trend extends with increasing tissue size (Fig. 4l(i), solid lines). Within the area where cells could be experimentally tracked, our experimental data fitted well with our model (Fig. 4l(i), scatter points), suggesting that the spreading of the ectoderm can be modelled as a process of active wetting^64,76–80^ supplemented by a cell influx from the central aggregate.

We next incorporated EM cells into our model. We assumed that EM cells had their own polarity and velocity vectors. Without ectodermal flow, individual EM cells would be polarized randomly, as observed in our data (Figs. 1c and 2a); hence, there would be no net radial polarity of the EM-cell population. However, ectodermal flow would align EM cells’ polarities in the radial direction. Interestingly, unlike the ectoderm, the radial velocity profile of the EM cell population is rather flat (Fig. 4l(ii)). Therefore, the EM cell velocity field *v*_m_(*r*) is not simply proportional to the ectodermal flow *v*_e_(*r*), in line with our previous correlation analysis (Extended Data Figs. 3j and 3n(i)). Furthermore, the cosine similarity between EM cells and the increasing speed of the ectoderm shows a saturating relationship (Extended Data Figs. 3l and 3n(ii)). This led us to hypothesize that EM cells are very responsive to ectodermal flow, and that their response saturates above a speed *v*_s_. To model this behaviour, we take a Hill function of order 1 as the simplest function that accounts for a linear response followed by a saturation:

3 $${{\boldsymbol{v}}}_{{\rm{m}}}\left(r\right)={v}_{\max }\frac{{{\boldsymbol{v}}}_{{\rm{e}}}\left(r\right)}{{v}_{{\rm{e}}}\left(r\right)+{v}_{{\rm{s}}}}$$

where *v*_max_ is the maximal speed of the EM cells, and *v*_e_ = |**v**_e_| indicates the ectodermal flow speed. Thus, by introducing the ectodermal velocity profile *v*_e_(*r*) shown in Fig. 4l(i) into equation (3), we predict the EM velocity profile shown in Fig. 4l(ii) (solid line). Within the range of radial positions where cells could be tracked, this prediction is consistent with the measured EM-cell velocities shown in Fig. 4l(ii) (scatter points).

Overall, by combining ex vivo data, in vivo data and theoretical modelling, our results support the hypothesis that local ectodermal tissue flow can act as a guidance cue for EM cells’ directional migration.

### Local ectodermal flow induces protrusion bias in neighbouring EM cells

**Fig. 5:**
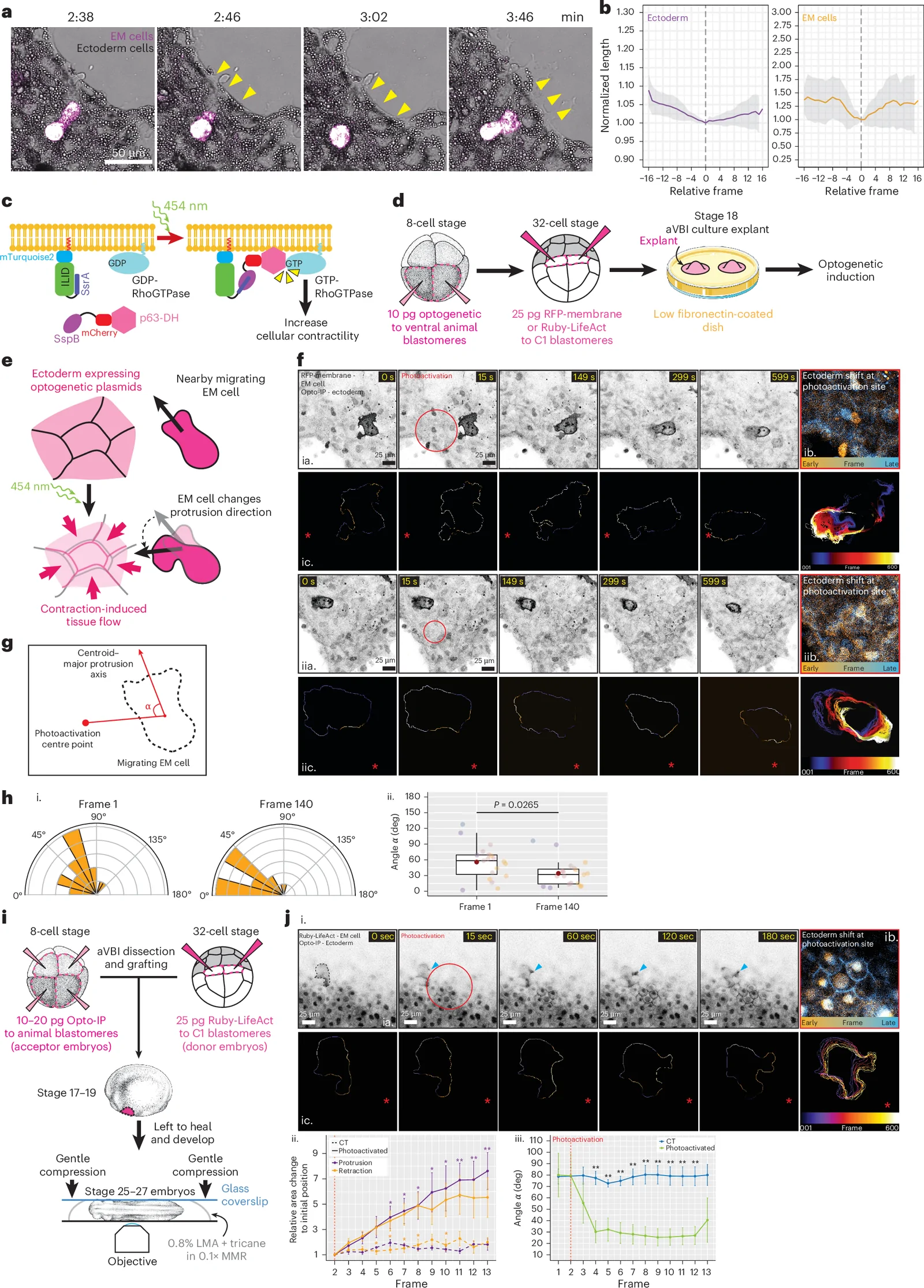
Local ectodermal flow induces protrusion bias in neighbouring EM cells. **a**, Representative image montage showing that a local tissue retraction has caused a neighbouring EM-cell protrusion to collapse (magenta). Yellow arrowheads point to the direction of tissue movement. Scale bars, 50 μm. See Supplementary Video 22 for details. **b**, Line plots showing the relative change in ectoderm distance (purple) and in the EM cell’s protrusion length (orange) over time. Frame 0 is where the ectoderm is at its shortest length, and other frames are relative to this frame. *n* = 22 events, *N* = 18 embryos. Mean ± s.d. **c**, Schematic diagram showing the working mechanism of the Opto-ILID-p63DH (Opto-IP) system. The synthetic protein ILID is connected to mTurquoise2 and anchored to the inner leaflet of the plasma membrane. ILID is also connected to a peptide called SsrA, which has a high natural affinity to a protein called SsrB. SsrB is conjugated to an mCherry-p63DH fusion protein, with p63 being a guanine nucleotide exchange factor. In the inactivated state, ILID sterically binds and blocks SsrA. When ILID is activated by a 454-nm laser, it releases the SsrA peptide and thus allows SsrA to bind SsrB. This brings the conjugated p63DH RhoGEF to the plasma membrane, where it activates the small GTPase protein Rho and induces contraction. **d**, Schematic diagram showing the strategy for mosaically expressing the Opto-ILID-p63DH (Opto-IP) constructs in the ectoderm by injecting only into the two ventral animal blastomeres and labelling the EM cells with either RFP-membrane or Ruby-LifeAct by injecting into the C1 blastomere. Explants were cultured in a low-fibronectin condition to facilitate contraction-induced tissue flow upon optogenetic activation. **e**, Schematic diagram describing the expected effect of a local contraction-induced tissue flow on the protrusion direction of a neighbouring EM cell. Pink arrows denote the direction of the tissue flow towards the centre of the activation area. Black arrows denote the direction of the main protrusion of the EM cell. **f**, Representative image montage showing the effect of local contraction-induced tissue flow by optogenetic biasing neighbouring EM cells’ protrusion dynamics. Induction area near the front (ia.) and rear (iia.) protrusion of the same EM cell. Temporal colour images of an early frame (frame 16; orange) and a late frame (frame 140; blue), showing the shift in the position of ectodermal cells (iia. and iib). Protrusion dynamics analysis (ic. and iic.) of the EM cell showing the direction of protrusion is in the same direction as the contraction-induced flow of the local ectoderm. Blue colour indicates retraction, and red to orange colour indicates protrusion formation. The red asterisks indicate the direction for the optogenetic region. Scale bars, 25 µm. See Supplementary Videos 23 and 24 for details. **g**, Schematic diagram showing the angle *α* between the cell’s major protrusion axis and the centre of the photoactivation region. **h**, (i) Rose plots showing the change in angle *α* from frame 1 (~60°, before photoactivation) to frame 140 (~30°, the point where the EM cell’s protrusion is most aligned with the flow direction) (*n* = 18 cells). (ii) Box plots comparing the average angle *α* between brame 1 (mean = 55.7°, *n* = 18 cells, before photoactivation) and frame 140 (mean = 34.2°, *n* = 18 cells, the point where protrusions are most aligned after photoactivation). Two-tailed Watson–Williams test. Mean values are shown as dark red dots. Box plots show the IQR with minimum = Q1 − 1.5 × IQR and maximum = Q3 + 1.5 × IQR. **i**, Schematic diagram showing the strategy for embryo labelling and mounting for in vivo optogenetic activation. Briefly, Ruby-LifeAct-labelled EM cells were grafted onto embryos expressing Opto-IP constructs in the ectoderm. Embryos were left to heal and grow until at least stage 25, then mounted in 0.8% low melting agarose (LMA) dissolved in 0.1× MMR. A glass coverslip was placed on top and gently pressed down to flatten the embryos before imaging. **j**, (i) Representative image montage showing the effect of local contraction-induced tissue flow by optogenetic biasing of neighbouring EM cells’ protrusion dynamics in vivo. Blue arrowheads point to a new protrusion forming towards the photoactivated area (red circle). Temporal colour images of an early frame (frame 1; orange) and a late frame (frame 13; blue) show the shift in the position of ectodermal cells (ia. and ib). Scale bars, 25 µm. Protrusion dynamics analysis (ic.) of the EM cell showing that the direction of protrusion is in the same direction as the contraction-induced flow of the local ectoderm. Blue colour indicates retraction, red to orange colour indicates protrusion formation. The red asterisk indicates the direction for the optogenetic region. See Supplementary Video 26 for details. (ii) Line graph showing the relative area change (protrusion (purple) and retraction (orange) relative to the first frame before photoactivation) of EM cells over time in both the control (no Opto-IP expression, dotted lines, *n* = 4 cells) and the photoactivated condition (Opto-IP expressed, solid lines, *n* = 6 cells). Two-tailed unpaired *t*-test for each Frame. Mean ± s.e.m. (iii) Line graph comparing the change in angle α from frame 1 to frame 13 between the control (cyan line, *n* = 4 cells) and photoactivated conditions (green line, *n* = 6 cells) (*P < 0.05, **P < 0.01). The red dotted line indicates the beginning of photoactivation. Two-tailed unpaired *t*-test for each frame. Mean ± s.e.m.

Next, we investigate how flow-induced EM-cell polarization happens at the cellular level. We first asked whether ectodermal retraction events could affect the directional migration of EM cells by plating aVBI explants on a low-fibronectin surface. When the ectoderm retracted, the nearby EM cells lost their protrusion and polarity (Fig. 5a and Supplementary Video 22). There was often a slight delay in EM cells’ response before a sudden drop in their protrusion length (Fig. 5b). They then regained their protrusion as the ectoderm spread out again. This suggested that local ectodermal flow could influence EM cells’ directionality by altering protrusion dynamics.

**Extended Data Fig. 4:**
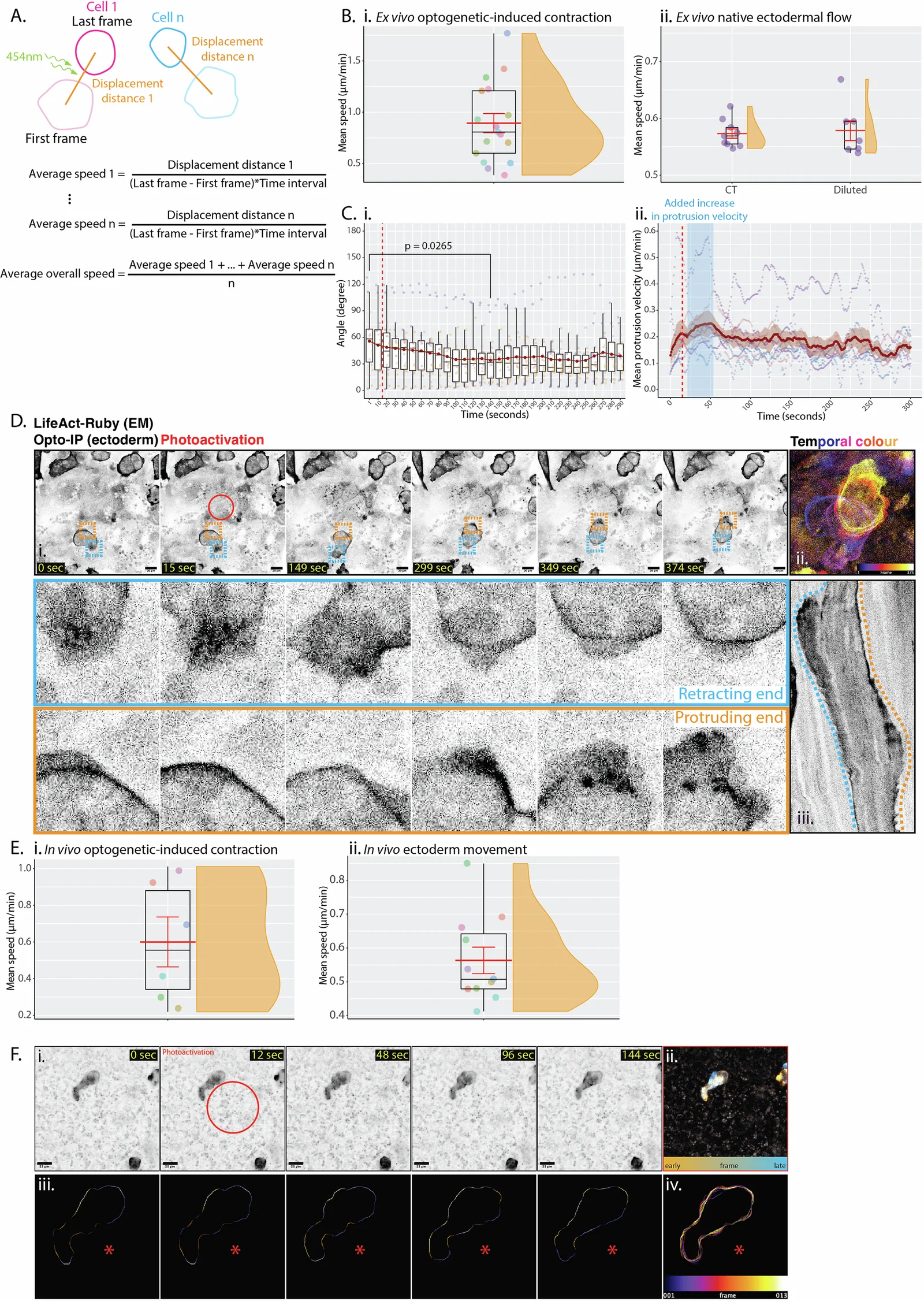
Angle and protrusion dynamic analysis of EM cells in response to contraction-induced tissue flow. **A**. Schematic diagram explaining the strategy to estimate the average contraction speed of ectodermal cells upon optogenetic induction. **B**. i. Box plots showing the average *ex vivo* optogenetic-induced contraction speed (Mean = 0.89 μm/min, n = 17 sites). ii. Box plots showing the average ectodermal flow of *ex vivo* explants in both control normal fibronectin (Mean = 0.57 μm/min, n = 9 explants) and diluted fibronectin (Mean = 0.58 μm/min, n = 7 explants) conditions. Mean ± SEM. All box plots show the IQR with minimum = Q1 – 1.5*IQR, and maximum = Q3 + 1.5*IQR and a half violin plot showing data distribution. **C**. i. Box plot showing the average change in the angle α over time. Red vertical line denotes the point of photoactivation. The mean angle at each time point is connected by a dark red line. The angle α was measured every 10 s. At 140 s, α is at its smallest and is significantly smaller (p < 0.05) than at the beginning. Watson–Williams test (n = 18 cells). ii. Mean protrusion velocity (dark red line) of EM cells over time, showing the increase in protrusion velocity in response to the local contraction-induced tissue flow. Red vertical line denotes the point of photoactivation. Red vertical line denotes the point of photoactivation. Mean ± SD (n = 18 cells). **D**. i. Representative image montage showing an EM cell reverts its polarity and actively forms actin protrusion (visualised with Ruby-LifeAct) to align with the local contraction-induced tissue flow upon photoactivation. The blue box shows the retraction of the actin protrusion opposite the photoactivation region. The orange box shows the actin protrusion facing towards the photoactivation region. ii. Temporal colour code image showing the EM cell reverses its polarity toward the photoactivation region. iii. Kymograph showing the retraction of the protrusion opposite the photoactivation region and the formation of the protrusion towards the photoactivation region. Scale bar = 20 µm. Supplementary Video 25. **E**. i. Box plot showing the average optogenetic-induced contraction speed of ectodermal cells *in vivo* (Mean = 0.6 μm/min, n = 6 sites). ii. Box plot showing the average speed of natural ectoderm movement *in vivo* (Mean = 0.563 μm/min, N = 11 embryos). **F**. Representative image montage showing there is no effect on EM cell’s protrusion bias in control embryos where the Opto-IP system was not expressed and hence no local contraction-induced flow. The red circle indicates the photoactivated area. Temporal colour images of an early frame (Frame 1) (orange) and a late frame (Frame 13) (blue) showing no shift in the position of either the EM cell nor ectodermal cells (i. and ii). Scale bar = 25 µm. Protrusion dynamics analysis (iii.) of the EM cell showing no change in the direction of protrusion even in the presence of blue light activation, but no contraction-induced flow is present. Blue colour indicates retraction, red to orange colour indicates protrusion formation. The red asterisk indicates the direction for the photoactivation region. Supplementary Video 27. All box plots show the IQR with minimum = Q1 – 1.5*IQR, and maximum = Q3 + 1.5*IQR and a half violin plot showing data distribution.

Next, to control tissue flow on demand, we turned to optogenetics. We used a relatively new system called Opto-ILID-p63DH (hereon Opto-IP)^81–83^, which can induce local contractility and therefore a flow of tissue towards the contracting cell (Fig. 5c,d). We hypothesized that a local contraction-induced tissue flow of the ectoderm should bias the protrusion dynamics of neighbouring EM cells (Fig. 5e). Indeed, when a contraction was activated near the side of a forward-moving EM cell, the cell subsequently changed the direction of its main protrusion to head towards the activated area (Fig. 5f(ia) and Supplementary Video 23). We confirmed that the contraction indeed caused a shift in the position of the ectodermal cells (Fig. 5f(ib)) with a speed of ~0.89 μm min^−1^ (Extended Data Fig. 4a), which is of the same order of magnitude as a native spreading ectoderm (~0.6 μm min^−1^) (Extended Data Fig. 4b(i,ii)). Analysis of segmented EM cells clearly shows a change in their protrusion direction^84,85^ (Fig. 5f(ic) and Supplementary Video 23). A contraction-induced flow from behind is often enough to reverse the EM cell’s migration direction (Fig. 5f(iia,iib,iic) and Supplementary Video 24). To quantify the degree of directional change, we measured the angle *α* between the cell’s major protrusion axis and the line connecting the cell’s centroid to the centre of the photoactivation region over time (Fig. 5g). The angle *α* changed from around 60° (before activation), which reflects the lateral position of the photoactivation site relative to the selected EM cell (Fig. 5h(i)), to around 30° at peak contraction (Fig. 5h(i,ii) and Extended Data Fig. 4c(i)), and in many cases, the EM cell’s protrusion became directly aligned with the direction of the contraction-induced flow. We also calculated the average global protrusion velocity of EM cells over time and found an added increase in protrusion activity following photoactivation, suggesting that contraction-induced tissue flow can drive protrusion formation (Extended Data Fig. 4c(ii)). We then asked whether this change in EM cells’ protrusion direction was passive, that is, cells simply being dragged along, or active, that is, cells actively responding to force cues. To test this, we performed the same optogenetic experiment with EM cells expressing the fluorescent filamentous actin reporter Ruby-LifeAct (Extended Data Fig. 4d(i) and Supplementary Video 25). We activated a region of the ectoderm behind a moving EM cell and observed new actin protrusions formed from what was the rear end of the cell (Extended Data Fig. 4d(i,ii,iii)), suggesting an active repolarization mechanism.

Finally, we took our optogenetic system in vivo (Fig. 5i). Upon photoactivation, the ectoderm contracts with an average speed of 0.6 μm min^−1^, in line with the speed of a moving ectoderm in wildtype embryos (Fig. 5j(ia),(ib) and Extended Data Fig. 4e(i,ii)). This contraction biased a pre-existing protrusion and simultaneously induced a new protrusion in a neighbouring EM cell (Fig. 5j(ic) and Supplementary Video 26). Importantly, this response was not observed in the control condition where no contraction-induced flow was present (Extended Data Fig. 4f and Supplementary Video 27). Measuring the area of protrusion extension and retraction in EM cells over time shows no change in both parameters in the control condition (Fig. 5j(ii), dotted lines), while significant increases over time were observed in the photoactivated condition (Fig. 5j(ii), solid lines). The angle of alignment *α* of EM cells was similarly decreased from ~80° to ~30°, but not in control embryos (Fig. 5j(iii), green line and blue line).

Overall, these data suggest that local ectodermal tissue flow can induce active repolarization and protrusion bias in neighbouring EM cells both ex vivo and in vivo.

### Genetically disrupting embryo elongation impairs EM cell dispersion in vivo

**Extended Data Fig. 5:**
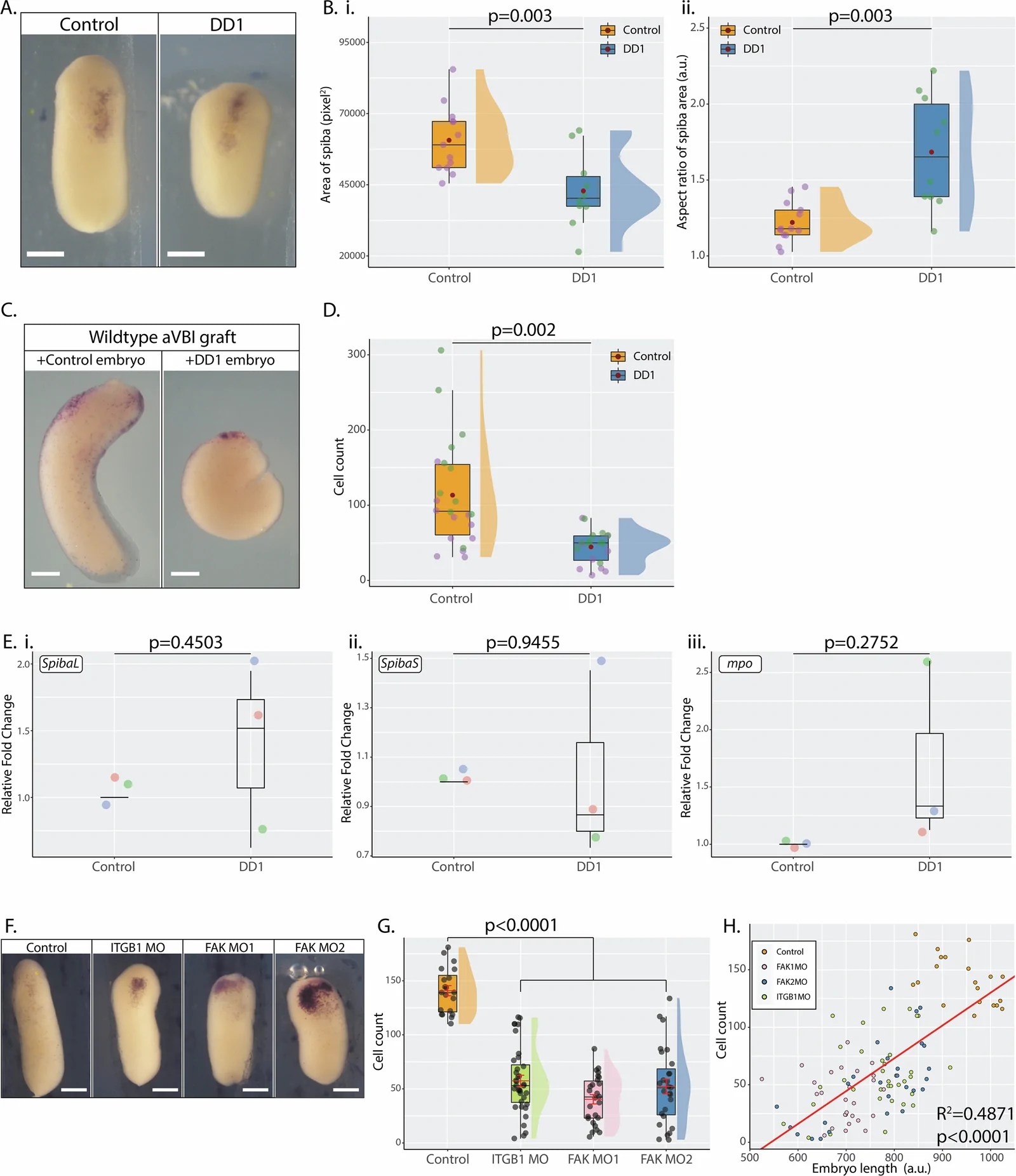
Convergent extension-inhibited embryos by DD1 injection have impaired EM cell dispersion. **A**. Representative ISH images of stage 25 control and DD1-injected embryos stained for the EM cell marker *spiba*. Control embryos are more elongated with a broader *spiba* area, while DD1-injected embryos are shorter with a narrower *spiba* area. **B**. i. Box plots comparing the average *spiba* area between control embryos (Mean = 60625.92, N = 13 embryos) and DD1-injected embryos (Mean = 42826.50, N = 10 embryos). Two-tailed unpaired t-test. Mean ± SEM. ii. Box plots comparing the average aspect ratio of the *spiba* area between control embryos (Mean = 1.221, N = 13 embryos) and DD1-injected embryos (Mean = 1.684, N = 10 embryos). Two-tailed unpaired t-test. Mean ± SEM. **C**. Representative ISH images of stage 27 control or DD1-injected embryos containing an aVBI graft. Control embryos are more elongated than DD1-injected embryos, with more EM cells dispersing throughout the embryo. **D**. Box plots comparing the average number of single EM cells that have escaped the aVBI between control embryos (Mean = 113.4 cells, N = 22 embryos) and DD1-injected embryos (Mean = 44.5 cells, N = 20 embryos). Two-tailed unpaired t-test. Mean ± SEM. **E**. Box plots comparing the relative fold change of the mRNA levels of the *spiba* gene (both i.) *spibaL* (Mean CT = 1, Mean DD1 = 1.36) and ii.) *spibaS* (Mean CT = 1, Mean DD1 = 1.02) and the iii.) *mpo* (Mean CT = 1, Mean DD1 = 1.05) gene between control and DD1-injected embryos. Two-tailed unpaired t-test. **F**. Representative ISH images of stage 25 control, ITGB1 MO, FAK1 MO, or FAK2 MO-ectoderm targeted embryos stained for the EM cell marker *spiba*. **G**. Box plots comparing the average number of single EM cells that have escaped the aVBI between control (Mean ~ 141 cells, N = 20 embryos), ITGB1 MO (Mean ~ 57.4 cells, N = 36 embryos), FAK1 MO (Mean ~ 40.3 cells, N = 26 embryos), or FAK2 MO embryos (Mean = 52.2 cells, N = 27 embryos). ANOVA with Tukey’s multiple comparison test. Mean ± SEM. **H**. Linear correlation analysis between embryo length and the number of EM cells outside of the aVBI across different embryo elongation inhibition conditions. In general, shorter embryos, such as when the ITGB1 or FAK signalling in the ectoderm was inhibited, have fewer EM cells escaping from the aVBI, while control embryos with normal elongation have the highest number of escaped EM cells. R^2^ = 0.4871, p < 0.0001 (two-tailed t-test). All box plots show the IQR with minimum = Q1 – 1.5*IQR, and maximum = Q3 + 1.5*IQR and a half violin plot showing data distribution.

In vivo, embryo elongation, driven by convergent extension along the dorsal side and cellular intercalation on the ventral side^86–91^, creates a flow of tissue^92–94^. We asked whether globally disrupting embryo elongation could affect EM cells’ migration dynamics. We used DD1, a dominant negative Dishevelled mutant of the canonical and non-canonical Wnt signalling pathway, to disrupt elongation by inhibiting convergent extension^95,96^. As expected, DD1 expression results in shorter embryos with a curving dorsal side (Extended Data Fig. 5a). In situ hybridization for the EM cell marker gene *spiba* showed a smaller and thinner EM cell dispersion area compared to the control (Extended Data Fig. 5a,b).

**Fig. 6:**
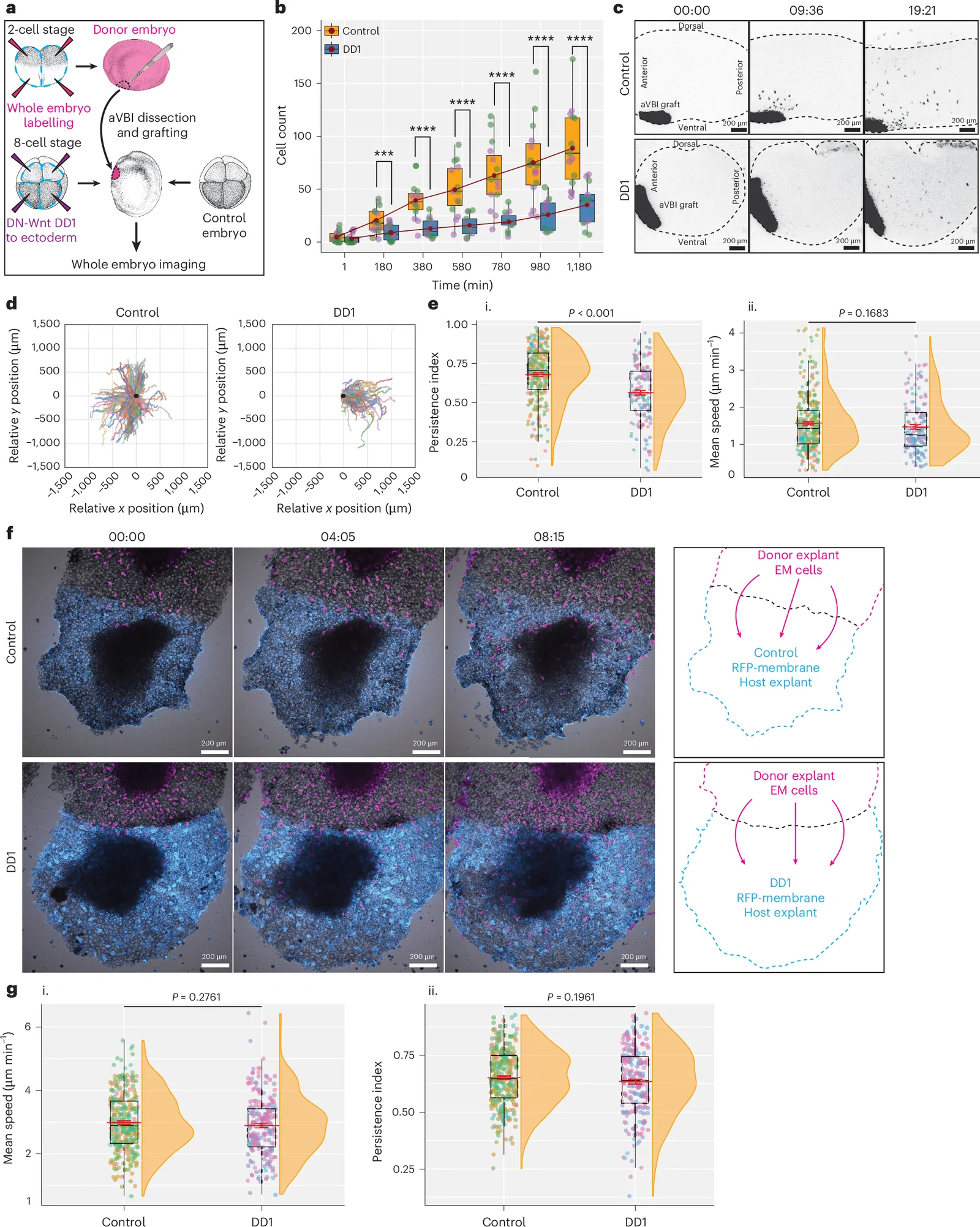
Genetically disrupting embryo elongation by blocking convergent extension impairs EM-cell dispersion in vivo but not ex vivo. **a**, Schematic diagram outlining the grafting strategy. Donor embryos were labelled with fluorescent dextran (magenta). The aVBI explants were dissected and grafted onto either control acceptor embryos or embryos injected with 300 pg of the dominant negative Wnt signalling Dishevelled mRNA (DD1) to the animal blastomeres. Grafted embryos were left to heal and imaged under a confocal microscope. **b**, Box plots comparing the number of EM cells that have escaped from the aVBI in wildtype (orange, *N* = 15 embryos) versus DD1-injected (blue, *N* = 13 embryos) embryos over time at low temporal resolution (time interval = 20 min). Mean values for each time point are shown as dark red dots and are connected by a red line, showing a sharper increase in the wildtype embryos compared to the DD1-injected ones (***P < 0.001, ****P < 0.0001). Two-tailed unpaired *t*-test. Points are colour-coded for different days. **c**, Representative image montage of EM cells (black) migrating within a wildtype embryo (top panel) or a DD1-injected embryo at high temporal resolution (time interval = 9 min). Scale bars, 200 µm. See Supplementary Video 28 for details. **d**, Migration trajectories of EM cells showing the tracks are longer and more directional in wildtype embryos compared to DD1-injected embryos. Note that the overall direction of the trajectories is due to the way embryos were oriented during the imaging section, not due to the presence of any external attractant gradients. **e**, (i) Box plots comparing the average persistence index of EM cells in wildtype (mean = 0.679, *n* = 276 cells, *N* = 8 embryos) and DD1-injected (mean = 0.563, *n* = 148 cells, *N* = 9 embryos) embryos. Mean ± s.e.m. Two-tailed unpaired *t*-test. (ii) Box plots comparing the mean speed of EM cells in wildtype (mean = 1.575 µm min^−1^, *n* = 276 cells, *N* = 8 embryos) and DD1-injected (mean = 1.47 µm min^−1^, *n* = 148 cells, *N* = 9 embryos) embryos. Mean ± s.e.m. Two-tailed unpaired *t*-test. **f**, Representative images of an ex vivo grafting assay showing EM cells (magenta) migrating into a wildtype explant or DD1-injected explant. Scale bars, 200 µm. See Supplementary Video 29 for details. **g**, (i) Box plots comparing the mean speed of EM cells migrating within a wildtype (mean = 2.984 μm min^−1^, *n* = 283 cells, *N* = 7 embryos) or DD1-injected (mean = 2.888 μm min^−1^, *n* = 201 cells, *N* = 5 embryos) explant. Mean ± s.e.m. Two-tailed unpaired *t*-test. (ii) Box plots comparing the average persistence index of EM cells migrating within a wildtype (mean = 0.652, *n* = 283 cells, *N* = 7 embryos) or DD1-injected (mean = 0.635, *n* = 201 cells, *N* = 5 embryos) explant. Mean ± s.e.m. Two-tailed unpaired *t*-test. All box plots show the IQR with minimum = Q1 − 1.5 × IQR, and maximum = Q3 + 1.5 × IQR, and a half violin plot showing the data distribution.

Fluorescence live imaging shows significantly fewer single dispersed EM cells in DD1-injected embryos compared to control across time points (Fig. 6a,b). To rule out the possibility that unlabelled EM cells might have contributed to the observed apparent lower EM cell count in the DD1 embryos, we performed in situ hybridization for *spiba* and confirmed that indeed DD1-injected embryos had a lower total single EM-cell count, even in late-stage embryos (Extended Data Fig. 5c,d). Tracking cells at a higher temporal resolution found that EM cells in control embryos were able to spread further with a higher persistence index compared to the DD1-injected ones, but with similar average speed (Fig. 6c–e and Supplementary Video 28), consistent with our ex vivo data. Importantly, in an ex vivo grafting assay where EM cells migrated into a control or a DD1-injected host explant (Fig. 6f,g and Supplementary Video 29), no difference in their speed or persistence index between the two conditions was observed, suggesting that the effects seen in vivo were likely due to impaired elongation rather than a direct effect of DD1 expression. This effect was also unlikely due to defects in EM-cell development, as expression of the two EM gene markers, *spiba* (both the *spibaS* and *spibaL* homologues) and *mpo*^25,26^, was unchanged in both control and DD1-injected conditions (Extended Data Fig. 5e). Other approaches to disrupt embryo elongation, including depleting Integrin β1^14^ or FAK^97,98^ in the ectoderm (Extended Data Fig. 5f) also resulted in shorter embryos, as previously shown^98–100^, and with fewer single dispersed EM cells (Extended Data Fig. 5g). A correlation analysis showed a linear relationship, where the longer the embryos, the more dispersed the EM cells and vice versa (Extended Data Fig. 5h).

Overall, these data support our hypothesis that EM-cell dispersion in vivo is linked to normal embryo elongation.

### Mechanically restricting embryo elongation impairs EM-cell migration in vivo

**Fig. 7:**
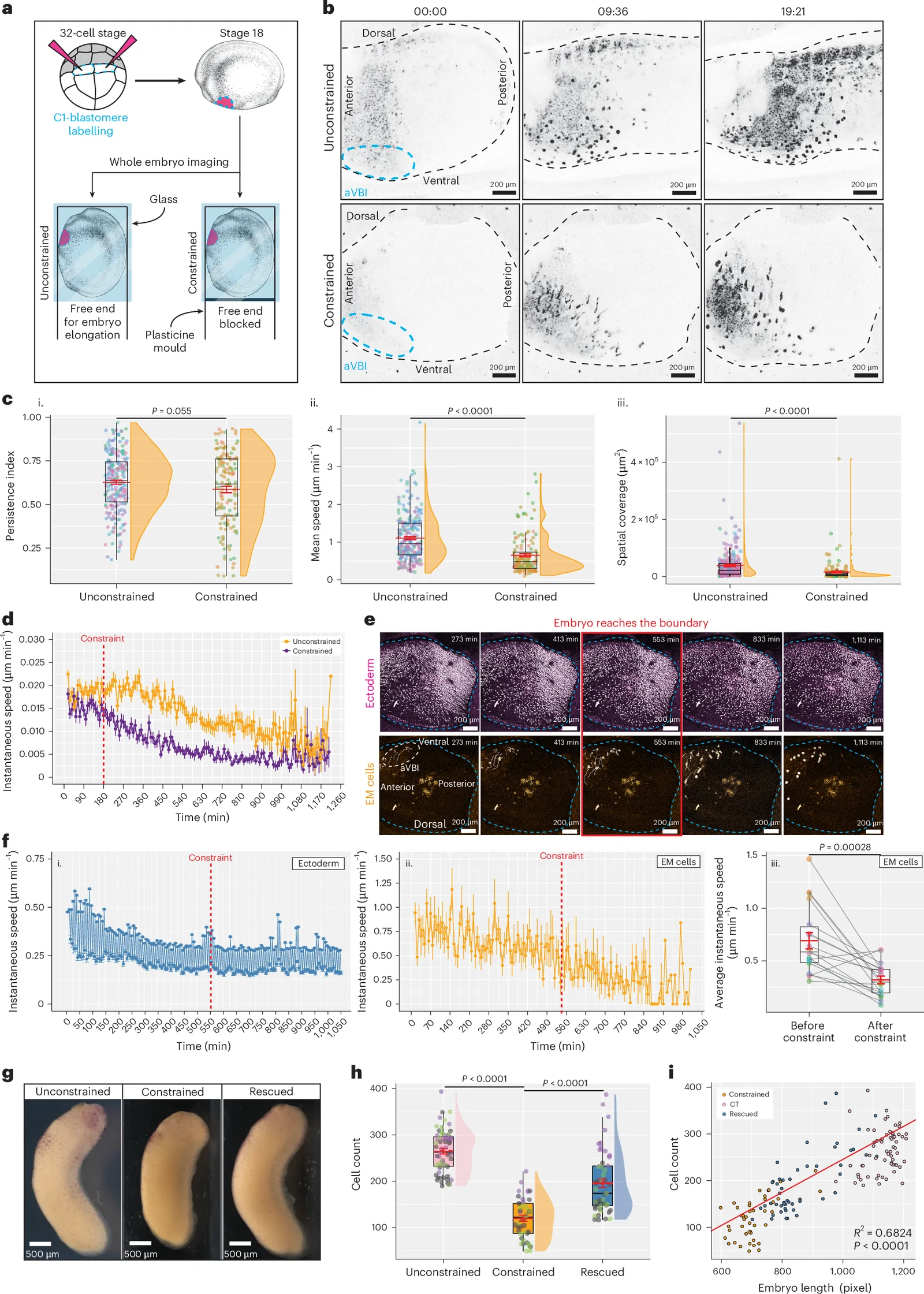
Mechanically restricting embryo elongation impairs EM-cell migration in vivo. **a**, Schematic diagram showing the strategy for constraining embryos. EM cells were labelled by C1-blastomere injection with fluorescent dextran (magenta). Stage-18 embryos were placed in plasticine moulds. For the control condition, the end of the mould was left open for the embryos to extend normally. For the constrained condition, the end is blocked off to stop embryo extension. A piece of glass is placed on top of the embryos to prevent them from slipping out during the imaging process. **b**, Representative image montage of EM cells (black) migrating within a control unconstrained embryo (top panels) or a constrained embryo (bottom panels) at high temporal resolution (time interval = 9 min). aVBI = blue dotted circle. Scale bars, 200 µm. See Supplementary Video 30 for details. **c**, (i) Box plots comparing the average persistence index of EM cells in control unconstrained (mean = 0.627, *n* = 227 cells, *N* = 12 embryos) versus constrained (mean = 0.586, *n* = 144 cells, *N* = 10 embryos) embryos. Mean ± s.e.m. Two-tailed unpaired *t*-test. (ii) Box plots comparing the average speed of EM cells in control unconstrained (mean = 1.105 µm min^−1^, *n* = 227 cells, *N* = 12 embryos) versus constrained (mean = 0.651 µm min^−1^, *n* = 144 cells, *N* = 10 embryos) embryos. Mean ± s.e.m. Two-tailed unpaired *t*-test. (iii) Box plots comparing the average spatial coverage of EM cells in control unconstrained (mean = 38,781.45, *n* = 227 cells, *N* = 12 embryos) versus constrained (mean = 16,043.97, *n* = 144 cells, *N* = 10 embryos) embryos. Mean ± s.e.m. Two-tailed unpaired *t*-test. **d**, Line graph showing the change in the instantaneous velocity of EM cells over time between control unconstrained embryos (orange; *N* = 12 embryos) and constrained embryos (purple; *N* = 10 embryos). EM cells in constrained embryos lost their migration ability when embryos were unable to elongate. The red dashed line indicates the point where the constraint occurs. Mean ± s.e.m. **e**, Representative images of a constrained embryo showing the ectoderm tissue flow (magenta) and the migration of EM cells (orange) in vivo. The red box indicates the moment when the embryo reaches the boundary of the mould and starts to be constrained. Scale bars, 200 µm. See Supplementary Video 31 for details. **f**, (i,ii) Line graphs showing the change in the instantaneous velocity of ectodermal cells (i, blue) and EM cells (ii, orange) over time. Both cells slow their speed when the embryo reaches the boundary of the mould. Red dashed lines indicate the points where the constraint occurs. Paired *t*-test. Mean ± s.e.m. (iii) Box plot showing the average instantaneous speed of EM cells before (mean = 0.544 µm min^−1^, *n* = 18 cells) and after constraint (mean = 0.199 µm min^−1^, *n* = 18 cells). Black lines indicate paired data points. Mean ± s.e.m. Two-tailed paired *t*-test. **g**, Representative ISH images of control unconstrained and constrained embryos, and rescued embryos stained for *spiba* mRNA. Scale bars, 500 µm. **h**, Box plots comparing the average number of EM cells outside of the aVBI between control unconstrained (mean = 264 cells, *N* = 63 embryos), constrained (mean = 120 cells, *N* = 52 embryos) and rescued embryos (mean = 196 cells, *N* = 63 embryos). Analysis of variance (ANOVA) with Tukey’s multiple comparison test. Mean ± s.e.m. **i**, Linear correlation analysis between embryo length and the number of EM cells outside the aVBI. In general, shorter embryos, such as when being constrained (orange), have fewer EM cells escaped from the aVBI, rescued embryos (blue) have an intermediate phenotype, and control unconstrained embryos (pink) have the highest number of EM cells outside of aVBI. *R*^2^ = 0.6824. *P* < 0.0001 (two-tailed *t*-test). All box plots show the IQR with minimum = Q1 − 1.5 × IQR and maximum = Q3 + 1.5 × IQR, and a half violin plot showing the data distribution. Source data

**Extended Data Fig. 6:**
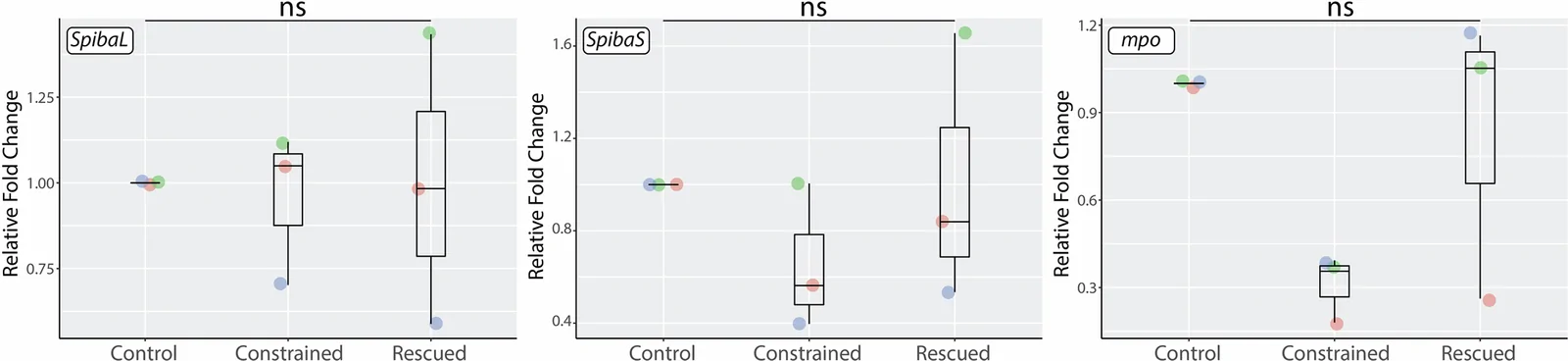
qPCR analysis of unconstrained, constrained, and rescued embryos. Box plots comparing the relative fold change of the mRNA levels of the *spiba* gene (both *spibaL* (Mean CT = 1, Mean Constrained = 0.96, Mean Rescued = 1.00) and *spibaS* (Mean CT = 1, Mean Constrained = 0.65, Mean Rescued = 1.01)) and the *mpo* gene (Mean CT = 1, Mean Constrained = 0.27, Mean Rescued = 0.74) between control unconstrained, constrained, and rescued embryos (n ≥ 9 embryos total per condition, N = 3 replicates). ANOVA with Tukey’s multiple comparison test. All box plots show the IQR with minimum = Q1 – 1.5*IQR, and maximum = Q3 + 1.5*IQR.

Although DD1 inhibits convergent extension mainly on the dorsal side, some level of elongation still occurs on the ventral side. We therefore established a strategy to completely inhibit embryo elongation by mechanically constraining them (Fig. 7a). We found that in the unconstrained condition, EM cells freely dispersed in the embryos as they grew longer. However, in the constrained condition, EM cells initially migrated out from the aVBI but, strikingly, once the embryos reached the boundary of the mould and stopped growing, EM cells abruptly lost their protrusions and stopped migrating altogether (Fig. 7b and Supplementary Video 30). There was a trend towards a lower persistence index in constrained embryos but this was not statistically significant, and was probably caused by the initial directional migration before their total loss of motility (Fig. 7c(i)). However, there is a statistically significant drop in the average speed and spatial coverage (Fig. 7c(ii,iii)), and a faster decline in the instantaneous speed over time (Fig. 7d, purple line) in the constrained condition. To observe the co-migration of the ectoderm and the EM cells, as well as the impact of the constraint, we performed high temporal live imaging of constrained embryos labelled for both cell populations. Initially, as the embryo elongated normally, the ectoderm spread from the ventral to the dorsal side, which was followed by the EM cells dispersing in the same direction (Fig. 7e and Supplementary Video 31). However, when the embryo reached the boundary of the mould (cyan dotted line, red box), the ectoderm stopped and was followed soon after by the EM cells (Fig. 7f(i,ii) and Supplementary Video 31). Quantifying the average instantaneous speed of EM cells before and after constraint shows a significant drop (Fig. 7f(iii)). Importantly, the defective migration phenotype could be rescued if the constrained embryos were released and left to recover, as evidenced by the increase in the number of dispersed EM cells, albeit not to the same level as in the unconstrained condition (Fig. 7g,h). Similar to before, no difference in the expression of the differentiation markers *spiba* (both S and L homologues) and *mpo* was observed between the three conditions, indicating that mechanically constraining embryos probably did not affect the normal development of EM cells within the time frame of the experiment (Extended Data Fig. 6). A correlation analysis showed a positive linear relationship, with unconstrained embryos having the highest number of single cell count, constrained embryos the lowest, and rescued embryos in the middle (Fig. 7i).

Overall, these data support our hypothesis that embryo elongation, and therefore tissue flow, is important for EM cells’ migration and dispersion in vivo.

## Discussion

**Fig. 8:**
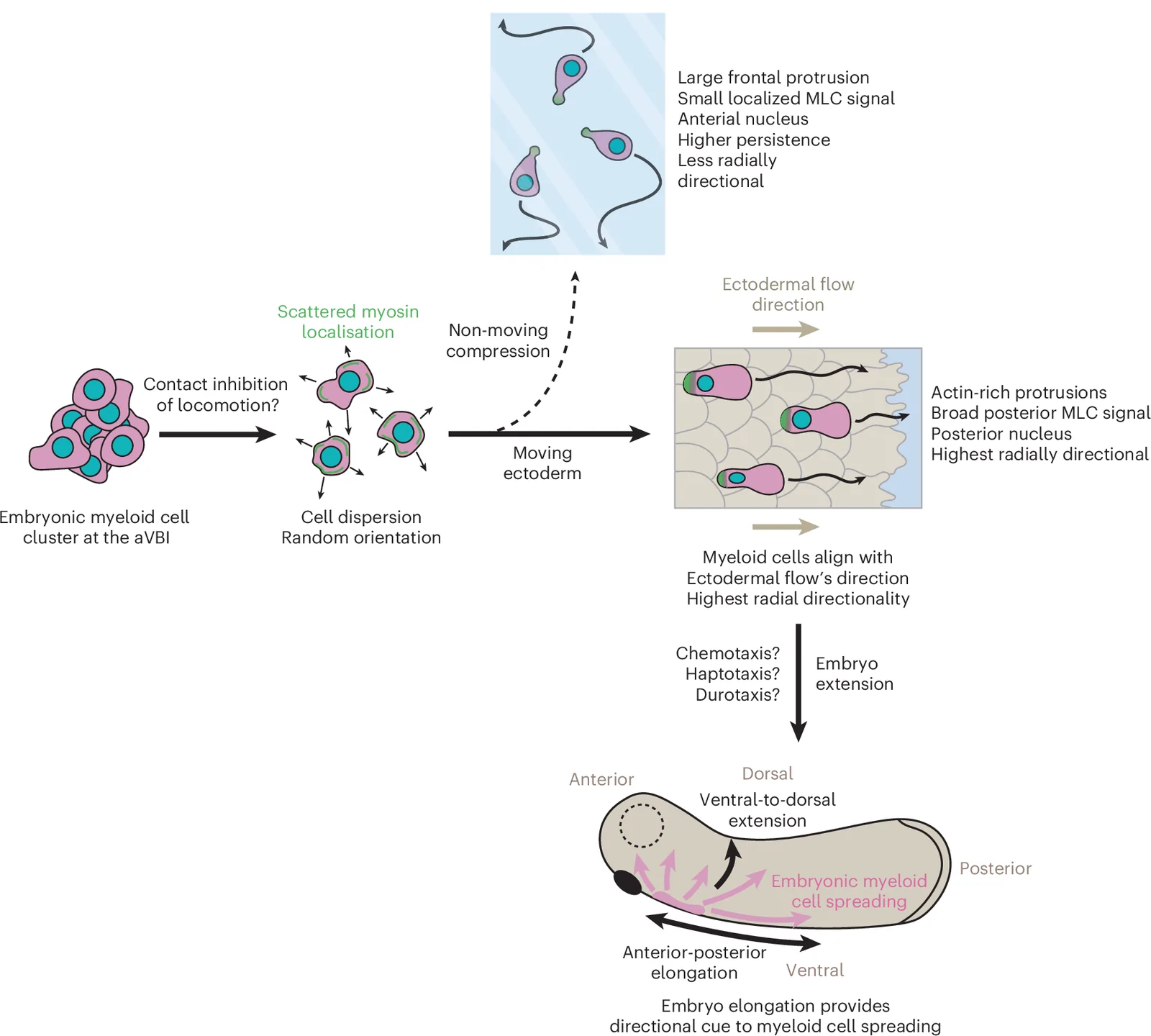
The current working model. EM cells originate as a cluster from mesodermal tissue at the aVBI in embryos. By a yet to be defined signal (perhaps contact inhibition of locomotion), EM cells start leaving the cluster as single cells. Without an ectoderm, EM cells assume a round shape with a random distribution of MLC signal. In the presence of an artificial compression such as a piece of glass, EM cells assume a fast but less radially migrating amoeboid migration phenotype with a large frontal protrusion, anterior-located nucleus, and a small, localized MLC signal at the rear end, which is indicative of a pressure-driven mode of migration. This phenotype does not resemble the phenotypes observed in native EM cells. Within an intact and moving ectoderm, EM cells move more directionally with the highest radial directionality index. They align with the direction of the surrounding ectodermal cells. They have a more elongated morphology, with an actin-rich frontal protrusion and broader and less constrained MLC localization at their rear. The spreading of ectoderm ex vivo reflects the movement during embryo elongation, which provides an added guidance cue (in addition to other potential cues such as chemotaxis, haptotaxis and durotaxis) to direct EM cell dispersion in the developing embryo.

In this Article we provide both ex vivo and in vivo evidence to support the hypothesis that local ectodermal tissue flow can act as a guidance cue for the migration of EM cells. The current working model is summarized in Fig. 8. EM cells start off as a cluster of mesenchymal cells at the aVBI. As the embryo grows, EM cells leave the cluster, perhaps in part driven by contact inhibition of locomotion^101,102^. In the absence of an intact ectoderm, EM cells assume a rounded-up morphology with a scattered myosin signal distribution. In the presence of a non-moving artificial compression, these cells become polarized with a distinctively large frontal protrusion and a concentrated myosin signal at the rear. Their morphology and kinetics, however, do not entirely resemble those seen in native EM cells. Only in the presence of an intact ectoderm can EM cells assume their natural streamlined morphology. The collective movement of the surrounding local ectodermal cells can directly influence EM cells’ protrusion polarization and therefore their direction of migration both ex vivo and in vivo. This flow of tissue is most prominent during embryo elongation, and the directionality of EM cells and the ectodermal cells is correlated with a 5-min delay. Genetically or mechanically interfering with this normal elongation impairs EM cells’ dispersion. Although our experiments were performed in *Xenopus* embryos, a similar challenge arises in the dispersion of embryonic macrophages in other systems. This includes the migration of equivalent immune cells from the anterior lateral plate mesoderm (ALPM) in zebrafish and from the yolk sac in mammals^24–26^. In all these cases, these cells may also employ a similar cell-on-cell migration mechanism. Because of the reliance of EM cells’ polarization and directional migration on the surrounding moving tissue, we coin the term ‘collective-assisted directional migration’ as a subset of cell-on-cell migration to describe this phenomenon.

It is worth noting that EM cells have their own intrinsic motility and do not need a moving ectoderm to be able to migrate. Rather, we propose that the co-movement of the surrounding tissue acts as an additional guidance cue to EM cells, which makes their initial dispersion in embryos more efficient. It is also important to note that our experiments have not specifically considered the co-movement of the endoderm or mesoderm. We cannot visualize beyond two or three cell layers deep in a *Xenopus* embryo, so analysing their correlation in vivo is not possible. Ex vivo culture might be a possible approach; however, accurately preserving the anatomical organization of all three germ layers poses another challenge. Another question is whether passive dragging could explain collective-assisted directional migration. However, the fact that EM cells actively form actin-rich protrusions in response to the local tissue flow, and that there is a 5-min delay in their response, speaks against this being a passive process. This, however, would make an interesting question for future studies.

We also noted that although we did not find a strong contribution of chemotaxis on EM cells’ dispersion ex vivo, we cannot rule out its contribution in vivo. A gradient of secreted or bound chemoattractants^61,103^ could still be present within an intact embryo, or EM cells could create their own path through a self-generated gradient^104–106^. One of the interesting observations is the decrease in both the migration speed and directionality of EM cells when explants were treated with heparanase. Heparanase digests the sugar chain heparan sulfate, which is a typical post-translational modification for cell-surface proteins^107,108^. Heparan sulfate has many different roles, including regulating protein functions^109^, protein–protein interactions through binding to and patterning of morphogens^62,110^, and cell–cell interactions such as during transendothelial migration^60,63^. EM cells seem to respond to the loss of heparan sulfate, which could be an indication of the existence of a haptotactic gradient of unknown molecules bound to the surface of ectodermal cells^111,112^. It is also possible that the loss of heparan sulfate directly affects cellular activities or EM cell–ectodermal cell interactions by disrupting glycosylated transmembrane receptors such as syndecan^109^. Coupled with the fact that EM cells’ migration was altered when the ectoderm was disrupted both ex vivo and in vivo, this supports the hypothesis that physical interactions between EM cells and ectodermal cells are more relevant to their migration in our system.

Although cell-on-cell migration is a widespread phenomenon that has been observed in different model organisms, the exact molecular mechanism for our collective-assisted directional migration remains elusive. In previously described scenarios, cadherin-mediated cell–cell contacts and focal adhesions played an important role in guiding cells along each other^8,9^. In our case, the molecular identity of the EM cell–ectodermal cell interaction is unknown. EM cells appear to use a mixture of amoeboid and mesenchymal migration^113^, evidenced by the presence of both lobopodia-like pseudopods^114,115^ and actin-rich lamellipodia protrusions. This makes identifying a specific cell–cell interaction molecule challenging. However, the idea that particles can align themselves with the motion of surrounding particles has been described and studied extensively in physics^72,116,117^. Hence, given that EM cells are in such proximity to the moving ectodermal tissue, a non-specific friction-like interaction between the two cell populations could be a possible explanation^118,119^. This friction could, perhaps, affect EM cells’ actin retrograde flow, which has been strongly correlated with migration persistence^120^. A recent study also found that the directionality, but not the speed, of the endoderm is correlated with the movement of the mesoderm in zebrafish embryo development^121^. Expanding on this idea, macrophages have been shown to sense tension within the extracellular matrix created by contracting fibroblasts from a distance^122^. So perhaps a moving or contracting tissue can generate a differential tension gradient between cells that EM cells can sense and respond to. Alternatively, the flow of tissue could induce polarization of proteins inside EM cells, which can lead to protrusion formation, similar to how ADIP complexes during neural tube closure in *Xenopus*^123^, and the proteins Fat2 and Lar^124^ during *Drosophila* follicular epithelium development are polarized. Altogether, these examples suggest that collective-assisted directional migration might be conserved in many different biological contexts^121^.

We also noticed that the behaviour of EM cells is reminiscent of neutrophils during transmigration. In the presence of a shear flow, neutrophils roll on the vessel lumen before forming catch bonds with the endothelial tissue using integrins such as α_L_β_2_, α_M_β_2_ or α_4_β_1_^125–129^. They then flatten out and form actin-rich protrusions to search for a weak spot to transmigrate. On the one hand, free EM cells flatten when entering the ectoderm, which could be due to the interactions with the glycocalyx coat and integrin receptors on ectodermal cells, much like those on blood vessels^130^. On the other hand, there are also distinct differences between these two scenarios. Unlike luminal fluid, tissue can respond to changes exerted on it from other cells, so the relationship between EM cells and ectodermal cells is most likely two-way. Neutrophils are also not embedded within a moving sheet of heterogeneous and viscoelastic ectodermal cells, which means the physics that act on them can be very different from what is experienced by EM cells. Therefore, it is possible that some of the molecular mechanisms are shared between EM cells and neutrophils, while others may not be, something that future research could address.

Beyond development, collective-assisted directional migration can also be present in other scenarios, such as during wound healing. As mechanical tension could be transmitted through matrix fibres to attract distant macrophages^122^, a similar mechanism could be at play near the wound edge, where the initial damage chemicals attract distant immune cells, while a mechanical tension propagating between cells could act like a ‘honing-in’ signal to the exact site of injury. During collective cancer invasion, individual cells can align with their neighbours like a nematically active fluid^7^. In the lymph node, dendritic cells^61^, T cells and B cells^131^ have been shown to rely on direct stromal-cell interaction as guidance for their migration. These examples suggest that collective-assisted directional migration might apply to a much broader range of biological contexts.

Overall, our results provide some added insights into how naive immune cells receive and interpret physical cues to navigate the complex environment within embryos. We hope that our findings shed some light on the complex and elusive phenomenon of cell-on-cell migration.

## Methods

Our research complies with all relevant ethical regulations.

### *Xenopus laevis* husbandry and in vitro fertilization

Female *X. laevis* (from NASCO, USA) were preprimed with 100 IU of pregnant mare serum gonadotropin (PMSG; Intervet) before being induced by injecting with 700 IU of human chorionic gonadotropin (Chorulon, Intervet) at least 72 h later. Embryos were collected from at least two female *Xenopus laevis* frogs on the day of the experiment and fertilized in vitro according to the standard protocol.

### Microinjection and labelling of EM cells

Fertilized embryos were dejellied. The EM cells were labelled by microinjecting fluorescent dextran or mRNA into two C1 blastomeres at the 32-cell stage. Embryos were left to heal in FICOL solution for at least 1 h at 18 °C before being transferred to 0.1× Marc’s modified Ringer’s (MMR) solution. The embryos were left to develop at 14.5 °C for ~48 h to reach stage 18. Embryos were selected for correct labelling based on the location of the fluorescence signal at the anterior ventral blood island. The vitelline membrane was then peeled in normal amphibian medium 3/8 (NAM3/8) solution, and explants were dissected.

To specifically target the ectoderm, only the animal blastomeres at the 8-cell stage were injected. Embryos were left in FICOL to recover as described above.

Animal licences were approved by the Animal Welfare and Ethical Review Board (WERB) at University College London and issued by the Home Office, UK.

### aVBI explant system

#### Explant culturing

The aVBI was dissected from stage-18 embryos in NAM3/8 buffer. The explant is composed of the ectoderm and mesoderm layer, which contains the EM cell population. The dissected explants were then transferred onto a dish coated with 10 μg ml^−1^ of fibronectin and containing Danilchik’s For Amy (DFA) solution overnight. Explants were oriented using an eyebrow knife so that the ectoderm layer faced up and the mesoderm layer contacted the fibronectin to mimic how the tissues organized in vivo. A glass coverslip was placed on top of the explant, slightly pushed down to promote the adhesion of the explant and to prevent the explants from curling up due to actomyosin contractility from the ectoderm. Explants were left to adhere for at least 3 h at 14.5 °C before the coverslips were carefully removed. The attached explants could be used for downstream experiments within two days.

For dewetting experiments, explants were plated on a surface coated with 0.5 μg ml^−1^ to 0.6 μg ml^−1^ fibronectin. Subsequent manipulation was as described above.

#### Explant immunofluorescence

Cultured aVBI explants were fixed in 4% formaldehyde in phosphate buffered saline (PBS) overnight at 4 °C. The next day, explants were washed three times in 1× PBS to get rid of excess formaldehyde and were permeabilized by incubation in a solution containing 20 mM glycine and 0.1% Triton-X100 in PBS for 15–30 min at room temperature. The explants were washed three times with 1× PBS, and blocked with 5% bovine serum albumin (BSA) with 0.1% Triton-X100 in PBS for 1 h at room temperature. The explants were then incubated overnight at 4 °C in the same blocking solution, with the added primary antibody at 1 in 50 dilution for the anti-*Xenopus* fibronectin or 1 in 100 dilution for the cross-reactive anti-fibronectin. Next day, the explants were washed three times in PBS and incubated overnight at 4 °C with the secondary antibody, mixed in with the same blocking solution. On the final day, the explants were washed three times with PBS and imaged immediately after.

### Long-term time-lapse

The attached explants were imaged using an upright Leica SP8 confocal microscope or an inverted Axiovert 200M microscope for multi-condition experiments. Explants were cultured in 10 ml of DFA solution and were imaged using a ×10 water immersion lens (HC APO 10×/0.3 W, 506142) or ×20 water immersion lens (HCX APO L20×/0.5 W, 506147). Green fluorescence was excited with a 488-nm laser, and red fluorescence was excited with a 568-nm laser, at 3% power for the ×10 lens and 5% laser power for the ×20 lens, and with 800 digital gains. Images were acquired at 1,024 × 1,024 pixels, averaging at 2, every 5 min for 15–20 h in total.

### Generating fluorescently labelled antibody and live imaging of fibronectin fibres in aVBI explants

To generate the fluorescently labelled version of the anti-*Xenopus* fibronectin antibody (4H2), we first purified the antibody from 5 ml of serum at a concentration of 19 μg ml^−1^ using the affinity chromatography Nab Protein A/G spin kit according to the manufacturer’s protocol. The resulting antibody eluate was buffer-exchanged using Zeba Spin desalting columns. The resulting eluate was then concentrated to ~100 μl using a Pierce Protein Concentrators PES system with 3-kDa cutoff, before proceeding to be labelled with AlexaFluor 555 using the AlexFluor antibody labelling kit (according to the manufacturer’s protocol).

To label fibronectin in vivo and for live imaging in explants, we microinjected around 20 nl of the purified AlexaFluor 555-labelled anti-*Xenopus* fibronectin into the mesoderm of the aVBI region in stage-18 embryos. The embryos were left to incubate with the antibody and to heal at 12.5 °C for 1–2 h. The aVBI was then dissected and plated onto a fibronectin-coated dish and imaged with a ×20 water immersion lens on an SP8 confocal microscope as described above.

### Quantification of EM cell–fibronectin overlapping and fibronectin area

The total EM-cell count outside the mesoderm was quantified. The number of EM cells with any fibronectin fibres overlapping was manually counted as follows:

$$\begin{array}{l}\mathrm{Percentage}\,\mathrm{of}\,\mathrm{EM}\,\mathrm{cells}\,\mathrm{with}\,\mathrm{overlapping}\,\mathrm{fibronectin}\\=\displaystyle\frac{\mathrm{EM}\,\mathrm{cells}\,\mathrm{with}\,\mathrm{overlapping}\,\mathrm{fibronectin}}{\mathrm{Total}\,\mathrm{EM}\,\mathrm{cell}\,\mathrm{count}}\times 100\end{array}$$

$$\begin{array}{l}\mathrm{Percentage}\,\mathrm{of}\,\mathrm{EM}\,\mathrm{cells}\,\mathrm{without}\,\mathrm{overlapping}\,\mathrm{fibronectin}\\=100-\mathrm{Percentage}\,\mathrm{of}\,\mathrm{EM}\,\mathrm{cells}\,\mathrm{with}\,\mathrm{overlapping}\,\mathrm{fibronectin}\end{array}$$

To quantify the area of fibronectin fibres, fibronectin fibres were segmented by manual thresholding. The area of fibronectin visible in the mesoderm and the corresponding area in the ectoderm were measured. Non-specific staining or internalized antibody signal in the live imaging was ignored in the quantified signal.

To quantify for the percentage of explants with visible fibronectin fibres in the Rhodamine-fibronectin experiment, we classified any explant that has any visible fibre-like structures as ‘yes’, and any explant without any fibre-like structures as ‘no’. A percentage is calculated based on this classification.

### Protrusion dynamic assay

Explants with labelled EM cells were cultured in fibronectin at a concentration of 0.5–0.6 μg ml^−1^ to induce dewetting transition. The explants were imaged with the SP8 every 2 min for 20 h using the same set-up as described above. During analysis, only positions with ectoderm retraction events were considered. A line was drawn from a fixed point inside the explant towards the edge of the ectoderm, and this distance was measured. The frame where the ectoderm retracted the furthest was considered frame 0, and the distance was tracked for at least 12 frames before and 12 frames after and normalized to this frame 0. The length of the EM cells near this retraction point was measured and normalized in the same manner.

### Optogenetic experiments

#### Explant optogenetics

For each embryo at the 8-cell stage, two ventral animal blastomeres were injected with 10 pg of Lck-mTurquoise2-iLID-SsrA and 10 pg of SspB-mCherry-p63DH mRNA in FICOL. The embryos were then left to develop into the 32-cell stage before 25 pg of either the membrane-localised red fluorescent protein (RFP-membrane) or Ruby-LifeAct mRNA was injected into the C1 blastomeres. The concentrations of these component mRNAs was optimized to allow for normal embryonic development. One caveat of this approach was that the expression of the optogenetic constructs would be less homogenous. However, the procedure was well-tolerated by the embryos, and we could completely avoid potential cross-contamination if the constructs were also injected into dorsal animal blastomeres, where they could leak into the C1 blastomeres from which EM cells were derived. Because the activation wavelength was in the green channel and we did not have access to a far-red fluorescence protein, we decided to use a red fluorescence protein, either RFP or Ruby, to label the EM cells. We carefully optimized the dose so that the signal from the EM cells was not too dim (which could make it hard to distinguish from the mCherry signal of the Opto-IP constructs) and not so bright that it would saturate any structural detail of the cells. The embryos were left to develop to stage 18 before their aVBI explants were dissected and cultured on 0.5–0.6-μg-ml^−1^ fibronectin substrate overnight. The low level of fibronectin helped increase the contraction induced by the optogenetic system. To perform optogenetic induction, a region of the ectoderm at around 60° angle to the direction of migration of the EM cell was targeted for contractility induction (Zeiss880 confocal mode, ×40 oil lens, wavelength 454 nm, 5% laser power, 1-s interval, started at frame 16 and repeated every five frames until the end). Both the ectoderm and EM cells were imaged with 568-nm wavelength, 5% laser power and a digital gain of 700.

To measure the angle, the centroid of the activated region was first acquired. Then, by using the angle tool, the angle was measured between the line connecting the centre of the activated region and the centroid of the cell (manually estimated) and the line connecting the cell’s centroid and the cell’s major protrusion. This angle was measured every ten frames.

To estimate the contraction speed, ectodermal cells at frame 15 or 16 (just before or just after optogenetic induction occurs) were manually segmented. The choice of frame 15 or 16 depended on whether a cell boundary could be easily identified and was under the assumption that the positions of ectodermal cells at the induction site were still relatively unchanged. The same cells were segmented at the final frame for each movie. The displacement distance between the centroid of each cell between the first frame and the last frame was measured. The average contraction speed was then calculated as

$$\mathrm{Average}\,\mathrm{speed}=\frac{\mathrm{Displacement}\,\mathrm{distance}\,({\upmu{\rm{m}}})}{\left(\mathrm{Last}\,\mathrm{frame}-\mathrm{First}\,\mathrm{frame}\right)\times \mathrm{Time}\,\mathrm{interval}\,({\rm{s}})}$$

#### In vivo optogenetics

For in vivo optogenetics, we injected 20 pg of the Opto-IP mRNAs into all four animal blastomeres. For each embryo, we grafted a Ruby-LifeAct-labelled aVBI explant from a donor embryo to ensure no cross-contamination. Embryos were left to develop to around stage 26–28, when EM cells have migrated out from the aVBI. We then laid the embryos onto their side and mounted them in 0.8% low melting agarose dissolved in 0.1× MMR mixed with tricaine (0.24 mg ml^−1^) in a glass-bottomed dish. A square glass coverslip was quickly placed on top and gently pressed down. To perform the optogenetic induction, a region next to the target EM cell was activated. A *z*-interval of 1 μm was used to ensure EM cells in the deeper tissue layer were captured. Imaging conditions are as follows: Zeiss880 confocal mode, ×40 oil lens, wavelength 454 nm, 10% laser power, consecutive interval, started at frame 2, and repeated every frame until the end. Both the ectoderm and the EM cells were imaged with 568-nm wavelength, 5% laser power, and a digital gain of 700.

### Super-resolution imaging

#### Single images

Explants with EM cells labelled with Ruby-LifeAct or GFP-tagged MLC and/or H2B-mCherry and/or ectoderm labelled with RFP-membrane were imaged using a Zeiss 880 instrument with an Airyscan confocal microscope with ×40 oil immersion lens, *z*-stack at 1-μm interval, every 15 s for 10–15 min.

#### Tile imaging

The bounding grid of each explant was determined and defined using the Tile Scan option in Zen Black. Each scanned image was imaged with a ×40 oil immersion lens at the optimal dimension determined by the Fast Airy scan mode, an optical zoom of 2, and a *z*-interval of 5 μm with 10% overlap. Images were deconvoluted and stitched with Zen Black.

### Outline protrusion dynamic analysis

EM cells were segmented by training the Fiji plugin Labkit. Smaller debris was filtered out by thresholding by size. The segmented images (called the cytoplasm) were converted to binary and then eroded by 1 pixel (called ‘eroded’). The eroded images were subtracted from the cytoplasm images to generate the outline of the cells (called the ‘membrane’). The cytoplasm and membrane images were fed into the Fiji plugin ADAPT, where the protrusion dynamics were detected and analysed. The overall protrusion velocity for each cell was plotted over time.

### Flow experiment

Labelled explants were cultured in a row on a 10-μg-ml^−1^ fibronectin-coated dish containing 10 ml of DFA solution. The explants were left at 21.5 °C for a few hours to allow for the initiation of EM migration. An outlet was connected to one side of the dish. The explants were imaged for ~3 h before the draining of the DFA solution started at a rate of 3 ml h^−1^ until all the liquid was drained and the imaging stopped.

### Migration analysis

Around 20–50 EM cells were manually tracked per explant using the Fiji MTrackJ plugin^132^. Within each set of experiments, the same number of EM cells were tracked for each explant. Only healthy explants with sufficient labelled EM cells were included in the analysis. Healthy explants were defined as not disintegrating or detaching for the majority of the imaging period. Native EM cells were only tracked up until they reached the edge of the explant. Average speeds were calculated. The exported Points data files were fed into the Fiji Chemotaxis Tool plugin to calculate the persistence index according to

$$\mathrm{Persistence}\,\mathrm{index}=\frac{\mathrm{Euclidean}\,\mathrm{distance}}{\mathrm{Accumulated}\,\mathrm{distance}}$$

Euclidean distance is the length of the straight line connecting the first and last points of a track. Accumulated distance is the total path a cell migrated, from start to finish.

For the radial directionality analysis, brightfield images of the explants in the first frame were segmented with the Thresholding function in Fiji before the coordinates of the centroid were determined using the Analyze Particles… function. For every tracked EM cell, we first calculated the magnitude of the initial vector and the final vector according to the following equations:

$$\left|{\bf{V}}_{\mathrm{initial}}\right|=\sqrt{{\left({x}_{\mathrm{centroid}}-{x}_{1}\right)}^{2}+{\left(\,{y}_{\mathrm{centroid}}-{y}_{1}\right)}^{2}}$$

$${x}_{1},\,{y}_{1}:\,\mathrm{coordinates}\,\mathrm{of}\,\mathrm{the}\,\mathrm{starting}\,\mathrm{point}\,\mathrm{of}\,\mathrm{the}\,\mathrm{myeloid}\,\mathrm{cells}$$

$$\left|{\bf{V}}_{\mathrm{final}}\right|=\sqrt{{({x}_{\mathrm{centroid}}-{x}_{2})}^{2}+{({y}_{\mathrm{centroid}}-{y}_{2})}^{2}}$$

$${x}_{2},\,{y}_{2}:\,\mathrm{coordinates}\,\mathrm{of}\,\mathrm{the}\,\mathrm{final}\,\mathrm{point}\,\mathrm{of}\,\mathrm{the}\,\mathrm{myeloid}\,\mathrm{cells}$$

$\left|{\bf{V}}_{\mathrm{initial}}\right|$ and $\left|{\bf{V}}_{\mathrm{final}}\right|$ are the magnitudes of the initial and final vectors.

The radial directionality index was calculated using the equation:

$$\mathrm{Radial}\,\mathrm{directionality}\,\mathrm{index}=\frac{|{{\bf{V}}}_{\mathrm{final}}|-|{{\bf{V}}}_{\mathrm{initial}}|}{\mathrm{Total}\,\mathrm{migration}\,\mathrm{path}\,\mathrm{length}}$$

- If EM cells were radially migrating outwards, $\left|{\bf{V}}_{\rm{final}}\right|$ would increase relative to $\left|{\bf{V}}_{\rm{Initial}}\right|,$ and the radial directionality index would be high.
- If EM cells were not radially migrating outwards, $\left|{\bf{V}}_{\rm{final}}\right|$ would decrease relative to $\left|{\bf{V}}_{\rm{Initial}}\right|$, and the radial directionality index would be low.
- If EM cells were migrating perfectly orthoradially, $\left|{\bf{V}}_{\rm{final}}\right|$ would be the same as $\left|{\bf{V}}_{\rm{Initial}}\right|$, and the radial directionality index would equal 0.

For the total turning angle, the angle between each pair of consecutive vectors was calculated using the equation

$$\cos \left(\theta \right)=\frac{{\bf{v}}_{1}\cdot{\bf{v}}_{2}}{\left|\left|{\bf{v}}_{1}\right|\right|\cdot\left|\left|{\bf{v}}_{2}\right|\right|}$$

where ${\bf{v}}_{1}\cdot{\bf{v}}_{2}$ is the dot product of the two direction vectors, and $\left|\left|{\bf{v}}_{1}\right|\right|$ and $\left|\left|{\bf{v}}_{2}\right|\right|$ are the magnitudes of the two vectors. The total turning angle is the sum of all angles *θ*:

$$\mathrm{Total}\,\mathrm{turning}\,\mathrm{angle}=\sum \theta$$

Spatial coverage was defined as the area of the convex hull formed by the points in the track from each cell. The spatial coverage thus quantifies the extent of the area covered by the moving objects.

For in vivo tracking, embryos were first corrected for drift using the Fiji plugin StackReg and TurboReg before EM cells were manually tracked using MTrackJ.

### Dissecting the contribution of any potential chemotactic effects near the edge of the explants

EM cells were tracked until they reached the edge of the explant. The overall persistence index of the EM cells was compared for four scenarios: taking into account the entire migration tracks, the entire migration tracks minus the last time point, the entire migration tracks minus the last five time points, and the entire migration tracks minus the last 10 time points.

### Shape analysis

EM cells were segmented using the Cellpose_2D_ZeroCostDL4Mic Google Collab^58^ notebook with manual curation to ensure accuracy. Shape indexes, including circularity, roundness and aspect ratio, were automatically calculated.

Circularity was calculated according to

$$\mathrm{Circularity}=4{\rm\pi }\frac{\mathrm{Area}}{{\mathrm{Perimeter}}^{2}}$$

Roundness was calculated according to

$$\mathrm{Roundness}=4\times \frac{\mathrm{Area}}{{\rm\pi }\times {\mathrm{Major}\,\mathrm{axis}}^{2}}$$

Aspect ratio was calculated according to

$$\mathrm{Aspect}\,\mathrm{ratio}=\frac{\mathrm{Major\,axis}}{\mathrm{Minor\,axis}}$$

### MLC signal quantification

A 1-pixel-thick line was drawn manually around the cell of interest, starting from the middle point of the front protrusion, using the Free Hand Selection tool in Fiji. This ensured that the myosin signal would be around the central region of the signal intensity profile (around the 0 relative distance point). Signals were normalized to the lowest signal value for each cell. Multiple cells were quantified, and the signal profiles were then averaged out and plotted.

### Compression experiment

To make polyacrylamide gels, glass slides were first coated with a bind-silane solution overnight. The glass slides were washed three times in 70% ethanol and left to air dry, then 13-mm glass coverslips were incubated with repel-silane solution overnight and left to air dry the next day. For a 19-kPa polyacrylamide gel, 100 μl of solution mixture was made by mixing these solutions in order: 63.4 μl of distilled H_2_O, 20 μl of 40% acrylamide solution, 13.2 μl of 2% bis-acrylamide, 2.4 μl of 6-acrylamidohexanoic acid (AHA), 0.5 μl of tetramethylethylenediamine (TEMED) and 0.5 μl of 10% ammonium persulfate solution. For each gel, 10 μl of the mixture was pipetted onto the bind-silane glass slide before a repel-silane coverslip was placed on top. The gel was left to solidify for 12 min, then the coverslips were gently removed. The gels were briefly washed three times with 100% methanol before being rehydrated three times with 200 μl of 1× PBS, 5 min each at room temperature. For each gel, 50 μl of 1-ethyl-3-(3-dimethylaminopropyl)carbodiimide hydrochloride (EDC) was added and incubated for 30 min at room temperature in the dark. Each gel was washed three times with 1× PBS for 5 min each, before being coated in 100 μl of a solution of 100-μg-ml^−1^ fibronectin in 1× PBS overnight at 4 °C in a humidity chamber. On the day of the experiment, the gels were equilibrated in 100 μl of DFA solution. No PBS should be left on the gel as it is not compatible with *Xenopus* cells.

Labelled aVBI explants were dissected from stage-18 embryos, then chopped into smaller pieces. The chopped explants were transferred into 1 ml of DFA to equilibrate. Excess DFA on the gels was drained. Using a P20 Gilson pipette, 10 μl of the chopped explant in DFA suspension was added onto the gel, then a 22-mm × 22-mm square glass coverslip with grease around the edges was placed on top and gently pressed down.

### In vivo grafting experiment

Acceptor embryos were either wildtype or injected with 300 pg of the dominant negative Wnt signalling protein (derived from a mutant Dishevelled protein called DD1) into four animal blastomeres. Donor embryos were whole labelled with fluorescence dextran by injecting at the 2-cell stage. Both donor and acceptor embryos were at stage 16 before grafting. Acceptor embryos were partially immobilized in plasticine moulds. The aVBI explant was dissected from the donor embryos and grafted onto the aVBI region of the acceptor embryos in NAM3/8. A small piece of glass was placed on top to promote healing. The grafted embryos were left to recover in NAM3/8 at 14.5 °C for at least 1 h before being imaged with the SP8 confocal microscope. The embryos were positioned in channels made from agarose. A layer of 3% methyl cellulose dissolved in 0.1× MMR was carefully pipetted on top to reduce embryo twitching and movement during the imaging period, then 10 ml of NAM3/8 mixed with tricaine (0.24 mg ml^−1^) was carefully pipetted on top. The embryos were left to settle before being imaged with an SP8 confocal system. The imaging set-up was as follows: ×10 water immersion lens, 488-nm laser at 6% laser power, *z*-stack with 25-μm interval, acquired every 9 min for 15–20 h. Only healthy embryos with enough labelled EM cells were included in the analysis. Healthy embryos were defined as those that remained intact for the majority of the imaging period.

### Ex vivo grafting experiment

To recapitulate the same conditions as used in the in vivo grafting experiment, aVBI explants with labelled EM cells were placed adjacent to explants taken from the lateral region of the acceptor embryo on a dish coated with 10 μg ml^−1^ of fibronectin in DFA labelled with RFP-membrane. The explants were left to adhere and spread for at least 2 h before being imaged with the SP8 confocal microscope.

### Embryo constraining experiment

aVBI explants from labelled donor embryos were grafted onto wildtype acceptor embryos as already described. Stage-18 embryos were placed in plasticine moulds with a glass coverslip placed on top to prevent them from slipping out. Unconstrained embryos were left in moulds with a free opening for normal elongation, and constrained embryos were placed in moulds with this end blocked. Embryos were imaged in NAM3/8 with tricaine as described above. Only healthy embryos with enough labelled EM cells were included in the analysis. Healthy embryos were defined as those that remained intact for most of the imaging period. Only healthy constrained embryos that exhibited no significant damage were included in the rescue experiment.

### Colourimetric/fluorescence in situ hybridization and immunofluorescence

Embryos were peeled and fixed in MEMFA solution at 4 °C overnight. The next day, embryos were dehydrated in 100% methanol before being rehydrated in rehydration buffers containing 75%, 50% and 25% methanol. The embryos were washed twice in 1× PBS–0.1% Tween20 before being bleached in a solution containing hydrogen peroxide for 20 min. The embryos were then washed in 1× PBS–0.1% Tween20 and incubated in hybridization buffer for 10 min at room temperature and then 30 min at 65 °C, before being incubated with the digoxigenin (DIG)-modified RNA probe in hybridization buffer at 65 °C overnight. The next day, embryos were sequentially washed with solutions 1, 2, 3 and 4 for 10 min each and solution 5 for 30 min at 65 °C. The embryos were washed twice with maleic acid buffer (MAB) buffer and blocked in MAB–2% Boehringer Mannheim blocking reagent (BMBR) for 1 h at room temperature. The embryos were then incubated with anti-DIG antibody (for colourimetric in situ hybridization (ISH)) or anti-DIG-horseradish peroxidase (POD) antibody (for fluorescence in situ hybridization (FISH)) at 1 in 2,000 dilution for 2 to 4 h at room temperature. The embryos were then washed twice with MAB and left in MAB buffer overnight at 4 °C. The next day, they were washed three times with MAB buffer at room temperature for 10 min each.

- For ISH: embryos were washed three times with alkaline phosphatase (AP) buffer at room temperature before being incubated with AP buffer containing 5-bromo-4-chloro-3-indolyl-phosphate (BCIP) and nitro blue tetrazolium (NBT) (3.5 μl each per 1 ml of AP buffer) for at least 2 h at room temperature or until the colourimetric signal was clearly visible.
- For FISH-immunofluorescence: embryos were incubated in 200 μl of amplification buffer (Perkin Elmer), then Cy-3 or Cy-5 tyramide (Perkin Elmer) solution was added at 1 in 100 dilution and incubate for 20–30 min. The embryos were washed twice in 1× PBS–0.1% Tween20 for 1 h each, then permeabilized in 1× PBS–0.3% TritonX-100 for 15 min. They were then blocked in 1× PBS–5% BSA for 2 h before being incubated with anti-fibronectin antibody at 1 in 200 dilutions overnight at 4 °C. The next day, the embryos were washed three times in 1× PBS–0.3% TritonX-100, then incubated in 1× PBS–5% BSA with the secondary antibody at 1 in 200 dilutions overnight at 4 °C. The next day, the embryos were washed three times in 1× PBS–0.1% Tween20 and could either be imaged directly or cryosectioned.

### Quantifying cell numbers in ISH embryos

Images of ISH embryos from the side were quantified. aVBI was defined as the region near the head just below the cement gland in the embryo. This region was excluded from the analysis. Single EM cells outside the aVBI were counted using the Find Maximum function with an appropriate threshold in Fiji.

To quantify the number of dispersed EM cells between the control and fibronectin morpholino-injected side, only embryos with a midline that could be confidently defined were included in the analysis to avoid potential biases.

### Cryosectioning

The embryos were dehydrated in a 10% sucrose solution overnight at 4 °C. The next day, they were placed in cryosection plastic moulds filled with a 1:1 mixture of OCT and 10% sucrose, oriented to the desired orientation, then transferred to −80 °C to freeze overnight. Sections of 30-μm thickness were collected and adsorbed onto a glass slide. The slides were baked at 37 °C for 1 h or until dry. If the embryos were double-fixed in formaldehyde prior to cryosectioning, an antigen retrieval step was performed before any immunofluorescence staining was performed. To retrieve antigens, the slides were incubated in 1% sodium dodecyl sulfate (SDS) in 1× PBS for 10 min at room temperature then washed with copious amounts of 1× PBS to ensure no SDS remained. The samples were permeabilized in 1× PBS + 0.1% TritonX-100 for 15 min at room temperature and blocked in 1× PBS + 0.1% TritonX-100 + 5% BSA for 1 h. The samples were incubated with the appropriate primary antibody at 4 °C overnight in a humidified chamber to prevent drying out. Next day, the samples were washed three times in 1× PBS + 0.1% TritonX-100 + 5% BSA before being incubated with the secondary antibody at 4 °C overnight. Finally, samples were washed three times in 1× PBS + 0.1% TritonX-100 and mounted in mounting medium.

To quantify the stained fibronectin signal between the control and the fibronectin morpholino-injected side, a straight line going from the notochord to the ventral side was drawn to divide the cryosection in half. The threshold was manually adjusted to detect and measure the fibronectin area between the two halves. Only sections with a relatively intact ectoderm were included in the analysis.

### MATLAB vector analysis

aVBI explants were labelled with H2B-mCherry for the ectodermal cells and AlexaFluor 488 fluorescence dextran for EM cells. Ectodermal nuclei and EM cells were segmented using Ilastik, and the resulting images were converted into binary. The binary images were imported into the PIVlab app (v3.06) in MATLAB (vR2024b 24.2.02740171) to generate vector direction plots. The angle between the velocity vectors of the cells and the coordinate system of the input image, which starts at the top left corner with the *x* axis (u-component) pointing right and the *y* axis (v-component) pointing down, was calculated and plotted from 180° to −180°. For a smooth and persistent migrating explant, we expected to see a more homogeneous and stable vector direction field, whereas for a more disordered migrating explant, we expected to see a more heterogeneous vector direction field. To quantify the degree of disorder, the colour maps of the vector field were converted into binary, and the area where the direction changed was quantified over time and converted to percentages.

### RT–qPCR

Total RNA purification from whole embryos was performed according to the manufacturer’s protocol (RNeasy Mini Kit, Qiagen, 74104). Complementary DNA was synthesized with random hexamers using a First Strand cDNA synthesis kit (ThermoScientific, K1621) according to the manufacturer’s protocol. Quantitative PCR (qPCR) was performed with the PowerUp SYBR Green master mix (Applied Biosystems, 25742). The 20-μl qPCR reaction was performed using a QuantStudio Real-Time PCR system as follows: hold stage: 2 min at 50 °C, 3 min at 95 °C. PCR stage 40 cycles: 20 s at 95 °C, 30 s at 57 °C, 30 s at 72 °C. Melt curve stage: 5 min at 72 °C, 15 s at 95 °C, 1 min at 60 °C, and 1 s at 95 °C.

### Cloning and in vitro transcription

The optogenetic constructs were first purchased from Addgene: SspB-mCherry-p63DH (176112) and Lck-mTurquoise2-iLID-SsrA (176125) before being subcloned into an empty pCS2+ vector using Gibson assembly (NEBuilder HiFi DNA Assembly Master Mix, E2621). pCS2+ vectors were broken into two pieces at the ampicillin-resistant gene to improve downstream screening. The resulting new plasmids were sequenced.

To in vitro transcribe, the plasmids were digested using NotI-HF (NEB, R3189S) and transcribed using an mMESSAGE mMACHINE SP6 transcription kit (Invitrogen, AM1340) according to the manufacturer’s protocol.

### Trajectory deviation, speed correlation and cosine similarity analyses

EM cells were manually tracked using the MTrackJ plugin, while the ectoderm was automatically tracked using TrackMate^133,134^ with StarDist^135^. For processing and analysis with Python, tracks were ingested into pandas data frames, and ectoderm track stubs (<10 time points) were removed. For ex vivo data, quality control excluded one outlier explant due to it being unhealthy. For in vivo data, where tracking was much harder, only samples where a sufficient number of EM cells (≥10) could be tracked for a sufficiently long time (≥20 frames) were included, resulting in *n* = 7 embryos out of 11 that were imaged across two separate experiments. Average local ectoderm velocity vectors were derived for the neighbourhood of each ectoderm cell and each EM cell by applying a Gaussian kernel (*σ* = 40 µm) to the surrounding ectodermal cell velocities, which favours the contribution of nearby ectodermal cells to the local average. Time-shift correlations (for trajectory deviations and for cell speeds) and cosine similarities measurements (for directional angles) between ectodermal cells and local ectoderm averages and between EM cells and local ectoderm averages were computed using the pandas corr method and scikit-learn’s paired_cosine_distances function, respectively. When calculating averages of these metrics over time, early time points (<80 ex vivo, <70 in vivo) and late time points (>180 ex vivo, >140 in vivo) were truncated away to preclude boundary effects. To calculate radial profiles from ex vivo data for comparison to model predictions, cluster centres were determined as the centroid of all ectoderm positions, rays from the centre to each cell were constructed, and all positions and vectors were rotated around the centre such that the centre-cell rays collapsed onto a single axis. All Python code to reproduce this analysis is available under the MIT open source software license on GitHub at https://github.com/WhoIsJack/ectoderm-myeloid-tracking-analysis.

### Active wetting model for ectoderm spreading

To capture ectoderm spreading, we extended the active wetting model for tissue spreading to include an influx of cells from the central aggregate into the ectoderm film (Fig. 4k). The active wetting model assumes that the tissue behaves as a liquid of viscosity *η* that spreads with velocity field **v**(*r*, *t*) on a substrate^64^. Spreading is driven by active traction forces generated by polarized cells at the tissue edge. These active tractions have magnitude *T*_a_ and are proportional to the tissue polarity field **p**(*r*, *t*). Force balance then imposes that

4 $${\rm\nabla }\cdot {\boldsymbol{\sigma }}=-{T}_{{\rm{a}}}{\bf{p}},$$

where

5 $$\sigma =\eta [{\rm\nabla }{\boldsymbol{v }}+{({\rm\nabla }{\boldsymbol{v }})}^{{\rm{T}}}]$$

is the viscous stress tensor. We assume that cells at the tissue edge polarize outwards, and the polarity then decays to zero towards the interior with a decay length *L*_c_. With these assumptions, the polarity field follows the following equation:

6 $${L}_{{\rm{c}}}^{2}{\nabla }^{2}{\bf{p}}={\bf{p}}.$$

In polar coordinates, and assuming that the polarity field is radial, ${\bf{p}}=p\left(r\right)\hat{{\bf{r}}}$, equation (6) becomes

7 $$\left[{r}^{2}\frac{{{\rm{d}}}^{2}}{{\rm{d}}{r}^{2}}+r\frac{{\rm{d}}}{{\rm{d}}r}-\left(1+\frac{{r}^{2}}{{L}_{{\rm{c}}}^{2}}\right)\right]p\left(r\right)=0.$$

Imposing the boundary condition *p*(*r* = *R*) = 1, where *R* is the tissue radius, the solution to equation (7) is

8 $$p\left(r\right)=\frac{{I}_{1}\left(r/{L}_{{\rm{c}}}\right)}{{I}_{1}\left(R/{L}_{{\rm{c}}}\right)},$$

where *I*_1_ is the modified Bessel function of the first kind and of first order.

With this solution, we can solve the force balance equation (4). For a radial flow field ${\bf{v}}=v\left(r\right)\hat{{\bf{r}}}$, and in polar coordinates, the non-vanishing components of the stress tensor are

9 $${\sigma }_{{rr}}=\eta {v }^{{\prime} }\left(r\right),\quad {\sigma }_{\theta \theta }=\eta \frac{v \left(r\right)}{r},$$

where *r* is the radial coordinate. In these polar components, the force balance reads

10 $${\sigma }_{{rr}}^{{\prime} }+\frac{{\sigma }_{{rr}}-{\sigma }_{\theta \theta }}{r}=-{T}_{a}p,$$

which gives

11 $$\left[\frac{{{\rm{d}}}^{2}}{{\rm{d}}{r}^{2}}+\frac{1}{r}\frac{{\rm{d}}}{{\rm{d}}r}-\frac{1}{{r}^{2}}\right]v \left(r\right)=-\frac{{T}_{a}}{\eta }p(r).$$

With the polarity field obtained above, the solution to equation (11) is

12 $$v \left(r\right)={Ar}+\frac{B}{r}+\frac{{T}_{{\rm{a}}}{L}_{{\rm{c}}}}{2\eta {I}_{1}\left(\frac{R}{{L}_{{\rm{c}}}}\right)}\left[r-{2L}_{{\rm{c}}}{I}_{1}\left(\frac{r}{{L}_{{\rm{c}}}}\right)\right]$$

for both the regions corresponding to the central aggregate (*r* ≤ *R*_a_) and the peripheral spreading film (*R*_a_ ≤ *r* ≤ *R*), as shown in Fig. 4k. Here, *A* and *B* are integration constants, which are different for each region.

We now impose boundary conditions to determine the integration constants. In the central region, we set *B* = 0 to avoid a divergence at the origin. At the peripheral region, indicated by the superscript, we impose a stress-free boundary condition at the tissue edge:

13 $${\sigma }_{{rr}}^{{\rm{per}}}\left(R\right)=0.$$

Moreover, we impose that the velocity is continuous across the two regions:

14 $${v}_{{\rm{cen}}}\left({R}_{{\rm{a}}}\right)={v }_{{\rm{per}}}\left({R}_{{\rm{a}}}\right),$$

where the superscripts indicate the central and peripheral regions, respectively. Finally, to account for the influx of cells from the aggregate, we impose that the flux into the peripheral region that would exist due just to migration is supplemented by an additional influx *J*:

15 $$\nabla \cdot {\boldsymbol{v}}_{\mathrm{per}}{|}_{r={R}_{{\rm{a}}}}=\nabla \cdot {\boldsymbol{v} }_{\mathrm{mig}}{|}_{r={R}_{{\rm{a}}}}+J.$$

Here, ${v }_{{\rm{mig}}}$ is the flow field of the tissue in the absence of influx, which is the solution to the original active wetting model, given by

16 $${v }_{\mathrm{mig}}\left(r\right)=\frac{{T}_{{\rm{a}}}{L}_{{\rm{c}}}}{2\eta {I}_{1}\left(\frac{R}{{L}_{{\rm{c}}}}\right)}\left\{\left.\left[{I}_{0}\left(\frac{R}{{L}_{{\rm{c}}}}\right)+{I}_{2}\left(\frac{R}{{L}_{{\rm{c}}}}\right)\right]r-2{L}_{{\rm{c}}}{I}_{1}\left(\frac{r}{{L}_{{\rm{c}}}}\right)\right\}\right.$$

Solving equations (13) to (15) to obtain the integration constants *A*_cen_, *A*_per_ and *B*_per_, we arrive at the solution

17 $${v }_{{\rm{cen}}}\left(r\right)=\frac{J}{2}\left(1+\frac{{R}^{2}}{{R}_{{\rm{a}}}^{2}}\right)r+\frac{{T}_{{\rm{a}}}{L}_{{\rm{c}}}}{\eta }\left\{\left.\left[\frac{{I}_{0}\left(\frac{R}{{L}_{{\rm{c}}}}\right)}{{I}_{1}\left(\frac{R}{{L}_{{\rm{c}}}}\right)}-\frac{{L}_{{\rm{c}}}}{R}\right]r-{L}_{{\rm{c}}}\frac{{I}_{1}\left(\frac{r}{{L}_{{\rm{c}}}}\right)}{{I}_{1}\left(\frac{R}{{L}_{{\rm{c}}}}\right)}\right\}\right.,$$

18 $${v }_{{\rm{per}}}\left(r\right)=\frac{J}{2}\left(1+\frac{{R}^{2}}{{r}^{2}}\right)r+\frac{{T}_{{\rm{a}}}{L}_{{\rm{c}}}}{\eta }\left\{\left.\left[\frac{{I}_{0}\left(\frac{R}{{L}_{{\rm{c}}}}\right)}{{I}_{1}\left(\frac{R}{{L}_{{\rm{c}}}}\right)}-\frac{{L}_{{\rm{c}}}}{R}\right]r-{L}_{{\rm{c}}}\frac{{I}_{1}\left(\frac{r}{{L}_{{\rm{c}}}}\right)}{{I}_{1}\left(\frac{R}{{L}_{{\rm{c}}}}\right)}\right\}\right.,$$

which produces the theory results plotted in Fig. 4l(i). We also introduced this result in equation (3) to predict the EM-cell flow, plotted in Fig. 4l(ii).

The parameter values used to construct the plots are as follows: *η* = 50 MPa s, *T*_a_=15 Pa, *L*_c_ = 25 µm, *R* = [541, 632, 724, 815] µm (Fig. 4l(i)), *R* = 800 µm (Fig. 4l(ii)), *R*_a_ = 250 µm, *J* = 0.00576 h^−1^, *v*_s_ = 0.01 µm min^−1^ and *v*_max_ = 0.85 µm min^−1^.

### Statistical analysis

All graphs were plotted using the ggplot2 R package or Matplotlib from Python. Two-tailed unpaired *t*-tests were used to compare between two groups. ANOVA with Tukey’s multiple comparison was used to compare between three or more conditions. The *n* (number of cells analysed) and *N* (number of embryos) values are shown in each figure legend. We anticipated that the effect size of the observed phenotype would be in the range of small to medium at the explant level based on preliminary data. We calculated the hypothetical number of explants (or embryos) needed to detect an effect size of 0.3 (medium) at a significance level (alpha) of 0.05 and a statistical power of 0.8 to be 175 explants (or embryos). This was impractical to perform. Therefore, to reduce the use of animals in experimentation, as well as to capture the full spectrum of variables between individual cells, we decided to use the *n* number for statistical analyses (unless specified otherwise). To reduce the chance of a false positive and ensure the consistency and reproducibility of the observed effects, we drew our conclusions from experiments that were performed in multiple different contexts and experimental set-ups, as well as from embryos collected from at least three different female frogs on separate days. Analyses were carried out blind whenever possible, with embryos or explants being represented as numbers while being analysed, and embryos randomly mixed from different frogs to ensure maximal variations were captured. Nevertheless, treatments with morpholinos often result in changes in their morphology so blind analyses may not always be possible. The data distribution was assumed to be normal, but this was not formally tested. Statistical tests were chosen following guidance from the *Handbook of Biological Statistics*^136^.

### Reporting summary

Further information on research design is available in the Nature Portfolio Reporting Summary linked to this Article.

## Online content

Any methods, additional references, Nature Portfolio reporting summaries, source data, extended data, supplementary information, acknowledgements, peer review information; details of author contributions and competing interests; and statements of data and code availability are available at https://doi.org/10.1038/s41556-026-02058-9.

## Supplementary information

Reporting Summary

Peer Review File

Supplementary Table 1 Reagents.

Supplementary Table 2 Light microscopy reporting table.

Supplementary Video 1 Video.

Supplementary Video 2 Video.

Supplementary Video 3 Video.

Supplementary Video 4 Video.

Supplementary Video 5 Video.

Supplementary Video 6 Video.

Supplementary Video 7 Video.

Supplementary Video 8 Video.

Supplementary Video 9 Video.

Supplementary Video 10 Video.

Supplementary Video 11 Video.

Supplementary Video 12 Video.

Supplementary Video 13 Video.

Supplementary Video 14 Video.

Supplementary Video 15 Video.

Supplementary Video 16 Video.

Supplementary Video 17 Video.

Supplementary Video 18 Video.

Supplementary Video 19 Video.

Supplementary Video 20 Video.

Supplementary Video 21 Video.

Supplementary Video 22 Video.

Supplementary Video 23 Video.

Supplementary Video 24 Video.

Supplementary Video 25 Video.

Supplementary Video 26 Video.

Supplementary Video 27 Video.

Supplementary Video 28 Video.

Supplementary Video 29 Video.

Supplementary Video 30 Video.

Supplementary Video 31 Video.

## Source data

Source Data Figures Statistical source data for all figures and Extended Data except for Figure 7Fi (due to size).

Source Data Fig. 7 Statistical source data for Figure 7Fi.

## Data Availability

All data are available in the main text or the supplementary materials. Requests to access the data and/or methodologies can be sent to the corresponding authors. Source data are provided with this paper.

## Code availability

The codes to solve the model equations and to fit them to the experimental data are provided in the materials and methods. The codes to analyse the correlation analysis and cosine similarity are available on GitHub at https://github.com/WhoIsJack/ectoderm-myeloid-tracking-analysis.

## References

[1] Doyle, A. D., Carvajal, N., Jin, A., Matsumoto, K. & Yamada, K. M. Local 3D matrix microenvironment regulates cell migration through spatiotemporal dynamics of contractility-dependent adhesions. *Nat. Commun.* **6**, 8720 (2015). 10.1038/ncomms9720 PMC4643399 26548801

[2] Bajanca, F. et al. In vivo topology converts competition for cell-matrix adhesion into directional migration. *Nat. Commun.* **10**, 1518 (2019). 10.1038/s41467-019-09548-5 PMC6447549 30944331

[3] Yamada, K. M., Doyle, A. D. & Lu, J. Cell-3D matrix interactions: recent advances and opportunities. *Trends Cell Biol.* **32**, 883–895 (2022). 10.1016/j.tcb.2022.03.002 PMC9464680 35410820

[4] Saraswathibhatla, A., Indana, D. & Chaudhuri, O. Cell–extracellular matrix mechanotransduction in 3D. *Nat. Rev. Mol. Cell. Biol.* **24**, 495–516, 10.1038/s41580-023-00583-1 (2023). PMC10656994 36849594

[5] Sleeboom, J. J. F. et al. The extracellular matrix as hallmark of cancer and metastasis: from biomechanics to therapeutic targets. *Sci. Transl. Med.* **16**, eadg3840 (2024). 10.1126/scitranslmed.adg3840 38170791

[6] Chitty, J. L. & Cox, T. R. The extracellular matrix in cancer: from understanding to targeting. *Trends Cancer* **11**, 839–849 (2025). 10.1016/j.trecan.2025.05.003 40467384

[7] Chepizhko, O. et al. Confined cell migration along extracellular matrix space in vivo. *Proc. Natl Acad. Sci. USA* **122**, e2414009121 (2025). 10.1073/pnas.2414009121 PMC11725923 39793073

[8] Cai, D. et al. Mechanical feedback through E-cadherin promotes direction sensing during collective cell migration. *Cell* **157**, 1146–1159 (2014). 10.1016/j.cell.2014.03.045 PMC4118667 24855950

[9] Dai, W. et al. Tissue topography steers migrating *Drosophila* border cells. *Science* **370**, 987–990 (2020). 10.1126/science.aaz4741 PMC8103818 33214282

[10] Knutsdottir, H. et al. Polarization and migration in the zebrafish posterior lateral line system. *PLoS Comput. Biol.* **13**, e1005451 (2017). 10.1371/journal.pcbi.1005451 PMC5393887 28369079

[11] Dalle Nogare, D. E., Natesh, N., Vishwasrao, H. D., Shroff, H. & Chitnis, A. B. Zebrafish Posterior Lateral Line primordium migration requires interactions between a superficial sheath of motile cells and the skin. *Elife* **9**, e58251 (2020). 10.7554/eLife.58251 PMC7688310 33237853

[12] Karaiwa, A. et al. Relationship between surrounding tissue morphology and directional collective migration of the posterior lateral line primordium in zebrafish. *Genes Cells* **25**, 582–592 (2020). 10.1111/gtc.12793 32516841

[13] Le, H. A. & Mayor, R. Neighboring cells as living substrates for guiding collective cell migration during development. *Cold Spring Harb. Perspect. Biol.* a041741 10.1101/cshperspect.a041741 (2025). PMC12951764 40389309

[14] Barriga, E. H., Franze, K., Charras, G. & Mayor, R. Tissue stiffening coordinates morphogenesis by triggering collective cell migration in vivo. *Nature* **554**, 523–527 (2018). 10.1038/nature25742 PMC6013044 29443958

[15] Shellard, A. & Mayor, R. Collective durotaxis along a self-generated stiffness gradient in vivo. *Nature* **600**, 690–694 (2021). 10.1038/s41586-021-04210-x 34880503

[16] Le, H. A. & Mayor, R. Cell–matrix and cell–cell interaction mechanics in guiding migration. *Biochem. Soc. Trans.* **51**, 1733–1745 (2023). 10.1042/BST20230211 PMC10586762 37610008

[17] Herbomel, P., Thisse, B. & Thisse, C. Ontogeny and behaviour of early macrophages in the zebrafish embryo. *Development* **126**, 3735–3745 (1999). 10.1242/dev.126.17.3735 10433904

[18] Perçin, G. et al. Embryonic macrophages orchestrate niche cell homeostasis for the establishment of the definitive hematopoietic stem cell pool. *Nat. Commun.* **16**, 4428 (2025). 10.1038/s41467-025-59059-9 PMC12078706 40368907

[19] Smith, S. J. & Mohun, T. J. Early cardiac morphogenesis defects caused by loss of embryonic macrophage function in *Xenopus*. *Mech. Dev.* **128**, 303–315 (2011). 10.1016/j.mod.2011.04.002 PMC3157588 21515365

[20] Leid, J. et al. Primitive embryonic macrophages are required for coronary development and maturation. *Circ. Res.* **118**, 1498–1511 (2016). 10.1161/CIRCRESAHA.115.308270 PMC5567774 27009605

[21] Cahill, T. J. et al. Tissue-resident macrophages regulate lymphatic vessel growth and patterning in the developing heart. *Development* **148**, dev194563 (2021). 10.1242/dev.194563 PMC7875498 33462113

[22] Ferrero, G. et al. Embryonic microglia derive from primitive macrophages and are replaced by *cmyb*-dependent definitive microglia in zebrafish. *Cell Rep.* **24**, 130–141 (2018). 10.1016/j.celrep.2018.05.066 29972775

[23] Silvin, A., Qian, J. & Ginhoux, F. Brain macrophage development, diversity and dysregulation in health and disease. *Cell. Mol. Immunol.* **20**, 1277–1289 (2023). 10.1038/s41423-023-01053-6 PMC10616292 37365324

[24] Ciau-Uitz, A., Walmsley, M. & Patient, R. Distinct origins of adult and embryonic blood in *Xenopus*. *Cell* **102**, 787–796 (2000). 10.1016/s0092-8674(00)00067-2 11030622

[25] Costa, R. M. B., Soto, X., Chen, Y., Zorn, A. M. & Amaya, E. *spib* is required for primitive myeloid development in *Xenopus*. *Blood* **112**, 2287–2296 (2008). 10.1182/blood-2008-04-150268 PMC2577559 18594023

[26] Agricola, Z. N. et al. Identification of genes expressed in the migrating primitive myeloid lineage of *Xenopus laevis*. *Dev. Dyn.* **245**, 47–55 (2016). 10.1002/dvdy.24314 PMC5387764 26264370

[27] Theveneau, E. & Mayor, R. Neural crest delamination and migration: from epithelium-to-mesenchyme transition to collective cell migration. *Dev. Biol.* **366**, 34–54 (2012). 10.1016/j.ydbio.2011.12.041 22261150

[28] Mayor, R. & Theveneau, E. The neural crest. *Development* **140**, 2247–2251 (2013). 10.1242/dev.091751 23674598

[29] Szabó, A. & Mayor, R. Mechanisms of neural crest migration. *Annu. Rev. Genet.* **52**, 43–63 (2018). 10.1146/annurev-genet-120417-031559 30476447

[30] Muinonen-Martin, A. J. et al. Melanoma cells break down LPA to establish local gradients that drive chemotactic dispersal. *PLoS Biol.* **12**, e1001966 (2014). 10.1371/journal.pbio.1001966 PMC4196730 25313567

[31] Juin, A. et al. N-WASP control of LPAR1 trafficking establishes response to self-generated LPA gradients to promote pancreatic cancer cell metastasis. *Dev. Cell* **51**, 431–445.e7 (2019). 10.1016/j.devcel.2019.09.018 PMC6863394 31668663

[32] Al-Hilal, T. A. et al. Durotaxis is a driver and potential therapeutic target in lung fibrosis and metastatic pancreatic cancer. *Nat. Cell Biol.* **27**, 1543–1554 (2025). 10.1038/s41556-025-01697-8 PMC12431851 40925952

[33] Heasman, J., Hynes, R. O., Swan, A. P., Thomas, V. & Wylie, C. C. Primordial germ cells of *Xenopus* embryos: the role of fibronectin in their adhesion during migration. *Cell* **27**, 437–447 (1981). 10.1016/0092-8674(81)90385-8 6101198

[34] Lee, G., Hynes, R. & Kirschner, M. Temporal and spatial regulation of fibronectin in early *Xenopus* development. *Cell* **36**, 729–740 (1984). 10.1016/0092-8674(84)90353-2 6697394

[35] Davidson, L. A., Marsden, M., Keller, R. & Desimone, D. W. Integrin α5β1 and fibronectin regulate polarized cell protrusions required for *Xenopus* convergence and extension. *Curr. Biol.* **16**, 833–844 (2006). 10.1016/j.cub.2006.03.038 16682346

[36] Moody, S. A. Fates of the blastomeres of the 32-cell-stage *Xenopus* embryo. *Dev. Biol.* **122**, 300–319 (1987). 10.1016/0012-1606(87)90296-x 3596014

[37] Costa, R., Chen, Y., Paredes, R. & Amaya, E. Labeling primitive myeloid progenitor cells in *Xenopus*. In *Progenitor Cells: Methods and Protocols* (eds Mace, K. A. & Braun, K. M.) 141–155 10.1007/978-1-61779-980-8_11 (Humana Press, 2012). 22914938

[38] Zahn, N. et al. Normal table of *Xenopus* development: a new graphical resource. *Development* **149**, dev200356 (2022). 10.1242/dev.200356 PMC9445888 35833709

[39] Millius, A. & Weiner, O. D. Manipulation of neutrophil-like HL-60 cells for the study of directed cell migration. *Methods Mol. Biol.* **591**, 147–158 (2010). 10.1007/978-1-60761-404-3_9 PMC3128798 19957129

[40] Khachaturyan, G. et al. Temperature-sensitive migration dynamics in neutrophil-differentiated HL-60 cells. *Sci. Rep.* **12**, 7053 (2022). 10.1038/s41598-022-10858-w PMC9054779 35488042

[41] Kandow, C. E., Georges, P. C., Janmey, P. A. & Beningo, K. A. Polyacrylamide hydrogels for cell mechanics: steps toward optimization and alternative uses. *Methods Cell. Biol.* **83**, 29–46 (2007). 10.1016/S0091-679X(07)83002-0 17613303

[42] Worthen, C. A., Rittié, L. & Fisher, G. J. Mechanical deformation of cultured cells with hydrogels. *Methods Mol. Biol.* **1627**, 245–251 (2017). 10.1007/978-1-4939-7113-8_17 PMC5854178 28836207

[43] Liu, Y.-J. et al. Confinement and low adhesion induce fast amoeboid migration of slow mesenchymal cells. *Cell* **160**, 659–672 (2015). 10.1016/j.cell.2015.01.007 25679760

[44] Lomakin, A. J. et al. The nucleus acts as a ruler tailoring cell responses to spatial constraints. *Science* **370**, eaba2894 (2020). 10.1126/science.aba2894 PMC8059074 33060332

[45] Venturini, V. et al. The nucleus measures shape changes for cellular proprioception to control dynamic cell behavior. *Science* **370**, eaba2644 (2020). 10.1126/science.aba2644 33060331

[46] García-Arcos, J. M., Jha, A., Waterman, C. M. & Piel, M. Blebology: principles of bleb-based migration. *Trends Cell Biol.* **34**, 838–853 (2024). 10.1016/j.tcb.2024.02.009 PMC11424778 38538441

[47] Jha, A., Chandra, A. & Farahani, P.E. et al. CD44 restricts EGFR mobility to polarize cytoskeletal signalling modules driving bleb-based migration. *Nat. Cell Biol.* **28**, 1408–1423 (2026). 10.1038/s41556-026-01981-1 PMC13364714 42410113

[48] Petrie, R. J., Koo, H. & Yamada, K. M. Generation of compartmentalized pressure by a nuclear piston governs cell motility in a 3D matrix. *Science* **345**, 1062–1065 (2014). 10.1126/science.1256965 PMC5248932 25170155

[49] Petrie, R. J., Harlin, H. M., Korsak, L. I. T. & Yamada, K. M. Activating the nuclear piston mechanism of 3D migration in tumor cells. *J. Cell Biol.* **216**, 93–100 (2016). 10.1083/jcb.201605097 PMC5223602 27998990

[50] Renkawitz, J. et al. Nuclear positioning facilitates amoeboid migration along the path of least resistance. *Nature* **568**, 546–550 (2019). 10.1038/s41586-019-1087-5 PMC7217284 30944468

[51] Graziani, V., Rodriguez-Hernandez, I., Maiques, O. & Sanz-Moreno, V. The amoeboid state as part of the epithelial-to-mesenchymal transition programme. *Trends Cell Biol.* **32**, 228–242 (2022). 10.1016/j.tcb.2021.10.004 34836782

[52] Herbomel, P., Thisse, B. & Thisse, C. Zebrafish early macrophages colonize cephalic mesenchyme and developing brain, retina and epidermis through a M-CSF receptor-dependent invasive process. *Dev. Biol.* **238**, 274–288 (2001). 10.1006/dbio.2001.0393 11784010

[53] Toh, M.-L. et al. Bone- and cartilage-protective effects of a monoclonal antibody against colony-stimulating factor 1 receptor in experimental arthritis. *Arthritis Rheumatol.* **66**, 2989–3000 (2014). 10.1002/art.38624 24623505

[54] Gilboa, D. et al. Erythropoietin enhances Kupffer cell number and activity in the challenged liver. *Sci. Rep.* **7**, 10379 (2017). 10.1038/s41598-017-11082-7 PMC5583293 28871174

[55] Weavers, H. et al. Systems analysis of the dynamic inflammatory response to tissue damage reveals spatiotemporal properties of the wound attractant gradient. *Curr. Biol.* **26**, 1975–1989 (2016). 10.1016/j.cub.2016.06.012 PMC4985561 27426513

[56] Zechini, L. et al. *Drosophila* complement-like Mcr acts as a wound-induced inflammatory chemoattractant. *Curr. Biol.* **35**, 1656–1664.e4 (2025). 10.1016/j.cub.2025.02.036 PMC12254109 40107264

[57] Gómez-de-Mariscal, E. et al. CellTracksColab is a platform that enables compilation, analysis and exploration of cell tracking data. *PLoS Biol.* **22**, e3002740 (2024). 10.1371/journal.pbio.3002740 PMC11335138 39116189

[58] von Chamier, L. et al. Democratising deep learning for microscopy with ZeroCostDL4Mic. *Nat. Commun.* **12**, 2276 (2021). 10.1038/s41467-021-22518-0 PMC8050272 33859193

[59] Lämmermann, T. & Kastenmüller, W. Concepts of GPCR-controlled navigation in the immune system. *Immunol. Rev.* **289**, 205–231 (2019). 10.1111/imr.12752 PMC6487968 30977203

[60] Floris, S., Van Den Born, J., Van Der Pol, S. M. A., Dijkstra, C. D. & De Vries, H. E. Heparan sulfate proteoglycans modulate monocyte migration across cerebral endothelium. *J. Neuropathol. Exp. Neurol.* **62**, 780–790 (2003). 10.1093/jnen/62.7.780 12901703

[61] Schumann, K. et al. Immobilized chemokine fields and soluble chemokine gradients cooperatively shape migration patterns of dendritic cells. *Immunity* **32**, 703–713 (2010). 10.1016/j.immuni.2010.04.017 20471289

[62] Alotaibi, F. S. et al. Interactions of proteins with heparan sulfate. *Essays Biochem.* **68**, 479–489 (2024). 10.1042/EBC20230093 PMC11625861 38646914

[63] Priestley, M. J. et al. Leukocytes have a heparan sulfate glycocalyx that regulates recruitment during psoriasis-like skin inflammation. *Sci. Signal.* **18**, eadr0011 (2025). 10.1126/scisignal.adr0011 41187220

[64] Pérez-González, C. et al. Active wetting of epithelial tissues. *Nat. Phys.* **15**, 79–88 (2019). 10.1038/s41567-018-0279-5 PMC6753015 31537984

[65] Berg, S. et al. ilastik: interactive machine learning for (bio)image analysis. *Nat. Methods* **16**, 1226–1232 (2019). 10.1038/s41592-019-0582-9 31570887

[66] Thielicke, W. & Stamhuis, E. J. PIVlab – towards user-friendly, affordable and accurate digital particle image velocimetry in MATLAB. *J. Open Res. Softw.* **2**, e30 (2014).

[67] Thielicke, W. & Sonntag, R. Particle image velocimetry for MATLAB: accuracy and enhanced algorithms in PIVlab. *J. Open Res. Softw.* **9**, 12 (2021).

[68] Skoglund, P. & Keller, R. *Xenopus* fibrillin regulates directed convergence and extension. *Dev. Biol.* **301**, 404–416 (2007). 10.1016/j.ydbio.2006.09.005 PMC2637453 17027959

[69] Lämmermann, T. et al. Rapid leukocyte migration by integrin-independent flowing and squeezing. *Nature* **453**, 51–55 (2008). 10.1038/nature06887 18451854

[70] Reversat, A. et al. Cellular locomotion using environmental topography. *Nature* **582**, 582–585 (2020). 10.1038/s41586-020-2283-z 32581372

[71] Davidson, L. A., Keller, R. & DeSimone, D. W. Assembly and remodeling of the fibrillar fibronectin extracellular matrix during gastrulation and neurulation in *Xenopus laevis*. *Dev. Dyn.* **231**, 888–895 (2004). 10.1002/dvdy.20217 15517579

[72] Alert, R. & Trepat, X. Physical models of collective cell migration. *Annu. Rev. Condens. Matter Phys.* **11**, 77–101 (2020).

[73] Beaune, G. et al. How cells flow in the spreading of cellular aggregates. *Proc. Natl Acad. Sci. USA* **111**, 8055–8060 (2014). 10.1073/pnas.1323788111 PMC4050549 24835175

[74] Staddon, M. F., Murrell, M. P. & Banerjee, S. Interplay between substrate rigidity and tissue fluidity regulates cell monolayer spreading. *Soft Matter* **18**, 7877–7886 (2022). 10.1039/d2sm00757f PMC9700261 36205535

[75] Yousafzai, M. S. et al. Cell-matrix elastocapillary interactions drive pressure-based wetting of cell aggregates. *Phys. Rev. X* **12**, 031027 (2022). 10.1103/physrevx.12.031027 PMC10673637 38009085

[76] Douezan, S. et al. Spreading dynamics and wetting transition of cellular aggregates. *Proc. Natl Acad. Sci. USA* **108**, 7315–7320 (2011). 10.1073/pnas.1018057108 PMC3088609 21504944

[77] Douezan, S., Dumond, J. & Brochard-Wyart, F. Wetting transitions of cellular aggregates induced by substrate rigidity. *Soft Matter* **8**, 4578–4583 (2012).

[78] Beaune, G. et al. Reentrant wetting transition in the spreading of cellular aggregates. *Soft Matter* **13**, 8474–8482 (2017). 10.1039/c7sm00768j 29091088

[79] Alert, R. & Casademunt, J. Role of substrate stiffness in tissue spreading: wetting transition and tissue durotaxis. *Langmuir* **35**, 7571–7577 (2019). 10.1021/acs.langmuir.8b02037 30281318

[80] Pallarès, M. E. et al. Stiffness-dependent active wetting enables optimal collective cell durotaxis. *Nat. Phys.* **19**, 279–289 (2023).

[81] Levskaya, A., Weiner, O. D., Lim, W. A. & Voigt, C. A. Spatiotemporal control of cell signalling using a light-switchable protein interaction. *Nature* **461**, 997–1001 (2009). 10.1038/nature08446 PMC2989900 19749742

[82] Guntas, G. et al. Engineering an improved light-induced dimer (iLID) for controlling the localization and activity of signaling proteins. *Proc. Natl Acad. Sci. USA* **112**, 112–117 (2015). 10.1073/pnas.1417910112 PMC4291625 25535392

[83] Mahlandt, E. K. et al. Opto-RhoGEFs, an optimized optogenetic toolbox to reversibly control Rho GTPase activity on a global to subcellular scale, enabling precise control over vascular endothelial barrier strength. *eLife* **12**, RP84364 (2023). 10.7554/eLife.84364 PMC10393062 37449837

[84] Barry, D. J., Durkin, C. H., Abella, J. V. & Way, M. Open source software for quantification of cell migration, protrusions and fluorescence intensities. *J. Cell Biol.* **209**, 163–180 (2015). 10.1083/jcb.201501081 PMC4395480 25847537

[85] Arzt, M. et al. LABKIT: labeling and segmentation toolkit for big image data. *Front. Comput. Sci.* **4**, 777728 (2022).

[86] Wallingford, J. B., Fraser, S. E. & Harland, R. M. Convergent extension: the molecular control of polarized cell movement during embryonic development. *Dev. Cell* **2**, 695–706 (2002). 10.1016/s1534-5807(02)00197-1 12062082

[87] Stubbs, J. L., Davidson, L., Keller, R. & Kintner, C. Radial intercalation of ciliated cells during *Xenopus* skin development. *Development* **133**, 2507–2515 (2006). 10.1242/dev.02417 16728476

[88] Tada, M. & Heisenberg, C.-P. Convergent extension: using collective cell migration and cell intercalation to shape embryos. *Development* **139**, 3897–3904 (2012). 10.1242/dev.073007 23048180

[89] Walck-Shannon, E. & Hardin, J. Cell intercalation from top to bottom. *Nat. Rev. Mol. Cell Biol.* **15**, 34–48 (2014). 10.1038/nrm3723 PMC4550482 24355988

[90] Huebner, R. J. & Wallingford, J. B. Coming to consensus: a unifying model emerges for convergent extension. *Dev. Cell* **46**, 389–396 (2018). 10.1016/j.devcel.2018.08.003 PMC6140352 30130529

[91] Sutherland, A., Keller, R. & Lesko, A. Convergent extension in mammalian morphogenesis. *Semin. Cell Dev. Biol.* **100**, 199–211 (2020). 10.1016/j.semcdb.2019.11.002 PMC7071967 31734039

[92] Behrndt, M. et al. Forces driving epithelial spreading in zebrafish gastrulation. *Science* **338**, 257–260 (2012). 10.1126/science.1224143 23066079

[93] Szabó, A. et al. The molecular basis of radial intercalation during tissue spreading in early development. *Dev. Cell* **37**, 213–225 (2016). 10.1016/j.devcel.2016.04.008 PMC4865533 27165554

[94] Wallmeyer, B., Trinschek, S., Yigit, S., Thiele, U. & Betz, T. Collective cell migration in embryogenesis follows the laws of wetting. *Biophys. J.* **114**, 213–222 (2018). 10.1016/j.bpj.2017.11.011 PMC5773767 29320689

[95] Sokol, S. Y. Analysis of dishevelled signalling pathways during *Xenopus* development. *Curr. Biol.* **6**, 1456–1467 (1996). 10.1016/s0960-9822(96)00750-6 8939601

[96] Kazanskaya, O. et al. R-Spondin2 is a secreted activator of Wnt/β-catenin signaling and is required for *Xenopus* myogenesis. *Dev. Cell* **7**, 525–534 (2004). 10.1016/j.devcel.2004.07.019 15469841

[97] Fonar, Y. et al. Focal adhesion kinase protein regulates Wnt3a gene expression to control cell fate specification in the developing neural plate. *Mol. Biol. Cell* **22**, 2409–2421 (2011). 10.1091/mbc.E10-12-0932 PMC3128541 21551070

[98] Bjerke, M. A., Dzamba, B. J., Wang, C. & DeSimone, D. W. FAK is required for tension-dependent organization of collective cell movements in *Xenopus* mesendoderm. *Dev. Biol.* **394**, 340–356 (2014). 10.1016/j.ydbio.2014.07.023 PMC4172504 25127991

[99] Rana, A. A., Collart, C., Gilchrist, M. J. & Smith, J. C. Defining synphenotype groups in *Xenopus tropicalis* by use of antisense morpholino oligonucleotides. *PLoS Genet.* **2**, e193 (2006). 10.1371/journal.pgen.0020193 PMC1636699 17112317

[100] Petridou, N. I., Stylianou, P. & Skourides, P. A. A dominant-negative provides new insights into FAK regulation and function in early embryonic morphogenesis. *Development* **140**, 4266–4276 (2013). 10.1242/dev.096073 24048589

[101] Scarpa, E. et al. Cadherin switch during EMT in neural crest cells leads to contact inhibition of locomotion via repolarization of forces. *Dev. Cell* **34**, 421–434 (2015). 10.1016/j.devcel.2015.06.012 PMC4552721 26235046

[102] Davis, J. R. et al. Inter-cellular forces orchestrate contact inhibition of locomotion. *Cell* **161**, 361–373 (2015). 10.1016/j.cell.2015.02.015 PMC4398973 25799385

[103] Lämmermann, T. et al. Neutrophil swarms require LTB4 and integrins at sites of cell death in vivo. *Nature* **498**, 371–375 (2013). 10.1038/nature12175 PMC3879961 23708969

[104] Tweedy, L. et al. Seeing around corners: cells solve mazes and respond at a distance using attractant breakdown. *Science* **369**, eaay9792 (2020). 10.1126/science.aay9792 32855311

[105] Alanko, J. et al. CCR7 acts as both a sensor and a sink for CCL19 to coordinate collective leukocyte migration. *Sci. Immunol.* **8**, eadc9584 (2023). 10.1126/sciimmunol.adc9584 37656776

[106] Uçar, M. C., Alsberga, Z., Alanko, J., Sixt, M. & Hannezo, E. Self-generated chemotaxis of mixed cell populations. *Proc. Natl Acad. Sci. USA* **122**, e2504064122 (2025). 10.1073/pnas.2504064122 PMC12403085 40838890

[107] Häcker, U., Nybakken, K. & Perrimon, N. Heparan sulphate proteoglycans: the sweet side of development. *Nat. Rev. Mol. Cell Biol.* **6**, 530–541 (2005). 10.1038/nrm1681 16072037

[108] Rabelink, T. J. et al. Heparanase: roles in cell survival, extracellular matrix remodelling and the development of kidney disease. *Nat. Rev. Nephrol.* **13**, 201–212 (2017). 10.1038/nrneph.2017.6 28163306

[109] Corti, F. et al. N-terminal syndecan-2 domain selectively enhances 6-O heparan sulfate chains sulfation and promotes VEGFA165-dependent neovascularization. *Nat. Commun.* **10**, 1562 (2019). 10.1038/s41467-019-09605-z PMC6450910 30952866

[110] Venero Galanternik, M., Kramer, K. L. & Piotrowski, T. Heparan sulfate proteoglycans regulate Fgf signaling and cell polarity during collective cell migration. *Cell Rep.* **10**, 414–428 (2015). 10.1016/j.celrep.2014.12.043 PMC4531098 25600875

[111] Sarris, M. et al. Inflammatory chemokines direct and restrict leukocyte migration within live tissues as glycan-bound gradients. *Curr. Biol.* **22**, 2375–2382 (2012). 10.1016/j.cub.2012.11.018 23219724

[112] Weber, M. et al. Interstitial dendritic cell guidance by haptotactic chemokine gradients. *Science* **339**, 328–332 (2013). 10.1126/science.1228456 23329049

[113] Bergert, M., Chandradoss, S. D., Desai, R. A. & Paluch, E. Cell mechanics control rapid transitions between blebs and lamellipodia during migration. *Proc. Natl Acad. Sci. USA* **109**, 14434–14439 (2012). 10.1073/pnas.1207968109 PMC3437886 22786929

[114] Petrie, R. J., Gavara, N., Chadwick, R. S. & Yamada, K. M. Nonpolarized signaling reveals two distinct modes of 3D cell migration. *J. Cell Biol.* **197**, 439–455 (2012). 10.1083/jcb.201201124 PMC3341168 22547408

[115] Sixt, M. Cell migration: fibroblasts find a new way to get ahead. *J. Cell Biol.* **197**, 347–349 (2012). 10.1083/jcb.201204039 PMC3341167 22547405

[116] Kumar, N., Soni, H., Ramaswamy, S. & Sood, A. K. Flocking at a distance in active granular matter. *Nat. Commun.* **5**, 4688 (2014). 10.1038/ncomms5688 25181961

[117] Doostmohammadi, A. & Ladoux, B. Physics of liquid crystals in cell biology. *Trends Cell Biol.* **32**, 140–150 (2022). 10.1016/j.tcb.2021.09.012 34756501

[118] Smutny, M. et al. Friction forces position the neural anlage. *Nat. Cell Biol.* **19**, 306–317 (2017). 10.1038/ncb3492 PMC5635970 28346437

[119] Shellard, A. et al. Frictiotaxis underlies focal adhesion-independent durotaxis. *Nat. Commun.* **16**, 3811 (2025). 10.1038/s41467-025-58912-1 PMC12019219 40268931

[120] Maiuri, P. et al. Actin flows mediate a universal coupling between cell speed and cell persistence. *Cell* **161**, 374–386 (2015). 10.1016/j.cell.2015.01.056 25799384

[121] Chang, C.-T., Tsai, T. & Solnica-Krezel, L. Temporal regulation of endoderm convergence and extension by the BMP activity gradient through mesoderm-dependent and independent mechanisms. *Cells Dev.* **184**, 204021 (2025). 10.1016/j.cdev.2025.204021 PMC13576405 40090551

[122] Pakshir, P. et al. Dynamic fibroblast contractions attract remote macrophages in fibrillar collagen matrix. *Nat. Commun.* **10**, 1850 (2019). 10.1038/s41467-019-09709-6 PMC6478854 31015429

[123] Chu, C.-W. et al. Mechanical cues polarize ADIP protein complexes to control vertebrate morphogenesis and wound healing. *Curr. Biol.* **35**, 3315–3326.e4 (2025). 10.1016/j.cub.2025.05.069 PMC12321261 40562038

[124] Barlan, K., Cetera, M. & Horne-Badovinac, S. Fat2 and Lar define a basally localized planar signaling system controlling collective cell migration. *Dev. Cell* **40**, 467–477.e5 (2017). 10.1016/j.devcel.2017.02.003 PMC5354100 28292425

[125] Damiano, E. R., Westheider, J., Tözeren, A. & Ley, K. Variation in the velocity, deformation and adhesion energy density of leukocytes rolling within venules. *Circ. Res.* **79**, 1122–1130 10.1161/01.RES.79.6.1122 (1996). 8943950

[126] Sundd, P., Pospieszalska, M. K. & Ley, K. Neutrophil rolling at high shear: flattening, catch bond behavior, tethers and slings. *Mol. Immunol.* **55**, 59–69 (2013). 10.1016/j.molimm.2012.10.025 PMC3601566 23141302

[127] Filippi, M.-D. Neutrophil transendothelial migration: updates and new perspectives. *Blood* **133**, 2149–2158 (2019). 10.1182/blood-2018-12-844605 PMC6524565 30898863

[128] Fine, N., Dimitriou, I. D. & Rottapel, R. Go with the flow: GEF-H1 mediated shear stress mechanotransduction in neutrophils. *Small GTPases* **11**, 23–31 (2020). 10.1080/21541248.2017.1332505 PMC6959313 29188751

[129] Grönloh, M. L. B., Arts, J. J. G. & van Buul, J. D. Neutrophil transendothelial migration hotspots - mechanisms and implications. *J. Cell Sci.* **134**, jcs255653 (2021). 10.1242/jcs.255653 33795378

[130] Moore, K. H., Murphy, H. A. & George, E. M. The glycocalyx: a central regulator of vascular function. *Am. J. Physiol. Regul. Integr. Comp. Physiol.* **320**, R508–R518 (2021). 10.1152/ajpregu.00340.2020 PMC8238147 33501896

[131] Bajénoff, M. et al. Stromal cell networks regulate lymphocyte entry, migration and territoriality in lymph nodes. *Immunity* **25**, 989–1001 (2006). 10.1016/j.immuni.2006.10.011 PMC2692293 17112751

[132] Meijering, E., Dzyubachyk, O. & Smal, I. Methods for cell and particle tracking. *Methods Enzymol.* **504**, 183–200 (2012). 10.1016/B978-0-12-391857-4.00009-4 22264535

[133] Tinevez, J.-Y. et al. TrackMate: an open and extensible platform for single-particle tracking. *Methods* **115**, 80–90 (2017). 10.1016/j.ymeth.2016.09.016 27713081

[134] Ershov, D. et al. TrackMate 7: integrating state-of-the-art segmentation algorithms into tracking pipelines. *Nat. Methods* **19**, 829–832 (2022). 10.1038/s41592-022-01507-1 35654950

[135] Schmidt, U., Weigert, M., Broaddus, C. & Myers, G. Cell detection with star-convex polygons. In *Medical Image Computing and Computer Assisted Intervention – MICCAI 2018*. *Lecture Notes in Computer Science* (eds Frangi, A. et al.) Vol. 11071 (Springer, 2018); 10.1007/978-3-030-00934-2_30

[136] McDonald, J. H. *Handbook of Biological Statistics* (Sparky House, 2014).

