# Supplementary material for: Tissue flow acts as a guidance cue for immune cell polarization and directional migration
Source: Nat Cell Biol. 2026 Sep 7;28(10):2008–26. doi: 10.1038/s41556-026-02058-9 (PMC13661273; doi:10.1038/s41556-026-02058-9)
Supplement: Supplementary file 1 [file 41556_2026_2058_MOESM1_ESM.pdf]

nature portfolio

Corresponding author(s): Roberto Mayor
Hoang Anh Le

Last updated by author(s): Nov 7, 2025

Reporting Summary

Nature Portfolio wishes to improve the reproducibility of the work that we publish. This form provides structure for consistency and transparency in reporting. For further information on Nature Portfolio policies, see our Editorial Policies and the Editorial Policy Checklist.

Statistics

For all statistical analyses, confirm that the following items are present in the figure legend, table legend, main text, or Methods section.

n/a	Confirmed	
☐	☒	The exact sample size (*n*) for each experimental group/condition, given as a discrete number and unit of measurement
☐	☒	A statement on whether measurements were taken from distinct samples or whether the same sample was measured repeatedly
☐	☒	The statistical test(s) used AND whether they are one- or two-sided<br>*Only common tests should be described solely by name; describe more complex techniques in the Methods section.*
☒	☐	A description of all covariates tested
☐	☒	A description of any assumptions or corrections, such as tests of normality and adjustment for multiple comparisons
☐	☒	A full description of the statistical parameters including central tendency (e.g. means) or other basic estimates (e.g. regression coefficient) AND variation (e.g. standard deviation) or associated estimates of uncertainty (e.g. confidence intervals)
☒	☐	For null hypothesis testing, the test statistic (e.g. *F*, *t*, *r*) with confidence intervals, effect sizes, degrees of freedom and *P* value noted<br>*Give P values as exact values whenever suitable.*
☒	☐	For Bayesian analysis, information on the choice of priors and Markov chain Monte Carlo settings
☒	☐	For hierarchical and complex designs, identification of the appropriate level for tests and full reporting of outcomes
☒	☐	Estimates of effect sizes (e.g. Cohen's *d*, Pearson's *r*), indicating how they were calculated

Our web collection on statistics for biologists contains articles on many of the points above.

Software and code

Policy information about availability of computer code

Data collection	For data collection, Leica software LAS X and Zeiss software Zen Black were used to collect fluorescence images. Microsoft Excel was used to document data points.
Data analysis	Software: For the majority of image analysis, Fiji software was used (current version 2.16.0/1.54p). For the exact plugins, check Materials and Methods. For PIV analysis, the PIVlab app (v3.06) in MATLAB (vR2024b 24.2.02740171) were used. For statistical analysis, graph plotting, RStudio version 4.2.1 and JupyterNotebook version 6.4.8 were used. For correlation analyses and modeling, all code is deposited on Github: https://github.com/WhoIsJack/ectoderm-myeloid-tracking-analysis.

For manuscripts utilizing custom algorithms or software that are central to the research but not yet described in published literature, software must be made available to editors and reviewers. We strongly encourage code deposition in a community repository (e.g. GitHub). See the Nature Portfolio guidelines for submitting code & software for further information.

Data

Policy information about availability of data

All manuscripts must include a data availability statement. This statement should provide the following information, where applicable:

- Accession codes, unique identifiers, or web links for publicly available datasets
- A description of any restrictions on data availability
- For clinical datasets or third party data, please ensure that the statement adheres to our policy

Source data are provided with this study. All other supporting data in this study are available within manuscript or from the corresponding author upon request. Codes for correlation analyses and modeling will be made available here: https://github.com/WhoIsJack/ectoderm-myeloid-tracking-analysis.

Research involving human participants, their data, or biological material

Policy information about studies with human participants or human data. See also policy information about sex, gender (identity/presentation), and sexual orientation and race, ethnicity and racism.

Reporting on sex and gender	N/A
Reporting on race, ethnicity, or other socially relevant groupings	N/A
Population characteristics	N/A
Recruitment	N/A
Ethics oversight	N/A

Note that full information on the approval of the study protocol must also be provided in the manuscript.

Field-specific reporting

Please select the one below that is the best fit for your research. If you are not sure, read the appropriate sections before making your selection.

☒ Life sciences ☐ Behavioural & social sciences ☐ Ecological, evolutionary & environmental sciences

For a reference copy of the document with all sections, see nature.com/documents/nr-reporting-summary-flat.pdf

Life sciences study design

All studies must disclose on these points even when the disclosure is negative.

Sample size	Initial sample size was determined following initial preliminary data collection as follow: the hypothetical number of explants (or embryos) needed to detect an effect size of 0.3 (medium) at a significance level (alpha) of 0.05 and a statistical power of 0.8 to be 175 explants (or embryos). Due to the impracticality of this number, and to reduce the use of animals in experimentation, as well as to capture the full spectrum of variables between individual cells, we decided to use the n number, which represents individual cells, for statistical analyses. The same number of cells were analysed for each explant wherever possible. To reduce the chance of a false positive and ensure the consistency and reproducibility of the observed effects, we drew our conclusions from experiments that were performed in multiple different contexts and setups, as well as from embryos collected from at least 3 different female frogs in separate days. We also consulted guidance from the Handbook of Biological Statistics for statistical tests. The exact n numbers were stated in figure legends.
Data exclusions	In Figure 4D, quality control excluded one outlier explant due to it being unhealthy even though the inclusion of this explant did not change the observed correlation. Every other explants were kept. This is stated in the Materials and Methods in the Trajectory deviation and speed correlation and cosine similarity analyses section.
Replication	To reduce the chance of a false positive and ensure the consistency and reproducibility of the observed effects, we drew our conclusions from experiments that were performed in multiple different contexts and setups, as well as from embryos collected from at least 3 different female frogs in separate days.
Randomization	Fertilised embryos were a mixture from at least 3 female frogs chosen at random from different days. Surviving embryos were randomly chosen with no internal bias. For any tracking of individual cells, cells were selected across the explant to ensure a good representation of that explant. Explant and embryos images were assigned with numbers to facilitate blind analysis whenever possible.
Blinding	Blinding was not always possible for morpholino treatment or imaging of whole embryos as this often result in changes in the embryo's morphology. For tracking of cells, wherever possible, explants were blinded with numbering before tracking began. However, this was not always possible as effects of different substrate coating or morpholino treatments could be quite visible at time. Nevertheless, we actively made an effort to perform blind analyses whenever possible.

Reporting for specific materials, systems and methods

We require information from authors about some types of materials, experimental systems and methods used in many studies. Here, indicate whether each material, system or method listed is relevant to your study. If you are not sure if a list item applies to your research, read the appropriate section before selecting a response.

Materials & experimental systems

n/a	Involved in the study
☐	☒ Antibodies
☒	☐ Eukaryotic cell lines
☒	☐ Palaeontology and archaeology
☐	☒ Animals and other organisms
☒	☐ Clinical data
☒	☐ Dual use research of concern
☒	☐ Plants

Methods

n/a	Involved in the study
☒	☐ ChIP-seq
☒	☐ Flow cytometry
☒	☐ MRI-based neuroimaging

Antibodies

Antibodies used	Anti Fibronectin 4H2 - Developmental Studies Hybridoma Bank<br>Fibronectin Monoclonal Antibody (A17) - Invitrogen
Validation	4H2 antibody has been used to detect fibronectin in Xenopus embryos in various studies before as found on the website: https://dshb.biology.uiowa.edu/4H2<br>A17 antibody can react to bovine fibronectin as shown in their website: https://www.thermofisher.com/antibody/product/Fibronectin-Antibody-clone-A17-Monoclonal/CSI%20005-17-02

Animals and other research organisms

Policy information about studies involving animals; ARRIVE guidelines recommended for reporting animal research, and Sex and Gender in Research

Laboratory animals	Adult female Xenopus laevis were used to obtain oocytes, which were fertilized with male sperm. Xenopus were age 2-5 years old.
Wild animals	N/A
Reporting on sex	N/A
Field-collected samples	N/A
Ethics oversight	Animal licenses were approved by the Animal Welfare and Ethical Review Board (WERB) at University College London and issued by the Home Office, UK.

Note that full information on the approval of the study protocol must also be provided in the manuscript.

Plants

Seed stocks	N/A
Novel plant genotypes	N/A
Authentication	N/A
