# Supplementary material for: Tissue flow acts as a guidance cue for immune cell polarization and directional migration
Source: Nat Cell Biol. 2026 Sep 7;28(10):2008–26. doi: 10.1038/s41556-026-02058-9 (PMC13661273; doi:10.1038/s41556-026-02058-9)
Supplement: Supplementary file 2 [file 41556_2026_2058_MOESM2_ESM.pdf]

nature portfolio

Peer Review File

Tissue flow acts as a guidance cue for immune cell polarisation and directional migration

Corresponding Author: Professor Roberto Mayor

Version 0:

Decision Letter:

*Please delete the link to your author homepage if you wish to forward this email to co-authors.

Dear Professor Mayor,

Your manuscript, "Tissue flow acts as a guidance cue for immune cell polarisation and directional migration", has now been seen by 3 referees, who are experts in biophysics (referee 1); Xenopus development and mechanobiology (referee 3); and immune cell migration (referee 4), as well as two early-career researcher co-reviewers. As you will see from their comments (attached below) they find this work of potential interest, but have raised substantial concerns, which in our view would need to be addressed with considerable revisions before we can consider publication in Nature Cell Biology.

Nature Cell Biology editors discuss the referee reports in detail within the editorial team, including the chief editor, to identify key referee points that should be addressed with priority, and requests that are overruled as being beyond the scope of the current study. To guide the scope of the revisions, I have listed these points below. We are committed to providing a fair and constructive peer-review process, so please feel free to contact me if you would like to discuss any of the referee comments further.

In particular, it would be essential to:

A) Provide further experimental evidence of the role of tissue flows guiding EM cell protrusion and migration, which may include direct mechanical manipulation (Reviewers #3 and #4) as well as clarify the quantitative modelling and interpretation (Reviewer #1)

B) Test with new experiments regarding potential contribution of fibronectin (Reviewer #3) and signalling (Reviewers #1 and #4)

C) Further clarify the relevance between the ex vivo explant system and how they correlate to in vivo conditions with additional experimental data (reviewer #3)

D) All other referee concerns pertaining to strengthening existing data, providing controls, methodological details, clarifications and textual changes, should also be addressed.

E) Finally please pay close attention to our guidelines on statistical and methodological reporting (listed below) as failure to do so may delay the reconsideration of the revised manuscript. In particular please provide:

- a Supplementary Figure including unprocessed images of all gels/blots in the form of a multi-page pdf file. Please ensure that blots/gels are labeled and the sections presented in the figures are clearly indicated.

- a Supplementary Table including all numerical source data in Excel format, with data for different figures provided as different sheets within a single Excel file. The file should include source data giving rise to graphical representations and statistical descriptions in the paper and for all instances where the figures present representative experiments of multiple independent repeats, the source data of all repeats should be provided.

We would be happy to consider a revised manuscript that would satisfactorily address these points, unless a similar paper is published elsewhere, or is accepted for publication in Nature Cell Biology in the meantime.

When revising the manuscript please:

- ensure that it conforms to our format instructions and publication policies (see below and https://www.nature.com/nature/for-authors).

- provide a point-by-point rebuttal to the full referee reports verbatim, as provided at the end of this letter.

- for any revision that includes light microscopy data, we ask our authors to please include a completed light microscopy reporting table https://www.nature.com/documents/Light_microscopy_reporting_table.xlsx to ensure the methods are described thoroughly. The table will be available to reviewers and ultimately published should the manuscript be accepted at the journal.

- provide the completed Reporting Summary (found here https://www.nature.com/documents/nr-reporting-summary.pdf). This is essential for reconsideration of the manuscript will be available to editors and referees in the event of peer review. For more information see http://www.nature.com/authors/policies/availability.html or contact me.

When submitting the revised version of your manuscript, please pay close attention to our href="https://www.nature.com/nature-portfolio/editorial-policies/image-integrity">Digital Image Integrity Guidelines. and to the following points below:

-- that unprocessed scans are clearly labelled and match the gels and western blots presented in figures.
-- that control panels for gels and western blots are appropriately described as loading on sample processing controls
-- all images in the paper are checked for duplication of panels and for splicing of gel lanes.

Finally, please ensure that you retain unprocessed data and metadata files after publication, ideally archiving data in perpetuity, as these may be requested during the peer review and production process or after publication if any issues arise.

Nature Cell Biology is committed to improving transparency in authorship. As part of our efforts in this direction, we are now requesting that all authors identified as 'corresponding author' on published papers create and link their Open Researcher and Contributor Identifier (ORCID) with their account on the Manuscript Tracking System (MTS), prior to acceptance. ORCID helps the scientific community achieve unambiguous attribution of all scholarly contributions. You can create and link your ORCID from the home page of the MTS by clicking on 'Modify my Springer Nature account'. For more information please visit please visit www.springernature.com/orcid.

This journal strongly supports public availability of data. Please place the data used in your paper into a public data repository, or alternatively, present the data as Supplementary Information. If data can only be shared on request, please explain why in your Data Availability Statement, and also in the correspondence with your editor. Please note that for some data types, deposition in a public repository is mandatory - more information on our data deposition policies and available repositories appears below.

Please submit the revised manuscript files and the point-by-point rebuttal to the referee comments using this link:

Link Redacted

*This url links to your confidential home page and associated information about manuscripts you may have submitted or be reviewing for us. If you wish to forward this email to co-authors, please delete the link to your homepage.

We would like to receive a revised submission within six months.

We hope that you will find our referees' comments, and editorial guidance helpful. Please do not hesitate to contact me if there is anything you would like to discuss.

Best wishes,

Daryl Jason David

Daryl Jason Verzosa David, PhD

Senior Editor, Nature Cell Biology
Advisory Editor, npj Biological Physics and Mechanics
Nature Portfolio

Heidelberger Platz 3, 14197 Berlin, Germany
Email: daryl.david@nature.com
ORCID: https://orcid.org/0000-0002-9253-4805

Reviewers' Comments:

Reviewer #1 (Remarks to the Author):

In this manuscript, the authors study the anterior Ventral Blood Island (aVBI) where a type of early immune like cell, embryonic myeloid (EM) cells, migrate radially away to protect the early embryo. These EM cells are embedded in an ectoderm tissue layer. The authors develop an explant system using Xenopus embryos where the tissue is removed and plated and the EM and ectoderm cells can be tracked radially migrating away from the explant. They find that if the ectoderm cells are removed, while the EM migration speed remains the same, the radial polarization is reduced, impairing the migration of the EM cells. They perform multiple additional experiments and perturbations to understand how the ectoderm cells guide the EM cells, ruling out compression and chemotaxis before demonstrating it is a coupling of EM cell polarization to ectoderm flow. The authors then develop a simple mathematical model for the coupling that reproduces important features of the experimental observations.

Overall, this is a well-written and very interesting paper with clear and careful experiments to justify its conclusions. In particular, the development and validation of a new explant system for studying EM cell migration, the clever quantitative analysis of cell shapes, trajectories, and motion correlations, and the optogenetic perturbations are all outstanding. The conclusion, that cell-on-cell crawling is cued by underlying tissue flows, is likely to be very high impact, and may drive other researchers to look for similar phenomena in their own systems.
There are a few issues we would like to ask to be clarified before publication. As our expertise in in modeling, we focus most of our questions on how the authors use modeling to understand the quantitative results.

Major concerns:
1. In Fig. 4 F i., the modeling results are plotted a separate panel from the experimental results. Is this for visual clarity or is there a reason they cannot be plotted together? For example, it is difficult to tell if the slopes are the same. Averages of the experimental data could be compared directly. If the slope of the model does not match, what is missing from the model that would adjust this? Qualitatively the trends are similar; the velocity increases with radial distance, up to the edge where it falls off, which does capture the qualitative trend in the experimental data. But if possible a direct quantitative comparison would be more convincing.

2. In Fig. 4 F ii., the model proposes that the EM velocity profile is correlated with the ectoderm profile, up until a saturation point. However, the experimental data is only within the region where the model has well saturated. Without any evidence of the shorter distances, it doesn't seem appropriate to speculate that the much on this region of the profile. Even if there is a boundary condition that the curve must start at 0, it could very well be a sigmoid or any other function.

3. While the observational data in Fig 5 is compelling and interesting, it seems that the quantitative evidence is focused only on one or two cells. In other words, there is no statistical data averaged over (even a small) ensemble of cells, and so it remains unclear whether this reorientation always happens in response to an optogenetic perturbation, or whether these one or two cells that are analyzed are especially sensitive. While we recognize these are incredibly difficult experiments, is there any way to get a sense of statistics for the response to perturbation? E.g., if you analyzed 3 cells in the same way you analyze Fig 5f, would they look similar?

4. Is there any evidence that there might also be a direct mechanical interaction between the EM cells and the ectoderm, not just a signaling that induces EM polarization? For example, if EM cells are strongly coupled via friction or adhesion to the ectoderm, one might expect that the flow of the ectoderm would drag the EM cells along, not just change their polarization. This would add a contribution that correlates the directionality at T=0 in Fig 4D. Clearly, Fig. 4D shows that this effect, if it exists, is sub-dominant to a 5-min-delayed, polarization-based mechanism, but can it be ruled out as a contribution?

Minor concerns:
5. It might be helpful to provide a little more clarity about the geometry of the tissue and the plate in the explant experiment. To us, it was a little unclear whether -- when explants are placed on the plate -- the EM cells crawling along the plate, or through the ectoderm. The cartoon of the embryo in Fig. 1 A makes the ectoderm look like a top layer with EM cells within the mesoderm, but the cartoon in Fig. 4 of the explant on the dish looks like the EM cells are just within the ectoderm.

6. If the EM cells are crawling within the ectoderm, when the ectoderm is removed, is part of the change due to cells crawling on just the dish compared to crawling inside a tissue?

Reviewer #2 (Remarks to the Author):

I co-reviewed this manuscript with one of the reviewers who provided the listed reports. This is part of the Nature Cell Biology initiative to facilitate training in peer review and to provide appropriate recognition for Early Career Researchers who co-review manuscripts.

Reviewer #3 (Remarks to the Author):

This manuscript investigates how primitive embryonic myeloid (EM) cells disperse during early Xenopus development. The authors proposed that EM cell migration is guided by ectodermal tissue flow, a result of the overall embryo elongation. The authors used both frog embryology approaches and newly developed explant culture assays to characterize cell movement and concluded that local ectodermal tissue flow can reorient EM cell protrusions and bias their migration direction. The study also noted that EM cells can switch between more lamellipodial and amoeboid morphologies, though this aspect is only briefly discussed.

Overall, this study addresses an important, underexplored, and underappreciated form of cell movement, the cell-on-cell migration, rather than cell-on-ECM migration, and provides a valuable framework for understanding how collective tissue dynamics can influence other cells around them.
However, I find that the central conclusions regarding tissue flow require additional supporting analysis and clarification of some ambiguities in the experimental setup and data interpretation.
While the discussion section does a good job of acknowledging alternative interpretations, the results as presented are not yet described clearly enough or supported by sufficient quantitative evidence. I therefore recommend a major revision.

Three major concerns:

1. Lack of baseline correlation between ectodermal tissue flow and myeloid cell migration.

The central claim of this study is that EM cell dispersion is guided by ectodermal tissue flow during embryo elongation. However, the current manuscript does not clearly establish this relationship in unperturbed wild-type embryos. Demonstrating a baseline correlation between ectodermal flow and EM cell trajectories under normal conditions could significantly strengthen the conclusion and seems to be straightforward to do.

While Figure 7E suggests a slight indication of this relationship, it was not quantified or analyzed systematically. Moreover, the accompanying Video 23 shows a burst of EM cells migrating towards the anterior-dorsal/lower-left after the tissue flow slowed down. The authors should perform an embryo-wide correlation analysis between the ectodermal flow and EM migration in control embryos to establish this foundational relationship. The same analysis could be performed for all in vivo perturbations, similar to what has been performed in the explants.

Additionally, given that ectodermal flow is likely spatially complex in both control and perturbed embryos, imposing a more defined flow could also be informative. For example, mechanically stretching embryos or explants along the head-to-tail axis, or orthogonally, might help create a clearer flow field and provide an additional layer of evidence supporting the authors’ conclusion.

2. Unclear explant setup and its correspondence to in vivo EM cell positioning

I feel it is difficult to visualize how the described explant recapitulates the in vivo arrangement of ectoderm, mesoderm, and EM cells. In the explant setup, it seems that the authors isolated ectoderm and mesoderm together and cultured them on a fibronectin-coated dish, with the mesoderm placed downward and the ectoderm on top. However, it is unclear to me why in Fig. 4E the ectoderm forms a bump in the center, the mesoderm layer was excluded, and the EM cells were dispersed within the ectoderm.

Additionally, according to Fig 1A and SFig 1D, in vivo EM cells first migrate underneath the ectoderm within the mesoderm, and then penetrate deeper into the endoderm. However, within the explant, the ectodermal layer appears to spread rapidly onto the fibronectin-coated substrate, retaining the mesodermal cells at the center. Thus, upon the onset of EM cell migration, they entered the ectoderm directly (SFig. 1C), rather than migrating within the mesoderm sandwiched by two fibronectin layers.

This setup is confusing as presented. The authors should provide a clearer schematic and text description of the explant configuration, and more importantly, discuss how this arrangement reproduces the in vivo environment. If the difference is apparently substantial, the authors should also clarify which aspects of the ex vivo behavior can be meaningfully translated to the in vivo situation.

3. Clarification of fibronectin distribution and its role in EM cell migration in vivo

In the Introduction, the authors state that “Only a thin layer of fibronectin separates the ectoderm from the mesoderm, and the mesoderm from the endoderm30–32, making EM cell dispersion primarily a cell-on-cell migration process”. However, this statement appears inconsistent with SFig. 1D and published data as in Davidson et al., Dev Dyn, 2004.

The aforementioned fibronectin layers appear reasonably dense. Because fibronectin fibrils can be stretched and remodeled by cell contractility and the fibronectin network can be patterned by tissue-scale cell flows, the ectodermal cell movements described in this work may similarly organize the fibronectin network. I am prone to believe that the ectodermal flow guides EM cell migration partially through fibronectin.

I recommend that the authors reassess this aspect through both a literature review and experiments to clarify the potential role of fibronectin.

Minor concerns:

1. The transition between more lamellipodial and amoeboid morphologies of the myeloid cells is very interesting. Could the authors guess which aspects of ectodermal tissue dynamics might influence this transition?

2. The optogenetics data seem very promising. When looking at Video 19, an apparent ectodermal cell flow was triggered, and the EM cell below was repolarized very quickly. However, I also noticed that other EM cells around the activation spot tended to respond differently. For example, the right EM cell moved away and against the flow, and a few cells above that that formed doublets remained static. Could this be distance, flow speed, or morphology-dependent? How might this heterogeneity affect EM dispersion in vivo?

3. Fig. 1B does not seem to be at the 32-cell stage, but a little later compared with Ref 33. Does this timing provide a better labeling of EM cells?

Reviewer #4 (Remarks to the Author):

The manuscript by Anh Le et al. investigates how tissue movements influence single cell migration. Specifically, they explore the mechanism by which embryonic myeloid (EM) cells migrate during the early stages of development. The authors employ an ex vivo culture system using tissue explants from Xenopus laevis to show that EM cells move less directionally in the absence of the ectoderm. The ectodermal layer also contributed to polarity of the EM cells, as measured by the localisation of cytoskeletal markers. Physical compression or response to chemotactic cues were not identified as the reason for the change in the polarity and migration of EM cells that were cultured without the ectoderm. Optogenetic manipulation of ectodermal tissue flows perturbed the migration of EM cells, indicating that ectodermal tissue movement play a role. Furthermore, genetic disruption of ectodermal tissue movement and mechanical restriction of tissue elongation reduced the dispersal of EM cells. Regarding novelty, other mechanisms have been described that help single cells spread in multicellular tissues, but the notion that tissue movements play a role has been less explored. The paper uses elegant approaches like optogenetics and mechanical manipulations to causally link tissue movements with the behaviour of EM cells. The findings are interesting and novel, but the mechanisms involved could be further clarified, to understand whether the effects are purely mechanical in nature or act via chemical pathways.

Major points

-An important point to address is the possibility that tissue flow influences EM movement via chemical signalling versus direct mechanical signalling. For example, ectodermal cell flow could change the distribution pattern of a chemical ligand (produced by ectoderm or EM cells) on the ectoderm surface. In terms of addressing the possibility of chemotaxis, the authors attempted one pathway but there are more general perturbations that can be tested. For example, inhibition by pertussis toxin could be used to block Gai signalling, without needing to identify the specific ligand/receptor. In addition, supernatants from ectoderm can be collected, concentrated and then added to the EM cells. The authors could also consider candidates from the literature for which there is evidence for a role in EM spreading. In zebrafish, m-csf plays a key role in macrophage early invasion of different tissues. https://doi.org/10.1006/dbio.2001.0393. It could be that tissue flows influence distribution of such ligands.

-In relation to the above, the experiments with liquid flushing are informative for soluble ligands, but do not exclude chemoattractant ligands (e.g. chemokines) that can immobilise on the surface of the ectoderm. Heparan sulfate is a common cell surface sugar on which chemokines can immobilise and can be inhibited by heparinase treatment.

Minor points

-In the introduction, it would be helpful to summarise other ways of single cell spreading, e.g. through contact inhibition of locomotion (doi: 10.1016/j.cell.2015.02.015), dispersion through self-generated gradients (https://doi.org/10.1371/journal.pbio.1001966) etc. This would allow the reader to relate the findings to other mechanisms and better appreciate the novel aspect of tissue flow as a factor.

-It is important to mention more studies that investigate cell-on-cell migration to better distinguish the advance made here and indicate the broader relevance of the findings. One example of relevance is the migration of immune cells on fibroblast networks within lymphoid organs (https://doi.org/10.1016/j.immuni.2010.04.017, and 10.1016/j.immuni.2006.10.011). Here leukocytes use stable fibroblast network for contact guidance. Interestingly, immobilised chemokines on the fibroblasts play a role in guiding these migration patterns. The authors could highlight the novel aspect of tissue movement as a factor and that discuss whether chemical ligands could also be influenced.

- 'Early Xenopus embryos lack a functional circulatory system, so EM cells migrate beneath the outer ectodermal layer, between mesodermal cells, and later invade into the deeper endodermal tissue'. This phrase suggests that this circulation-independent dispersion pattern is specific to Xenopus. Yet EM cells in other organisms also disperse prior to circulation onset https://doi.org/10.1242/dev.126.17.3735. In addition, other models exist where cells spread without circulation. It would be helpful to emphasise more the broader relevance on the phenomenon rather than restrict the relevance to Xenopus.

-The figures are generally well-presented, and the information is mostly sufficient. In Fig. 2 some informatio is missing on the number of embryos used for panel c and the number of cells and embryos in panel d.

-In Fig. 3 the authors should ideally include the intact – compressed condition and fragmented-non compressed condition to facilitate interpretation of the datasets.

-Fig. 5 shows optogenetic experiments that are important for mechanistic understanding. Some quantification for imaging related to f and i is needed to support the claims. In addition, the authors can include controls showing the effect of photoactivation in explants not expressing the ontogenetic construct. The optogenetic activation seems of small scale, i.e. not enough to recapitulate the tissue scale flows. Is it possible to do the optogenetic activation in multiple points/cells to achieve bigger scale and potentially clearer effects?

-In experiments of Fig. 6 how do we know the genetic modification does not change something secreted by the cells? Here it would be relevant to investigate gene expression changes or functionally test supernatants from these cells.

-The data presentation is generally good but some minor points here: In Fig. 5, some annotations inside images and scale bar are of low resolution and not clearly visible. In Fig. 6 membrane RFP si labelled in blue and can be confusing, perhaps a neutral white colour might be clearer. The videos are very helpful and well-presented, but it would be practical to consolidate some of them into fewer movies.

-The authors can discuss some additional points in the discussion section, such as alternative interpretations of the data. For example, is it possible that the fragmentation process could have effect on gene expression and differentiation of the EM cells and how could this be accounted for? And how could other tissue factors like the endoderm, further influence the migration pattern of EM cells?

-In the discussion section, the authors relate their observations to the extravasation process, where cells flatten due to changes in integrins downstream of chemokine activation. Here again, they can consider discussing a link to chemoattractant patterning and the presentation/immobilisation of chemokines on tissue surfaces.

Reviewer #5 (Remarks to the Author):

I co-reviewed this manuscript with one of the reviewers who provided the listed reports. This is part of the Nature Cell Biology initiative to facilitate training in peer review and to provide appropriate recognition for Early Career Researchers who co-review manuscripts.

GUIDELINES FOR MANUSCRIPT SUBMISSION TO NATURE CELL BIOLOGY

READABILITY OF MANUSCRIPTS – Nature Cell Biology is read by cell biologists from diverse backgrounds, many of whom are not native English speakers. Authors should aim to communicate their findings clearly, explaining technical jargon that might be unfamiliar to non-specialists, and avoiding non-standard abbreviations. Titles and abstracts should concisely communicate the main findings of the study, and the background, rationale, results and conclusions should be clearly explained in the manuscript in a manner accessible to a broad cell biology audience. Nature Cell Biology uses British spelling.

MANUSCRIPT FORMAT – please follow the guidelines listed in our Guide to Authors regarding manuscript formats at Nature Cell Biology.

TITLE – should be no more than 100 characters including spaces, without punctuation and avoiding technical terms, abbreviations, and active verbs..

AUTHOR NAMES – should be given in full.

AUTHOR AFFILIATIONS – should be denoted with numerical superscripts (not symbols) preceding the names. Full addresses should be included, with US states in full and providing zip/post codes. The corresponding author is denoted by: "Correspondence should be addressed to [initials]."

ABSTRACT AND MAIN TEXT – please follow the guidelines that are specific to the format of your manuscript, as listed in our Guide to Authors (http://www.nature.com/ncb/pdf/ncb_gta.pdf) Briefly, Nature Cell Biology Articles, Resources and Technical Reports have 3500 words, including a 150 word abstract, and the main text is subdivided in Introduction, Results, and Discussion sections. Nature Cell Biology Letters have up to 2500 words, including a 180 word introductory paragraph

(abstract), and the text is not subdivided in sections.

ACKNOWLEDGEMENTS – should be kept brief. Professional titles and affiliations are unnecessary. Grant numbers can be listed.

AUTHOR CONTRIBUTIONS – must be included after the Acknowledgements, detailing the contributions of each author to the paper (e.g. experimental work, project planning, data analysis etc.). Each author should be listed by his/her initials.

FINANCIAL AND NON-FINANCIAL COMPETING INTERESTS – the authors must include one of three declarations: (1) that they have no financial and non-financial competing interests; (2) that they have financial and non-financial competing interests; or (3) that they decline to respond, after the Author Contributions section. This statement will be published with the article, and in cases where financial and non-financial competing interests are declared, these will be itemized in a web supplement to the article. For further details please see https://www.nature.com/licenceforms/nrg/competing-interests.pdf.

REFERENCES – are limited to a total of 70 for Articles, Resources, Technical Reports; and 40 for Letters. This includes references in the main text and Methods combined. References must be numbered sequentially as they appear in the main text, tables and figure legends and Methods and must follow the precise style of Nature Cell Biology references. References only cited in the Methods should be numbered consecutively following the last reference cited in the main text. References only associated with Supplementary Information (e.g. in supplementary legends) do not count toward the total reference limit and do not need to be cited in numerical continuity with references in the main text. Only published papers can be cited, and each publication cited should be included in the numbered reference list, which should include the manuscript titles. Footnotes are not permitted.

METHODS – Nature Cell Biology publishes methods online. The methods section should be provided as a separate Word document, which will be copyedited and appended to the manuscript PDF, and incorporated within the HTML format of the paper.

Methods should be written concisely, but should contain all elements necessary to allow interpretation and replication of the results. As a guideline, Methods sections typically do not exceed 3,000 words. The Methods should be divided into subsections listing reagents and techniques. When citing previous methods, accurate references should be provided and any alterations should be noted. Information must be provided about: antibody dilutions, company names, catalogue numbers and clone numbers for monoclonal antibodies; sequences of RNAi and cDNA probes/primers or company names and catalogue numbers if reagents are commercial; cell line names, sources and information on cell line identity and authentication. Animal studies and experiments involving human subjects must be reported in detail, identifying the committees approving the protocols. For studies involving human subjects/samples, a statement must be included confirming that informed consent was obtained. Statistical analyses and information on the reproducibility of experimental results should be provided in a section titled “Statistics and Reproducibility”.

All Nature Cell Biology manuscripts submitted on or after March 21 2016 must include a Data availability statement as a separate section after Methods but before references, under the heading "Data Availability”. . For Springer Nature policies on data availability see http://www.nature.com/authors/policies/availability.html; for more information on this particular policy see http://www.nature.com/authors/policies/data/data-availability-statements-data-citations.pdf. The Data availability statement should include:

• Accession codes for primary datasets (generated during the study under consideration and designated as "primary accessions") and secondary datasets (published datasets reanalysed during the study under consideration, designated as "referenced accessions"). For primary accessions data should be made public to coincide with publication of the manuscript. A list of data types for which submission to community-endorsed public repositories is mandated (including sequence, structure, microarray, deep sequencing data) can be found here http://www.nature.com/authors/policies/availability.html#data.

• Unique identifiers (accession codes, DOIs or other unique persistent identifier) and hyperlinks for datasets deposited in an approved repository, but for which data deposition is not mandated (see here for details http://www.nature.com/sdata/data-policies/repositories).

• At a minimum, please include a statement confirming that all relevant data are available from the authors, and/or are included with the manuscript (e.g. as source data or supplementary information), listing which data are included (e.g. by figure panels and data types) and mentioning any restrictions on availability.

• If a dataset has a Digital Object Identifier (DOI) as its unique identifier, we strongly encourage including this in the Reference list and citing the dataset in the Methods.

We recommend that you upload the step-by-step protocols used in this manuscript to protocols.io. More details can found at https://www.protocols.io/help/publish-articles.

DISPLAY ITEMS – main display items are limited to 6-8 main figures and/or main tables for Articles, Resources, Technical Reports; and 5 main figures and/or main tables for Letters. For Supplementary Information see below.

FIGURES – Colour figure publication costs $600 for the first, and $300 for each subsequent colour figure. All panels of a multi-panel figure must be logically connected and arranged as they would appear in the final version. Unnecessary figures and figure panels should be avoided (e.g. data presented in small tables could be stated briefly in the text instead).

All imaging data should be accompanied by scale bars, which should be defined in the legend.
Cropped images of gels/blots are acceptable, but need to be accompanied by size markers, and to retain visible background signal within the linear range (i.e. should not be saturated). The boundaries of panels with low background have to be demarked with black lines. Splicing of panels should only be considered if unavoidable, and must be clearly marked on the figure, and noted in the legend with a statement on whether the samples were obtained and processed simultaneously. Quantitative comparisons between samples on different gels/blots are discouraged; if this is unavoidable, it should only be performed for samples derived from the same experiment with gels/blots were processed in parallel, which needs to be stated in the legend.

Figures should be provided at approximately the size that they are to be printed at (single column is 86 mm, double column is 170 mm) and should not exceed an A4 page (8.5 x 11"). Reduction to the scale that will be used on the page is not necessary, but multi-panel figures should be sized so that the whole figure can be reduced by the same amount at the smallest size at which essential details in each panel are visible. In the interest of our colour-blind readers we ask that you avoid using red and green for contrast in figures. Replacing red with magenta and green with turquoise are two possible colour-safe alternatives. Lines with widths of less than 1 point should be avoided. Sans serif typefaces, such as Helvetica (preferred) or Arial should be used. All text that forms part of a figure should be rewritable and removable.

We accept files from the following graphics packages in either PC or Macintosh format:

- For line art, graphs, charts and schematics we prefer Adobe Illustrator (.AI), Encapsulated PostScript (.EPS) or Portable Document Format (.PDF). Files should be saved or exported as such directly from the application in which they were made, to allow us to restyle them according to our journal house style.

- We accept PowerPoint (.PPT) files if they are fully editable. However, please refrain from adding PowerPoint graphical effects to objects, as this results in them outputting poor quality raster art. Text used for PowerPoint figures should be Helvetica (preferred) or Arial.

- We do not recommend using Adobe Photoshop for designing figures, but we can accept Photoshop generated (.PSD or .TIFF) files only if each element included in the figure (text, labels, pictures, graphs, arrows and scale bars) are on separate layers. All text should be editable in 'type layers' and line-art such as graphs and other simple schematics should be preserved and embedded within 'vector smart objects' - not flattened raster/bitmap graphics.

- Some programs can generate Postscript by 'printing to file' (found in the Print dialogue). If using an application not listed above, save the file in PostScript format or email our Art Editor, Allen Beattie for advice (a.beattie@nature.com).

Regardless of format, all figures must be vector graphic compatible files, not supplied in a flattened raster/bitmap graphics format, but should be fully editable, allowing us to highlight/copy/paste all text and move individual parts of the figures (i.e. arrows, lines, x and y axes, graphs, tick marks, scale bars etc.). The only parts of the figure that should be in pixel raster/bitmap format are photographic images or 3D rendered graphics/complex technical illustrations.

All placed images (i.e. a photo incorporated into a figure) should be on a separate layer and independent from any superimposed scale bars or text. Individual photographic images must be a minimum of 300+ DPI (at actual size) or kept constant from the original picture acquisition and not decreased in resolution post image acquisition. All colour artwork should be RGB format.

EXTENDED DATA FIGURES - When re-submitting your manuscript, please ensure that any supplementary figures and tables that are crucial to the manuscript's conclusions are converted into Extended Data figures and tables to increase visibility of these data. Extended Data figures and tables are online-only (present in the online PDF and full-text HTML versions of the paper), peer-reviewed display items that provide essential background to the article but are not included in the main article due to space constraints. A maximum of ten Extended Data display items (figures and tables) is permitted.

FIGURE LEGENDS – must not exceed 350 words for each figure to allow fit on a single printed NCB page together with the figure. They must include a brief title for the whole figure, and short descriptions of each panel with definitions of the symbols used, but without detailing methodology.

TABLES – main tables should be provided as individual Word files, together with a brief title and legend. For supplementary tables see below.

SUPPLEMENTARY INFORMATION – Supplementary information is material directly relevant to the conclusion of a paper, but which cannot be included in the printed version in order to keep the manuscript concise and accessible to the general reader. Supplementary information is an integral part of a Nature Cell Biology publication and should be prepared and presented with as much care as the main display item, but it must not include non-essential data or text, which may be removed at the editor's discretion. All supplementary material is fully peer-reviewed and published online as part of the HTML version of the manuscript. Supplementary Figures and Supplementary Notes are appended at the end of the main PDF of the published manuscript.

Supplementary items should relate to a main text figure, wherever possible, and should be mentioned sequentially in the main manuscript, designated as Supplementary Figure, Table, Video, or Note, and numbered continuously (e.g. Supplementary Figure 1, Supplementary Figure 2, Supplementary Table 1, Supplementary Table 2 etc.).

Unprocessed scans of all key data generated through electrophoretic separation techniques need to be presented in a supplementary figure that should be labelled and numbered as the final supplementary figure, and should be mentioned in every relevant figure legend. This figure does not count towards the total number of figures and is the only figure that can be displayed over multiple pages, but should be provided as a single file, in PDF or TIFF format. Data in this figure can be displayed in a relatively informal style, but size markers and the figures panels corresponding to the presented data must be indicated.

The total number of Supplementary Figures (not including the “unprocessed scans” Supplementary Figure) should not exceed the number of main display items (figures and/or tables (see our Guide to Authors and March 2012 editorial http://www.nature.com/ncb/authors/submit/index.html#suppinfo; http://www.nature.com/ncb/journal/v14/n3/index.html#ed). No restrictions apply to Supplementary Tables or Videos, but we advise authors to be selective in including supplemental data.

Each Supplementary Figure should be provided as a single page and as an individual file in one of our accepted figure formats and should be presented according to our figure guidelines (see above). Supplementary Tables should be provided as individual Excel files. Supplementary Videos should be provided as .avi or .mov files up to 50 MB in size. Supplementary Figures, Tables and Videos much be accompanied by a separate Word document including titles and legends.

GUIDELINES FOR EXPERIMENTAL AND STATISTICAL REPORTING

REPORTING REQUIREMENTS – We are trying to improve the quality of methods and statistics reporting in our papers. To that end, we are now asking authors to complete a reporting summary that collects information on experimental design and reagents. The Reporting Summary can be found here https://www.nature.com/documents/nr-reporting-summary.pdf)If you would like to reference the guidance text as you complete the template, please access these flattened versions at http://www.nature.com/authors/policies/availability.html.

STATISTICS – Wherever statistics have been derived the legend needs to provide the n number (i.e. the sample size used to derive statistics) as a precise value (not a range), and define what this value represents. Error bars need to be defined in the legends (e.g. SD, SEM) together with a measure of centre (e.g. mean, median). Box plots need to be defined in terms of minima, maxima, centre, and percentiles. Ranges are more appropriate than standard errors for small data sets. Wherever statistical significance has been derived, precise p values need to be provided and the statistical test used needs to be stated in the legend. Statistics such as error bars must not be derived from n<3. For sample sizes of n<5 please plot the individual data points rather than providing bar graphs. Deriving statistics from technical replicate samples, rather than biological replicates is strongly discouraged. Wherever statistical significance has been derived, precise p values need to be provided and the statistical test stated in the legend.

Information on how many times each experiment was repeated independently with similar results needs to be provided in the legends and/or Methods for all experiments, and in particular wherever representative experiments are shown.

We strongly recommend the presentation of source data for graphical and statistical analyses as a separate Supplementary Table, and request that source data for all independent repeats are provided when representative experiments of multiple independent repeats, or averages of two independent experiments are presented. This supplementary table should be in Excel format, with data for different figures provided as different sheets within a single Excel file. It should be labelled and numbered as one of the supplementary tables, titled “Statistics Source Data”, and mentioned in all relevant figure legends.

--------- Please don't hesitate to contact NCB@nature.com should you have queries about any of the above requirements ---------

Version 1:

Decision Letter:

Our ref: NCB-A60247A

16th June 2026

Dear Dr. Mayor,

Thank you for submitting your revised manuscript "Tissue flow acts as a guidance cue for immune cell polarisation and directional migration" (NCB-A60247A). It has now been seen by the original referees and their comments are below. The reviewers find that the paper has improved in revision, and therefore we'll be happy in principle to publish it in Nature Cell

Biology, pending minor revisions to satisfy the referees' final requests and to comply with our editorial and formatting guidelines.

The current version of your manuscript is in a PDF format. Therefore, please email us a copy of the file in an editable format (Microsoft Word or LaTex)-- we can not proceed with PDFs at this stage.

We are now performing detailed checks on your paper and will send you a checklist detailing our editorial and formatting requirements in a couple of weeks. Please do not upload the final materials and make any revisions until you receive this additional information from us.

Thank you again for your interest in Nature Cell Biology. Please do not hesitate to contact me if you have any questions.

Sincerely,

Daryl

Daryl Jason Verzosa David, PhD

Senior Editor, Nature Cell Biology
Advisory Editor, npj Biological Physics and Mechanics
Nature Portfolio

Heidelberger Platz 3, 14197 Berlin, Germany
Email: daryl.david@nature.com
ORCID: https://orcid.org/0000-0002-9253-4805

Reviewer #1 (Remarks to the Author):

Overall, we believe the authors have adequately addressed our concerns and those of the other reviewers. The theoretical model with the added source term now agrees quantitatively with the experimental data, which substantially improves the impact. For visual clarity the authors may want to consider using open markers for the data in the model-data comparison panels, as we found the combination a bit difficult to parse. The improved statistics and additional optogenetic experiments provide support to the authors claims. We appreciate the expanded discussion on the passive effects of friction vs active polarization and believe the cartoons are much improved. We now recommend publication.

Reviewer #2 (Remarks to the Author):

I co-reviewed this manuscript with one of the reviewers who provided the listed reports. This is part of the Nature Cell Biology initiative to facilitate training in peer review and to provide appropriate recognition for Early Career Researchers who co-review manuscripts.

Reviewer #3 (Remarks to the Author):

I appreciate the authors' careful and thorough responses to these comments. The revised version addresses my major concerns in a clear and constructive manner.

In particular, I appreciate the significant effort the authors have made to add new experiments, test alternative approaches, and clarify points that were confusing to me in the original submission. These additions have strengthened the manuscript and made the interpretation of the results more balanced and better supported.

Reviewer #4 (Remarks to the Author):

The authors have performed a careful and comprehensive revision that has addressed my comments. The additional experiments and quantifications are helpful in interpreting the findings and the editorial changes have improved the clarity of the manuscript. I also appreciate the additional discussion on alternative interpretations of data.

I only have a few minor comments/suggestions:
-The PTX/anti-CSF treatments had small effects but do not appear insignificant. It is hard to exclude incomplete inhibition in such experiments, so the authors could articulate the limitations of these perturbations.
- The experiments with heparinase revealed a marked influence. The authors can discuss these findings also in relation to haptotactic chemokine gradients that have been shown to be sensitive to heparinase treatment (10.1126/science.1228456; 10.1016/j.cub.2012.11.018).

Reviewer #5 (Remarks to the Author):

I co-reviewed this manuscript with one of the reviewers who provided the listed reports. This is part of the Nature Cell Biology initiative to facilitate training in peer review and to provide appropriate recognition for Early Career Researchers who co-review manuscripts.

INTERNAL USE ONLY AIP modules required: ["Clinical","Life Science"]

Version 2:

Decision Letter:

Dear Dr Mayor,

I am pleased to inform you that your manuscript, "Tissue flow acts as a guidance cue for immune cell polarisation and directional migration", has now been accepted for publication in Nature Cell Biology.

Thank you for sending us the final manuscript files to be processed for print and online production, and for returning the manuscript checklists and other forms. Your manuscript will now be passed to our production team who will be in contact with you if there are any questions with the production quality of supplied figures and text.

Over the next few weeks, your paper will be copyedited to ensure that it conforms to Nature Cell Biology style. Once your paper is typeset, you will receive an email with a link to choose the appropriate publishing options for your paper and our Author Services team will be in touch regarding any additional information that may be required.

After the grant of rights is completed, you will receive a link to your electronic proof via email with a request to make any corrections within 48 hours. If, when you receive your proof, you cannot meet this deadline, please inform us at rjsproduction@springernature.com immediately.

You will not receive your proofs until the publishing agreement has been received through our system.

Due to the importance of these deadlines, we ask that you please let us know now whether you will be difficult to contact over the next month. If this is the case, we ask you provide us with the contact information (email, phone and fax) of someone who will be able to check the proofs on your behalf, and who will be available to address any last-minute problems.

If you have any questions about our publishing options, costs, Open Access requirements, or our legal forms, please contact ASJournals@springernature.com

Once your paper has been scheduled for online publication, the Nature press office will be in touch to confirm the details. An online order form for reprints of your paper is available at https://www.nature.com/reprints/author-reprints.html. All co-authors, authors' institutions and authors' funding agencies can order reprints using the form appropriate to their geographical region.

Publication is conditional on the manuscript not being published elsewhere and on there being no announcement of this work to any media outlet until the online publication date in Nature Cell Biology.

You may wish to make your media relations office aware of your accepted publication, in case they consider it appropriate to organize some internal or external publicity. Once your paper has been scheduled you will receive an email confirming the publication details. This is normally 3-4 working days in advance of publication. If you need additional notice of the date and time of publication, please let the production team know when you receive the proof of your article to ensure there is sufficient time to coordinate. Further information on our embargo policies can be found here: https://www.nature.com/authors/policies/embargo.html

Please note that *Nature Cell Biology* is a Transformative Journal (TJ). Authors may publish their research with us through the traditional subscription access route or make their paper immediately open access through payment of an article-processing charge (APC). Authors will not be required to make a final decision about access to their article until it has been accepted. Find out more about Transformative Journals

Authors may need to take specific actions to achieve compliance with funder and institutional open access mandates. If your research is supported by a funder that requires immediate open access (e.g. according to Plan S principles or the NIH public access policy) then

you should select the gold OA route, and we will direct you to the compliant route where possible. Because authors warrant under our subscription licensing terms that they haven't committed to licensing any version of their article under a licence inconsistent with the terms of our agreement – including the applicable embargo period – publication under the subscription model isn't suitable for authors whose funders require no embargo.

To assist our authors in disseminating their research to the broader community, our SharedIt initiative provides you with a unique shareable link that will allow anyone (with or without a subscription) to read the published article. Recipients of the link with a subscription will also be able to download and print the PDF.

If your paper includes color figures, please be aware that in order to help cover some of the additional cost of four-color reproduction, Nature Portfolio charges our authors a fee for the printing of their color figures. Please contact our offices for exact pricing and details.

As soon as your article is published, you will receive an automated email with your shareable link.

If you have not already done so, we strongly recommend that you upload the step-by-step protocols used in this manuscript to protocols.io (https://protocols.io), an open online resource that allows researchers to share their detailed experimental know-how. All uploaded protocols are made freely available and are assigned DOIs for ease of citation. Protocols and Nature Portfolio journal papers in which they are used can be linked to one another, and this link is clearly and prominently visible in the online versions of both. Authors who performed the specific experiments can act as primary authors for the Protocol as they will be best placed to share the methodology details, but the Corresponding Author of the present research paper should be included as one of the authors. By uploading your Protocols onto protocols.io, you are enabling researchers to more readily reproduce or adapt the methodology you use, as well as increasing the visibility of your protocols and papers. You can also establish a dedicated workspace to collect your lab Protocols. Further information can be found at https://www.protocols.io/help/publish-articles.

You can use a single sign-on for all your accounts, view the status of all your manuscript submissions and reviews, access usage statistics for your published articles and download a record of your refereeing activity for the Nature Portfolio.

Nature Cell Biology encourages authors presenting evidence for cell, biological, molecular, and genetic interactions to consider communicating these findings using Biofactoid (https://biofactoid.org/). This tool helps users share a searchable representation of interactions (e.g. binding, gene expression, post-translational modification) between genes, gene products, or chemicals. Information added to Biofactoid, with author attribution, is shared on social media and public databases, such as Pathway Commons, where it can be discovered and analyzed in the context of a large and growing corpus of knowledge.

Please feel free to contact us if you have any questions.

With kind regards,
Daryl

Daryl Jason Verzosa David, PhD

Senior Editor, Nature Cell Biology
Advisory Editor, npj Biological Physics and Mechanics
Nature Portfolio

Heidelberger Platz 3, 14197 Berlin, Germany
Email: daryl.david@nature.com
ORCID: https://orcid.org/0000-0002-9253-4805

Click here if you would like to recommend Nature Cell Biology to your librarian
http://www.nature.com/subscriptions/recommend.html#forms

** Visit the Springer Nature Editorial and Publishing website at www.springernature.com/editorial-and-publishing-jobs for more information about our career opportunities. If you have any questions please click here.**

Open Access This Peer Review File is licensed under a Creative Commons Attribution 4.0 International License, which permits use, sharing, adaptation, distribution and reproduction in any medium or format, as long as you give appropriate credit to the original author(s) and the source, provide a link to the Creative Commons license, and indicate if changes were made.
In cases where reviewers are anonymous, credit should be given to 'Anonymous Referee' and the source.
The images or other third party material in this Peer Review File are included in the article's Creative Commons license, unless indicated otherwise in a credit line to the material. If material is not included in the article's Creative Commons license and your intended use is not permitted by statutory regulation or exceeds the permitted use, you will need to obtain permission directly from the copyright holder.
To view a copy of this license, visit https://creativecommons.org/licenses/by/4.0/

Rebuttal letter

We sincerely thank the reviewers for their thoughtful and constructive feedback. Their insightful comments have helped us improve the clarity, rigour, and overall quality of our manuscript. In response, we have conducted additional experiments, which we believe have significantly strengthened the work. Below, we address each comment in detail and our responses are highlighted in red for clarity.

Reviewer #1 (Remarks to the Author):

In this manuscript, the authors study the anterior Ventral Blood Island (aVBI) where a type of early immune like cell, embryonic myeloid (EM) cells, migrate radially away to protect the early embryo. These EM cells are embedded in an ectoderm tissue layer. The authors develop an explant system using Xenopus embryos where the tissue is removed and plated and the EM and ectoderm cells can be tracked radially migrating away from the explant. They find that if the ectoderm cells are removed, while the EM migration speed remains the same, the radial polarization is reduced, impairing the migration of the EM cells. They perform multiple additional experiments and perturbations to understand how the ectoderm cells guide the EM cells, ruling out compression and chemotaxis before demonstrating it is a coupling of EM cell polarization to ectoderm flow. The authors then develop a simple mathematical model for the coupling that reproduces important features of the experimental observations.

Overall, this is a well-written and very interesting paper with clear and careful experiments to justify its conclusions. In particular, the development and validation of a new explant system for studying EM cell migration, the clever quantitative analysis of cell shapes, trajectories, and motion correlations, and the optogenetic perturbations are all outstanding. The conclusion, that cell-on-cell crawling is cued by underlying tissue flows, is likely to be very high impact, and may drive other researchers to look for similar phenomena in their own systems.
There are a few issues we would like to ask to be clarified before publication. As our expertise in in modeling, we focus most of our questions on how the authors use modeling to understand the quantitative results.

Major concerns:
1. In Fig. 4 F i., the modeling results are plotted a separate panel from the experimental results. Is this for visual clarity or is there a reason they cannot be plotted together? For example, it is difficult to tell if the slopes are the same. Averages of the experimental data could be compared directly. If the slope of the model does not match, what is missing from the model that would adjust this? Qualitatively the trends are similar; the velocity increases with radial distance, up to the edge where it falls off, which does capture the qualitative trend in the experimental data. But if possible a direct quantitative comparison would be more convincing.

This is an insightful comment that prompted us to perform further analysis. Regarding the two separate plots, it was indeed our initial idea for clarity purposes. We thought that superimposing the 2 plots with multiple curves and points could make it a bit difficult to visualise, hence the decision to keep them separated. However, the reviewers are correct in pointing out that the slopes of the curves for our initial ectoderm model did not match exactly the slope of the experimental data, even though they both share a similar trend. The main reason for this discrepancy is that the original model assumed identical behaviour along the entire radius of the explant, with a y-intercept that by definition must be at y = 0, resulting in a steeper slope than observed in the data. In real explants, however, the central region forms a larger aggregate from which the ectodermal sheet spreads outward onto the substrate. We have now revised the model for the ectoderm to account for the role of the aggregate by including a boundary condition at the border of the central aggregate (R_a), where an additional influx (J) of cells from the central aggregate intercalate into the moving peripheral cell sheet (Figure 4K). This influx is a new ingredient that we added to the original active wetting model (Pérez-González et al., 2019) that we used as the basis for the model in our current paper. With this new ingredient, we were able to more closely match the slope of the model with the slope of the experimental data. We now show the direct comparison between theory and experiment in a single plot in Figure 4L, i.

2. In Fig. 4 F ii., the model proposes that the EM velocity profile is correlated with the ectoderm profile, up until a saturation point. However, the experimental data is only within the region where the model has well saturated. Without any evidence of the shorter distances, it doesn't seem appropriate to speculate that the much on this region of the profile. Even if there is a boundary condition that the curve must start at 0, it could very well be a sigmoid or any other function.

We agree with the reviewers that we cannot conclude about the model for small ectoderm speeds, corresponding to small distances from the tissue centre. As pointed out by the reviewers, the response of myeloid cells to ectoderm flow could be complex and captured by a function with a different form. However, our choice for the specific form in Eq. 3 is physically motivated based on linear response theory. The argument is as follows: The ectoderm flow is like an external field, and the polarization of the myeloid cell population is the response to that field. Thus, at small ectoderm speeds, we expect the myeloid cell population to respond linearly. Hence, we expect $v_\mathrm{m} \sim v_\mathrm{e}$ at small v_e. The simplest function that connects this linear response regime at small ectoderm speeds with the saturated regime at high ectoderm speeds is Eq. 3 in our paper. This is the reason we chose it. This function is a Hill function of order 1. We agree with the reviewers that, if the response of the cells to flow is more sudden for some reason, it could be a sigmoidal function, which could be captured by a Hill function of higher order. However, given that we cannot measure the movement of EM cells closer or at the centre of the explant, we made the parsimonious choice of the Hill function of order 1 to fit the data. In the revised manuscript, we explain this point right before Eq. 3. Investigating this response function is a very interesting direction for future work, which could provide insight into how cells orient their migration due to the flow of surrounding tissue. In line with the previous point, we have

also superimposed the model and the experimental data for the myeloid velocity in the revised Figure 4L, ii.

3. While the observational data in Fig 5 is compelling and interesting, it seems that the quantitative evidence is focused only on one or two cells. In other words, there is no statistical data averaged over (even a small) ensemble of cells, and so it remains unclear whether this reorientation always happens in response to an optogenetic perturbation, or whether these one or two cells that are analyzed are especially sensitive. While we recognize these are incredibly difficult experiments, is there any way to get a sense of statistics for the response to perturbation? E.g., if you analyzed 3 cells in the same way you analyze Fig 5f, would they look similar?

We thank the reviewers for this question. The montage shown in Figure 5F was a representative example, but the analysis of the angle change shown in Figure 5H is based on 18 cells, which is stated in the figure legend. As stated in the text, the average angle between the photoactivation centre point and the cell's major axis before photoactivation is around 60°. This is because the activation region is often to the side of the EM cells. After photoactivation, the average angle reduces to around 30°, and in many cases, becomes completely aligned to the photoactivation centre as seen in the rose plot at Frame 140 (Figure 5H, ii). As requested by the reviewer, we have now also added a box plot (Figure 5H, ii) for a statistical comparison between the angle of Frame 1 (before photoactivation) and Frame 140 (after photoactivation, where the cells are most aligned with the flow direction), showing that this reduction is statistically significant.

In addition, and to strengthen our *ex vivo* observations, we have performed optogenetic experiments *in vivo*. We added Figure 5I, J, and SuppFigure 4F as an additional line of evidence showing the same response EM cells have in the presence of a local contraction-induced tissue flow. We added a quantification for the protrusion/retraction area change, as well as angle change in Figure 5J, ii, iii. As can be seen from the graphs, only in the photoactivation condition (solid lines) do we see an increase in protrusion and retraction of the nearby EM cells, indicating the cell is moving toward the contracting area. This did not happen in control embryos where there is no contraction-induced tissue flow (dotted lines). We also added additional quantification to estimate the speed of the optogenetic-induced contraction in SuppFigure 4A, B, E, F to show that this contraction speed is similar to the natural movement of the ectoderm, both *ex vivo* and *in vivo*.

4. Is there any evidence that there might also be a direct mechanical interaction between the EM cells and the ectoderm, not just a signaling that induces EM polarization? For example, if EM cells are strongly coupled via friction or adhesion to the ectoderm, one might expect that the flow of the ectoderm would drag the EM cells along, not just change their polarization. This would add a contribution that correlates the directionality at T=0 in Fig 4D. Clearly, Fig. 4D shows that this effect, if it exists, is sub-dominant to a 5-min-delayed, polarization-based mechanism, but can it be ruled out as a contribution?

This is an important point raised by the reviewer. The proposed working model is that the movement of the local ectodermal tissue polarises EM cells via direct mechanical interaction, either through non-specific friction-like interaction or via a more specific cell-cell interaction. We did consider the involvement of a passive dragging effect and never entirely ruled out its contribution. However, we don't believe this to be a major driving force, considering that EM cells actively form protrusions in the direction of contraction, which would not be needed if the cells are just being dragged passively. And when we induce the contraction in the opposite direction, the EM cell reverses its polarity, which suggests that this is an active response, and not simply a dragging effect. Another point to consider is that the average speed of EM cells is around twice as fast as that of the ectodermal cells in explants (Figure 4B, iii versus SuppFigure 4, B, ii), which makes the contribution of any dragging effect minimal. However, we agree that it could still contribute given how intertwined the 2 cell populations are within our explant system. Working out the relative contributions of dragging vs flow-induced polarisation is an interesting question for future research.

We have added a discussion to the manuscript:

"Another question is whether passive dragging could explain collective-assisted directional migration. Our observations that the ectodermal flow induces an actin-rich protrusion in EM cells suggest that passive dragging is not dominant. Moreover, if passive dragging were a major contribution to EM cell dispersion, we would not expect to detect a 5-minute delay in EM cells' response. Nevertheless, teasing out the relative contributions of passive and active responses is an interesting question for future studies."

Minor concerns:

5. It might be helpful to provide a little more clarity about the geometry of the tissue and the plate in the explant experiment. To us, it was a little unclear whether -- when explants are placed on the plate -- the EM cells crawling along the plate, or through the ectoderm. The cartoon of the embryo in Fig. 1 A makes the ectoderm look like a top layer with EM cells within the mesoderm, but the cartoon in Fig. 4 of the explant on the dish looks like the EM cells are just within the ectoderm.

We apologise for the confusion concerning the geometry of the explants, which we have now improved by adding extra schematics to further clarify the geometry of our explant system in Figure 1A and B. The cartoon in the now Figure 4K has been updated accordingly. In the explant, EM cells are originally a part of the mesoderm (the precursor cells) that sits at the centre of the explant (the bulging part at the centre). From here, EM cells escape into the sheet of spreading ectodermal cells. This sheet is also composed of more than one layer of ectodermal cells, which means EM cells are not moving in a flat 2D surface but in a 3D environment that allows them to move in all 3 dimensions: x, y, and z (SuppFigure 1C and SuppVideo 2). EM cells therefore can interact with the fibronectin-coated substrate underneath as well as surround themselves with other ectodermal cells, depending on where they are, which is similar to what they would experience when moving through the ectoderm in an embryo. Our explant system can

faithfully recapitulate this EM cell-ectodermal cell interaction.

6. If the EM cells are crawling within the ectoderm, when the ectoderm is removed, is part of the change due to cells crawling on just the dish compared to crawling inside a tissue?

The reviewer is correct in noting that, upon removal of the ectoderm, the cells alter their shape and migratory parameters as a result of migrating on just the dish rather than within a three-dimensional ectodermal tissue. Within the ectodermal tissue, EM cells can make contact with the surrounding ectodermal cells and also with the dish as they are moving in a 3D environment. We have now added another schematic panel in Figure 1B, ii, to clarify the geometry of EM cells within the explant.

Reviewer #2 (Remarks to the Author):

I co-reviewed this manuscript with one of the reviewers who provided the listed reports. This is part of the Nature Cell Biology initiative to facilitate training in peer review and to provide appropriate recognition for Early Career Researchers who co-review manuscripts.

Reviewer #3:

This manuscript investigates how primitive embryonic myeloid (EM) cells disperse during early Xenopus development. The authors proposed that EM cell migration is guided by ectodermal tissue flow, a result of the overall embryo elongation. The authors used both frog embryology approaches and newly developed explant culture assays to characterize cell movement and concluded that local ectodermal tissue flow can reorient EM cell protrusions and bias their migration direction. The study also noted that EM cells can switch between more lamellipodial and amoeboid morphologies, though this aspect is only briefly discussed.

Overall, this study addresses an important, underexplored, and underappreciated form of cell movement, the cell-on-cell migration, rather than cell-on-ECM migration, and provides a valuable framework for understanding how collective tissue dynamics can influence other cells around them.
However, I find that the central conclusions regarding tissue flow require additional supporting analysis and clarification of some ambiguities in the experimental setup and data interpretation.
While the discussion section does a good job of acknowledging alternative interpretations, the results as presented are not yet described clearly enough or supported by sufficient quantitative evidence. I therefore recommend a major revision.

Three major concerns:

1. Lack of baseline correlation between ectodermal tissue flow and myeloid cell migration.

The central claim of this study is that EM cell dispersion is guided by ectodermal tissue flow during embryo elongation. However, the current manuscript does not clearly establish this relationship in unperturbed wild-type embryos. Demonstrating a baseline correlation between ectodermal flow and EM cell trajectories under normal conditions could significantly strengthen the conclusion and seems to be straightforward to do.

While Figure 7E suggests a slight indication of this relationship, it was not quantified or analyzed systematically. Moreover, the accompanying Video 23 shows a burst of EM cells migrating towards the anterior-dorsal/lower-left after the tissue flow slowed down. The authors should perform an embryo-wide correlation analysis between the ectodermal flow and EM migration in control embryos to establish this foundational relationship. The same analysis could be performed for all in vivo perturbations, similar to what has been performed in the explants.

We thank the reviewer for this important comment, which has prompted us to perform additional *in vivo* experiments and to apply the same correlation analysis pipeline used for our *ex vivo* model, finding that EM cell directionality but not speed shows a time-delayed correlation with ectoderm flow, consistent with our *ex vivo* results. We have added the following paragraph with the corresponding new figures and videos:

"*To check whether these correlations are also present in vivo, we live-imaged wildtype embryos with labelled ectodermal nuclei and grafted fluorescent dextran-labelled aVBI to ensure the best signal for tracking. We applied the same analysis pipeline used in our ex vivo data and found no correlation between the speed components of EM cells and the surrounding ectodermal cells (SuppFigure 3M, i, SuppVideo 21), consistent with the previous result (SuppFigure 3I). However, more importantly, we detected a statistically significant correlation between the angle of migration of EM cells and the ectodermal cells in vivo (SuppFigure 3M, ii), along with the 5-minute peak shift as detected in our ex vivo data (SuppFigure 3K). These observations show that ectodermal tissue flow can influence EM cells' directionality, but not speed, both ex vivo and in vivo.*"

In the constrained embryo experiment, the main message is that when embryo elongation stops, so does EM cell migration, and this has been consistent across our experiments (Figure 7B). But because the ectoderm in these embryos does not move, it is not possible to perform the same correlation analysis since the analysis pipeline relies on the movement of both cell populations to infer a correlation coefficient.

We also would like to further clarify that we do not propose tissue flow to be the main force that drives EM cell dispersion, but a new guidance cue for EM cells. In the absence of chemotaxis, tissue flow appears to be able to bias EM cells' protrusion dynamics and directionality, as seen in our *ex vivo* experiments. *In vivo*, tissue flow likely acts in concert with other guidance cues to control EM migration.

Additionally, given that ectodermal flow is likely spatially complex in both control and perturbed embryos, imposing a more defined flow could also be informative. For

example, mechanically stretching embryos or explants along the head-to-tail axis, or orthogonally, might help create a clearer flow field and provide an additional layer of evidence supporting the authors' conclusion.

We thank the reviewer for this important point, which prompted us to explore several mechanical approaches to stretch embryos and *ex vivo* explants. However, this quickly proved to be a non-trivial engineering challenge. For a mechanical device to function as intended, the applied stretching must be sustained over time, requiring continuous deformation of the embryo, akin to its natural growth. Without sustained stretching, the induced tissue flow is only transient. In the case of explants, mechanical stretching often led to their detachment or disintegration. We also attempted to stretch embryos by aspirating them into a capillary but encountered the same limitation of maintaining a sustained stretching over a long period of time. However, to address this important issue raised by the reviewer, we performed *in vivo* optogenetics, which allows us to control the flow of tissue more locally with higher precision and to visualise its effect immediately.

A detailed description of the *in vivo* optogenetics is in the Materials and Methods section as follows:

"For in vivo optogenetics, we injected 20 pg of the Opto-IP mRNAs into all 4 animal blastomeres. For each embryo, we grafted a Ruby-LifeAct-labelled aVBI explant from a donor embryo to ensure no cross-contamination. Embryos were left to develop to around Stage 26 to 28, when EM cells have migrated out from the aVBI. We then laid the embryos onto their side and mounted them in 0.8% low melting agarose dissolved in 0.1X MMR mixed with Tricane (0.24 mg/ml) in a glass-bottom dish. A square glass coverslip was quickly placed on top and gently pressed down. To perform the optogenetic induction, a region next to the target EM cell was activated. A z-interval of 1 µm was used to ensure EM cells in the deeper tissue layer were captured. Imaging conditions are as follows: Zeiss880 confocal mode, X40 OIL lens, wavelength 454 nm, 10% laser power, consecutive interval, started at Frame 2, and repeated every frame until the end. Both the ectoderm and the EM cells were imaged with a 568 nm wavelength, 5% laser power, and a digital gain of 700."

We explained the result in Figure 5J, and SuppFigure 4E, F, and added the following section to the main text:

"Finally, we asked whether the optogenetic response observed ex vivo was also conserved in vivo. We first expressed the Opto-IP system in the entire ectoderm layer of acceptor embryos before grafting aVBI tissue containing Ruby-LifeAct-labelled EM cells from donor embryos lacking the Opto-IP system. We left the embryos to heal and grow to around stage 25 to 27 to ensure that some EM cells have had the time to spread into the ectoderm before imaging (Figure 5I). Upon photoactivation, the ectoderm visibly contracts at an average speed of 0.6 µm/min, which is also in line with the speed of a naturally moving ectoderm in wildtype embryos (Figure 5J, ia, ib, SuppFigure 4E, i, ii). This contraction biased a pre-existing protrusion and simultaneously induced the formation of a new protrusion toward the contracting ectoderm in the neighbouring EM cell (Figure 5J, ic, SuppVideo 26). Importantly, this response was not observed in the control condition, where the Opto-IP constructs were

not expressed and therefore, no ectodermal contraction occurred upon blue light activation (SuppFigure 4F, SuppVideo 27). Measuring the area of protrusion extension and retraction of EM cells over time shows no change in both parameters in the control condition (Figure 5J, ii, dotted lines), while significant increases over time were observed in the photoactivated condition (Figure 5J, ii, solid lines). The angle of alignment α between EM cells' protrusion and the centre of the photoactivation region was similarly decreased from around 80° to around 30° (Figure 5J, iii, green line), while no change was observed in the control embryos (Figure 5J, iii, blue line)."

The above result suggests that local tissue flow can bias neighbouring EM cells' protrusion dynamics and migration, which is in complete agreement with our *ex vivo* data.

2. Unclear explant setup and its correspondence to in vivo EM cell positioning

I feel it is difficult to visualize how the described explant recapitulates the in vivo arrangement of ectoderm, mesoderm, and EM cells. In the explant setup, it seems that the authors isolated ectoderm and mesoderm together and cultured them on a fibronectin-coated dish, with the mesoderm placed downward and the ectoderm on top. However, it is unclear to me why in Fig. 4E the ectoderm forms a bump in the center, the mesoderm layer was excluded, and the EM cells were dispersed within the ectoderm.

Additionally, according to Fig 1A and SFig 1D, in vivo EM cells first migrate underneath the ectoderm within the mesoderm, and then penetrate deeper into the endoderm. However, within the explant, the ectodermal layer appears to spread rapidly onto the fibronectin-coated substrate, retaining the mesodermal cells at the center. Thus, upon the onset of EM cell migration, they entered the ectoderm directly (SFig. 1C), rather than migrating within the mesoderm sandwiched by two fibronectin layers.

This setup is confusing as presented. The authors should provide a clearer schematic and text description of the explant configuration, and more importantly, discuss how this arrangement reproduces the in vivo environment. If the difference is apparently substantial, the authors should also clarify which aspects of the ex vivo behavior can be meaningfully translated to the in vivo situation.

We thank the reviewer for this point, and we agree that our previous description of the *ex vivo* setup was confusing. To address this problem, we have added extra schematics in Figure 1A and 1B to further clarify the geometry of the explant. The reviewer is correct in pointing out one of the differences between the *ex vivo* setup and *in vivo*. *In vivo*, EM cells can migrate in all 3 germ layers and everywhere in between, but in our *ex vivo* setup, the anatomical aspect that is preserved the most is the interaction between EM cells, ectodermal cells, and the fibronectin layer underneath. In the *ex vivo* setup, the mesoderm is less motile than the ectoderm, so it remains at the centre of the explant. The ectoderm spreads outward on top of the mesoderm and then onto the fibronectin-coated substrate. When EM cells leave the mesoderm, they will be in contact with the

spreading ectoderm and the fibronectin underneath, which is similar to an aspect of their migration *in vivo*. We highlighted this similarity specifically in the zoom-in schematic in Figure 1A and Figure 1B, ii. We have modified the following texts to reflect these changes:

In the introduction:

"Early Xenopus embryos lack a functional circulatory system, similar to zebrafish[17], so dispersing EM cells can migrate within and underneath ectodermal cells, in between mesodermal cells, and later, invade into the deeper endodermal tissue[24–26] (Figure 1A)."

"To dissect the mechanism of how EM cells navigate through dense tissue, we first take advantage of the easy-to-culture Xenopus embryo tissue to develop an ex vivo culturing system that preserves some aspects of the natural tissue anatomy and the developmental dynamics of the EM cells while also being highly reproducible and easy to set up.";

and in the first results section:

"In our ex vivo model, the EM precursor cells (Figure 1B, ii, pink cluster), which are a part of the mesodermal tissue, are less motile than the ectodermal layer, and thus remain largely at the centre of the explant. When EM cells emerge from this mesodermal tissue, they enter the environment of a moving ectodermal layer. These observations suggest that our explant system faithfully recapitulates the natural development of EM cells and that it serves as a suitable model for studying the interactions between EM cells and ectodermal cells as found in vivo."

3. Clarification of fibronectin distribution and its role in EM cell migration in vivo

In the Introduction, the authors state that "Only a thin layer of fibronectin separates the ectoderm from the mesoderm, and the mesoderm from the endoderm30–32, making EM cell dispersion primarily a cell-on-cell migration process". However, this statement appears inconsistent with SFig. 1D and published data as in Davidson et al., Dev Dyn, 2004.

The aforementioned fibronectin layers appear reasonably dense. Because fibronectin fibrils can be stretched and remodeled by cell contractility and the fibronectin network can be patterned by tissue-scale cell flows, the ectodermal cell movements described in this work may similarly organize the fibronectin network. I am prone to believe that the ectodermal flow guides EM cell migration partially through fibronectin.

I recommend that the authors reassess this aspect through both a literature review and experiments to clarify the potential role of fibronectin.

We thank the reviewer for raising these interesting points. To clarify, when we said, "thin layer of fibronectin", this was meant to be compared with the size of the surrounding cells. Within an embryo, EM cells can interact with the fibronectin layer, but this layer is

most often bundled and confined to between the germ layers rather than infiltrating extensively between cells within the ectoderm. These separating and confined layers of fibronectin can be seen very nicely in (Davidson et al., 2004, 2006). This means when EM cells migrate through the ectoderm, they will most often interact with the surrounding ectodermal cells, which is what our paper is focusing on. We have now adjusted our wording as follows:

"*Cross-section images of later stage embryos show confined bundles of fibronectin separating the ectoderm from the mesoderm, and the mesoderm from the endoderm[33–35] rather than interdigitating between cells of the ectoderm, suggesting that EM cell dispersion through tissue is primarily a cell-on-cell migration process."*

Regarding the idea of fibronectin remodelling, it is indeed very interesting and entirely possible that the ectodermal tissue or mesodermal tissue could modify the fibronectin matrix to regulate EM cell migration in embryos. However, we have now added experimental results that found no direct contribution of fibronectin to EM cell migration *in vivo* in early embryos by using a translation-blocking morpholino against fibronectin. This finding may not be that surprising, as multiple studies have shown that immune cells can still migrate normally even without any integrins or adhesion molecules just by using the physical topography instead of the chemical composition of the environment (Lämmermann et al., 2008; Reversat et al., 2020). We also found no evidence of fibronectin remodelling by the ectoderm *ex vivo* for both endogenous and exogenous fibronectin using fixed immunofluorescence or live cell imaging. It is important to note that the ectoderm does not produce fibronectin; rather, ectoderm explants adhere to and spread on exogenous plasma fibronectin. This form is inherently more difficult to assemble into fibres than cellular fibronectin, largely because it exists in a compact conformation (Baneyx & Vogel, 1999). Therefore, it is unlikely that the ectoderm can remodel fibronectin fibres, consistent with our new observations. These data are discussed in detail in the added text:

"One hypothesis for how the ectoderm flow could influence EM cell migration is through the remodelling of fibronectin. To first establish whether there is a direct role of fibronectin in EM cell migration, we depleted the expression of fibronectin in one half of each embryo using a morpholino and compared the number of EM cells that migrated into the control and the injected sides (Figure 4C, i). Embryos bent towards the injected side, similar to those injected with fibrillin morpholinos[35,72] (Figure 4C, ii). However, pair-wise comparison between the two sides shows no statistically significant difference in the number of single EM cells (Figure 4D), showing that fibronectin is not required for in vivo EM cell dispersion. This is consistent with previous studies showing immune cells that can migrate even in the absence of focal adhesions[73,74]. We confirmed the efficacy of the knockdown using immunofluorescence (SuppFigure 3F, i, ii). Although these observations indicate that EM dispersion is unlikely to require fibronectin in vivo, we nevertheless examined fibronectin matrix remodelling by the ectoderm ex vivo. We plated aVBI explants on a fibronectin-coated surface, and then fixed and stained with a Xenopus-specific anti-fibronectin antibody to assess whether the ectoderm could remodel endogenous fibronectin (Figure 4E, i). We found that the endogenous fibronectin was located exclusively within the mesodermal layer

and, therefore, there was virtually no overlap between EM cells and the fibronectin signal in the ectoderm region (Figure 4E, ii). We then stained the explants with a polyclonal antibody that recognises the exogenous fibronectin used for coating (SuppFigure 3G). The entire surface was homogenously stained except for a few fibronectin fibres located exclusively in the mesoderm, which has already been described as a site of fibronectin remodeling[75], with no fibres detected within the ectoderm region (SuppFigure 3G). Quantification of the percentage of EM cells colocalising with the anti-fibronectin signal shows that most of them do not colocalise with any fibronectin (Figure 4E, ii). We then performed live cell imaging using a fluorescently labelled version of the Xenopus-specific fibronectin monoclonal antibody. The antibody showed a strong signal for fibrous fibronectin within the mesoderm region of the explants, similar to what was observed in the fixed samples. Labelled EM cells were seen interacting with the fibronectin fibres, but once they left the mesoderm, no fibres were seen associating with these cells (Figure 4F, i, SuppVideo 18). Quantifying the amount of fibronectin within the mesoderm versus the ectoderm shows that virtually all the fibronectin fibres remain within the mesoderm, even as time progresses, and the explants expand (Figure 4F, ii). We then plated aVBI explants onto a Rhodamine-fibronectin-coated surface to look for signs of exogenous fibronectin remodelling (Figure 4G). Most explants showed no sign of fibronectin remodelling apart from a small portion of explants that had some fibrous structures (Figure 4G, H, white arrow, SuppVideo 19). However, these fibres were rare and confined to the mesoderm, with little to no interaction with the EM cells. If the ex vivo radial migration of EM cells were to be explained by the ectoderm remodelling of fibronectin, we would have expected to observe a more extensive network of fibres associated with the EM cells, particularly in the ectoderm region. However, this was not the case in all the experiments we performed. Therefore, we conclude that fibronectin does not play a major role in EM cells' migration in vivo, and that ex vivo, the ectoderm layer does not exhibit any apparent fibronectin remodelling during EM cells' migration."

Minor concerns:

1. The transition between more lamellipodial and amoeboid morphologies of the myeloid cells is very interesting. Could the authors guess which aspects of ectodermal tissue dynamics might influence this transition?

This is one of the very interesting initial observations. We think that part of the mechanism that regulates the shape and migration mode of EM cells has to do with the natural confinement imposed by the ectodermal cells. When EM cells are inside the ectoderm, the ectodermal layer can compress them, giving them an elongated morphology and triggering a transition to amoeboid motility (Liu et al., 2015; Ruprecht et al., 2015), characterised by a rearward positioning of myosin, which was partially replicated when we artificially compressed the cells with a coverslip (Figure 3B, F). However, there are other mechanisms that might also contribute, such as the presence of bound signalling molecules on the surface of ectodermal cells, or direct cell-cell interactions, similar to how border cells polarise within the Drosophila egg chamber by forming E-cadherin interactions with the surrounding nurse cells (Cai et al., 2014). None of these effects would be present outside of the ectoderm.

2. The optogenetics data seem very promising. When looking at Video 19, an apparent ectodermal cell flow was triggered, and the EM cell below was repolarized very quickly. However, I also noticed that other EM cells around the activation spot tended to respond differently. For example, the right EM cell moved away and against the flow, and a few cells above that that formed doublets remained static. Could this be distance, flow speed, or morphology-dependent? How might this heterogeneity affect EM dispersion in vivo?

We thank the reviewer for this astute observation. The degree of response can be quite varied depending on many factors, such as how far the cells are from the contraction area, how strong the contraction is, what shape the EM cells have at that moment (because this can affect cortical tension, and therefore protrusion formation), whether there are other EM cells around (labelled or unlabelled), all of which is difficult to control for experimentally, as we had to rely on finding locations where there were both enough Opto-IP expression in the ectoderm and also at least one nearby EM cell. In the video mentioned by the reviewer (now SuppVideo 25 instead of Video19), the cell that moves away seems to already be at the edge of the explant, and so when the explant contracts, the cell gets expelled, as it will not be under the ectoderm influence anymore. The contraction-induced flow might not be strong enough for this cell or that this particular cell might adhere a bit more strongly to the substrate, as these EM cells can be heterogenous in their sensitivity, which is something we did not specifically focus on in this manuscript, but we do appreciate that such heterogeneity exists. Nevertheless, in most cases, a nearby contraction-induced flow is able to produce a response in nearby EM cells as quantified in Figure 5H.
For the doublet cells, they did in fact respond to the contraction by transiently forming a protrusion and moving closer at the end of the movie. However, it seems their rounder shape might have prevented a more prominent actin protrusion from forming, likely because of higher cortical tension. We have also added a statistical analysis of multiple EM cells (Figure 5H, ii) to show that photoactivation yielded a significant decrease in the angle between the cell protrusion and the photoactivation spot. We also reconfirmed these observations with *in vivo* optogenetic experiments in Figure 5I and J, SuppFigure 4E and F, as mentioned in an earlier response. These results show a consistent response of EM cells to the local contraction-induced tissue flow.

We expect that this level of variability in sensitivity of EM cells can be higher *in vivo* simply due to the presence of other guidance cues, which don't necessarily exist in our *ex vivo* system. Therefore, EM cells *in vivo* will likely have to integrate many more different guiding signals to make a decision. Being able to tease out the individual contribution of each factor would be very helpful but is beyond the scope of our current work. But even then, we were still able to induce protrusion biases of EM cells using just local tissue flow induction and we were able to detect a correlation in their migration directionality with the ectoderm *in vivo*, which suggests that this phenomenon does exist in embryos.

3. Fig. 1B does not seem to be at the 32-cell stage, but a little later compared with Ref 33. Does this timing provide a better labeling of EM cells?
We thank the reviewer for spotting this. We have replaced it with the correct hand-drawn cartoon of the 32-cell stage embryo.

Reviewer #4:

The manuscript by Anh Le et al. investigates how tissue movements influence single cell migration. Specifically, they explore the mechanism by which embryonic myeloid (EM) cells migrate during the early stages of development. The authors employ an ex vivo culture system using tissue explants from Xenopus laevis to show that EM cells move less directionally in the absence of the ectoderm. The ectodermal layer also contributed to polarity of the EM cells, as measured by the localisation of cytoskeletal markers. Physical compression or response to chemotactic cues were not identified as the reason for the change in the polarity and migration of EM cells that were cultured without the ectoderm. Optogenetic manipulation of ectodermal tissue flows perturbed the migration of EM cells, indicating that ectodermal tissue movement play a role. Furthermore, genetic disruption of ectodermal tissue movement and mechanical restriction of tissue elongation reduced the dispersal of EM cells. Regarding novelty, other mechanisms have been described that help single cells spread in multicellular tissues, but the notion that tissue movements play a role has been less explored. The paper uses elegant approaches like optogenetics and mechanical manipulations to causally link tissue movements with the behaviour of EM cells. The findings are interesting and novel, but the mechanisms involved could be further clarified, to understand whether the effects are purely mechanical in nature or act via chemical pathways.

Major points

-An important point to address is the possibility that tissue flow influences EM movement via chemical signalling versus direct mechanical signalling. For example, ectodermal cell flow could change the distribution pattern of a chemical ligand (produced by ectoderm or EM cells) on the ectoderm surface. In terms of addressing the possibility of chemotaxis, the authors attempted one pathway but there are more general perturbations that can be tested. For example, inhibition by pertussis toxin could be used to block Gai signalling, without needing to identify the specific ligand/receptor. In addition, supernatants from ectoderm can be collected, concentrated and then added to the EM cells. The authors could also consider candidates from the literature for which there is evidence for a role in EM spreading. In zebrafish, m-csf plays a key role in macrophage early invasion of different tissues. https://doi.org/10.1006/dbio.2001.0393. It could be that tissue flows influence distribution of such ligands.

We thank the reviewers for this comment as it led us to perform several additional experiments to address it. However, it is important to note that we never entirely exclude the role of chemical signalling in guiding EM cells, especially *in vivo*. Instead, our report

is attempting to show that mechanical signals also play an important role. Certainly, we believe that both mechanical and chemical pathways will play an intertwined role in EM dispersion. One could speculate that the mechanical movement of the ectoderm may change the distribution of chemokines, and that, then, causes a response in EM cells. However, it is not clear how the spreading of the ectoderm could generate a central-to-peripheral gradient of chemoattractant within an explant. If the ectoderm were simply carrying the chemoattractant along, it would more likely do the opposite, as the ectoderm could dilute the chemoattractant as it spreads. In addition, the response time of EM cells to an optogenetic-induced contraction was within seconds, which is much faster than chemical responses, which can take minutes (Zhang et al., 2009), suggesting that mechanical factors are at work.

In terms of the suggested conditioned media experiment, we believe this is more appropriate for *in vitro* cell culture, where the cell number can be easily controlled, and thousands of cells can be cultured cleanly. This is, unfortunately, not the case when working with an *ex vivo* explant system. The system is essentially a co-culture of both ectoderm, mesoderm, and EM cells, and it is not possible to cleanly separate the ectoderm layer from the other tissue, so it is not possible to be certain that the conditioned media would only contain secreted factors from one tissue layer. In addition, as the explants are manually dissected from individual embryos, it is not feasible to accurately control for cell number variations or to dissect enough ectodermal explants for conditioned media collection.

Having said that, and following the suggestion by the reviewer, we have done experiments where explants were treated with pertussis toxin (PTX) or anti-CSF1R antibody to inhibit CSF1R signalling. However, these manipulations have very limited impact on EM cell behaviours (SuppFigure 2F, K). Combining these new observations with the other experiments that we have done, we think that soluble chemoattractants are likely not to play a big role in regulating EM cells migration, at least *ex vivo*. We added the following paragraphs to our main text:

"Another major chemotactic molecule for macrophages is the macrophage colony-stimulating factor (M-CSF or CSF1), which is important for the dispersion of early macrophages in the brain in zebrafish[56]. However, treatment with an anti-CSF1 receptor antibody[57,58] only resulted in a marginal reduction in the overall speed with no significant change in the persistence index of EM cells (SuppFigure 2F, i, ii, SuppVideo 10). This suggests that CSF1 is unlikely to contribute significantly to the observed directional migration of EM cells ex vivo."

"General inhibition of GPCR signalling, which is another major chemotaxis pathway[63], by pertussis toxins (PTX) only marginally reduced EM cells' mean speed but did not change their persistence index (SuppFigure 2K, i, ii, SuppVideo 12). These experiments suggest that soluble chemoattractants are likely not the main players controlling the radial dispersion of EM cells ex vivo."

-In relation to the above, the experiments with liquid flushing are informative for soluble

ligands, but do not exclude chemoattractant ligands (e.g. chemokines) that can immobilise on the surface of the ectoderm. Heparan sulfate is a common cell surface sugar on which chemokines can immobilise and can be inhibited by heparinase treatment.

We thank the reviewers for raising this important point. We did briefly mention the possibility of bound chemokines in our discussion, and we certainly still think this is a possibility. In fact, we think that this could be one of the ways in which EM cells locally interact with the ectodermal cells since the glycosylated membrane proteins are important for cell-cell interactions (such as during neutrophil extravasation, where they interact with the endothelial layer, something that we also mentioned in our discussion). Following the reviewer's advice, we treated our explants with heparanase (SuppFigure 2K) and saw that this significantly impaired EM cell migration, which is a very interesting observation. This result could hint at the presence of bound chemokines on the surface of the ectoderm or that glycosylation is important to EM cells' migration. We would argue that either of these effects could be related to the mechanisms underlying our discovery of ectoderm flow acting as a guidance cue for EM cell dispersion. One could speculate that the movement of the ectoderm might pattern bound chemokines to allow EM cells to follow, or that a surface cell-cell adhesion (such as syndecans or integrins) modulated by glycosylation could mediate the interactions between EM cells and the ectoderm.

We added this paragraph to the main text:

"*We next wondered whether a more direct local interaction between EM cells and ectodermal cells would be more relevant. To this end, we treated our explants with heparanase, an enzyme that digests the sugar heparan sulfate attached to many transmembrane proteins, which mediates cell-cell interactions and haptotatic signalling*[64–67]*. Indeed, EM cells in heparanase-treated explants have a significantly slower mean speed and persistence index compared to the untreated explants (SuppFigure 2K, i, ii, SuppVideo 12). Together, these data argued against soluble molecule-driven chemotaxis being the main guidance cue for the radial migration of native EM cells ex vivo, while suggesting that direct local interactions between EM cells and ectodermal cells may be more important.*"

And to the discussion:

"*One of the interesting observations is the decrease in both the migration speed and directionality of EM cells when explants were treated with heparanase. Heparanase digests the sugar chain heparan sulfate, which is a typical post-translational modification for cell surface proteins*[111,112]*. Heparan sulfate has many different roles, including regulating protein functions*[113]*, protein-protein interactions through binding to and patterning of morphogens*[114,66]*, and cell-cell interactions such as during transendothelial migration*[64,67]*. EM cells seem to respond to the loss of heparan sulfate, which could be an indication of the existence of a haptotactic gradient of an unknown molecule bound to the surface of ectodermal cells. It is also possible that the loss of heparan sulfate on the surface of EM cells or ectodermal cells directly affects their cellular activities or cell-cell interactions through*

disrupting the functions of glycosylated transmembrane receptors such as syndecan[113]. Combined with the fact that EM cells' migration was impaired when the ectoderm was physically disrupted both ex vivo and in vivo, this result supports the hypothesis that direct physical interactions between EM cells and surrounding ectodermal cells are more relevant to their migration in our system."

Although we found that this result is very promising, it is beyond the scope of our current paper. However, this will be worth a follow-up investigation.

Minor points

-In the introduction, it would be helpful to summarise other ways of single cell spreading, e.g. through contact inhibition of locomotion (doi: 10.1016/j.cell.2015.02.015), dispersion through self-generated gradients (https://doi.org/10.1371/journal.pbio.1001966) etc. This would allow the reader to relate the findings to other mechanisms and better appreciate the novel aspect of tissue flow as a factor.

We have referenced the contact-inhibition papers in our discussion here:
"The current working model is summarised in Figure 8. EM cells start off as a cluster of mesenchymal cells at the aVBI. As the embryo develops, EM cells leave the cluster, perhaps in part driven by contact inhibition of locomotion[105,106]."

We have also now added a brief description of cancer metastasis to our introduction:
"Traditionally, the mechanisms governing single-cell migration through a tissue have been frequently explored in the context of cancer. In vitro studies show that single cancer cells can use self-generated gradients as guidance cues, such as a chemical gradient of LPA in the case of melanoma[30] and pancreatic cancer[31], or a stiffness gradient[32]. However, the mechanisms that guide single immune cell dispersion, such as within a developing embryo, remain largely unexplored."

-It is important to mention more studies that investigate cell-on-cell migration to better distinguish the advance made here and indicate the broader relevance of the findings. One example of relevance is the migration of immune cells on fibroblast networks within lymphoid organs (https://doi.org/10.1016/j.immuni.2010.04.017, and 10.1016/j.immuni.2006.10.011). Here leukocytes use stable fibroblast network for contact guidance. Interestingly, immobilised chemokines on the fibroblasts play a role in guiding these migration patterns. The authors could highlight the novel aspect of tissue movement as a factor and that discuss whether chemical ligands could also be influenced.

We thank the reviewers for suggesting these papers. We think these papers indeed add extra support to the idea that cell-on-cell migration is a widespread phenomenon. We have added and modified our discussion to incorporate these points:

"Looking beyond the context of development, cell-on-cell directional migration can also be present in other scenarios, such as during wound healing. Since mechanical tension could be transmitted through matrix fibres to attract distant macrophages[124], a similar mechanism could be at play near the wound edge, where the initial damage chemicals attract distant immune cells, while mechanical tension propagating through a network of connected cells could act like a "honing-in" signal to the exact site of injury. During cancer invasion, cells can collectively move in streams to metastasise. An analysis of their motion showed that the movement of these cells can align with their neighbours like a nematically active fluid[7]. In the lymph node, dendritic cells[133] , T cells, and B cells[134] have been shown to rely on either surface-bound chemokines or the architecture of stromal cells as guidance for their migration. These examples, therefore, suggest that our findings that tissue flow can guide cell-on-cell migration might apply to a broader range of biological contexts, from development to immunobiology to cancer biology."

- 'Early Xenopus embryos lack a functional circulatory system, so EM cells migrate beneath the outer ectodermal layer, between mesodermal cells, and later invade into the deeper endodermal tissue'. This phrase suggests that this circulation-independent dispersion pattern is specific to Xenopus. Yet EM cells in other organisms also disperse prior to circulation onset https://doi.org/10.1242/dev.126.17.3735. In addition, other models exist where cells spread without circulation. It would be helpful to emphasise more the broader relevance on the phenomenon rather than restrict the relevance to Xenopus.

The reason that we mentioned "*Early Xenopus embryos lack a functional circulatory system*" is only to set the stage as to why *Xenopus* embryos is a suitable model system to study cell-on-cell migration, along with their ability to easily setup *ex vivo* culture systems, which not many other animal models can do. We agree with the reviewer that there are other model organisms that also lack the circulatory system, which may suggest that cell-on-cell migration is indeed widespread. We have added more discussion to show the broader relevance of our phenomenon and our findings:

"Although our experiments were performed in Xenopus embryos, a similar challenge arises in the dispersion of embryonic macrophages in other systems. This includes the migration of equivalent cells from the anterior lateral plate mesoderm (ALPM) in zebrafish and from the yolk sac in mammals[24–26]. In all these cases, early macrophages may employ a cell-on-cell migration mechanism, as we have shown in Xenopus."

"While cell-on-cell migration is a widespread phenomenon that has been observed in different model organisms, including Drosophila melanogaster, zebrafish, and Xenopus laevis, the exact molecular mechanism for our collective-assisted directional migration remains elusive. In the previously described scenarios, cadherin-mediated cell-cell contacts and focal adhesions played a significant role in guiding cells along each other[8,9]."

"Looking beyond the context of development, cell-on-cell directional migration can also be present in other scenarios, such as during wound healing. Since mechanical tension could be transmitted through matrix fibres to attract distant macrophages[124], a similar mechanism could be at play near the wound edge, where the initial damage chemicals attract distant

immune cells, while mechanical tension propagating through a network of connected cells could act like a "honing-in" signal to the exact site of injury. During cancer invasion, cells can collectively move in streams to metastasise. An analysis of their motion showed that the movement of these cells can align with their neighbours like a nematically active fluid[7]. In the lymph node, dendritic cells[133] , T cells, and B cells[134] have been shown to rely on either surface-bound chemokines or the architecture of stromal cells as guidance for their migration. These examples, therefore, suggest that our findings that tissue flow can guide cell-on-cell migration might apply to a broader range of biological contexts, from development to immunobiology to cancer biology."

-The figures are generally well-presented, and the information is mostly sufficient. In Fig. 2 some informatio is missing on the number of embryos used for panel c and the number of cells and embryos in panel d.

We thank the reviewers for this point. In Figure 2C, we quantified based on the individual cells, not embryos, since these behaviours are single cell behaviour. Furthermore, when we release the EM cells from the fragmented explants, it becomes trickier to know from which explant/embryo these cells are coming, so reporting the number of embryos here is not possible.

In Figure 2D, this is an image montage to show the transition that we have been observing in previous experiments, where EM cells immediately become rounder after leaving the ectoderm (Figure 1C, Figure 4A), but now at a higher resolution. There is no quantification done here, so there is no report on the number of cells or embryos.

-In Fig. 3 the authors should ideally include the intact – compressed condition and fragmented-non compressed condition to facilitate interpretation of the datasets.

We thank the reviewers for this point, but we would argue that these two conditions are not required. The idea of the experiment in Figure 3 is to compare the morphology of EM cells being artificially compressed versus EM cells being naturally compressed by the ectoderm. So, in a way, we could view the "intact + uncompressed" condition as being naturally compressed. If we introduce another artificial compression on top of this, we will have two sources of compression: the compression coming from the ectoderm itself, and the compression coming from the external coverslip. So, this comparison would make the interpretation of the result more cumbersome, as we will be imposing two types of compression onto our explants simultaneously.

The fragmented-non compressed condition is already presented in Figure 3B to compare between artificially compressed vs non-compressed fragmented explants. It is not relevant to Figure 3F.
So overall, we believe our original experimental design is sufficient to address the question being asked.

-Fig. 5 shows optogenetic experiments that are important for mechanistic

understanding. Some quantification for imaging related to f and i is needed to support the claims. In addition, the authors can include controls showing the effect of photoactivation in explants not expressing the ontogenetic construct. The optogenetic activation seems of small scale, i.e. not enough to recapitulate the tissue scale flows. Is it possible to do the optogenetic activation in multiple points/cells to achieve bigger scale and potentially clearer effects?

We thank the reviewers for this point. We have now added a box plot quantifying the angle before and after photoactivation in Figure 5H, ii.

The optogenetic experiment was not supposed to recapitulate the entire ectodermal tissue scale movement, but rather to assess how local tissue flows affect EM cell polarisation. We have added extra quantifications on the contraction speed to show that the optogenetic-induced contraction speed is similar to the average ectoderm speed in SuppFigure 4A and B. Our optogenetic system is also constrained by the number of cells that express the constructs, since expression in embryos can be heterogeneous, so it is not always possible for us to activate a larger area to create more tissue flow. In a way, this serves our purpose better in creating local tissue flows to probe the response of individual EM cells.

Finally, we have managed to reproduce our *ex vivo* data *in vivo*, including a negative control as requested by the reviewer. We detailed our setup in the updated Figure 5I with a detailed description in the Materials and Methods section as follows:

"*In vivo optogenetics*
For in vivo optogenetics, we injected 20 pg of the Opto-IP mRNAs into all 4 animal blastomeres. For each embryo, we grafted a Ruby-LifeAct-labelled aVBI explant from a donor embryo to ensure no cross-contamination. Embryos were left to develop to around Stage 26 to 28, when EM cells have migrated out from the aVBI. We then laid the embryos onto their side and mounted them in 0.8% low melting agarose dissolved in 0.1X MMR mixed with Tricane (0.24 mg/ml) in a glass-bottom dish. A square glass coverslip was quickly placed on top and gently pressed down. To perform the optogenetic induction, a region next to the target EM cell was activated. A z-interval of 1 µm was used to ensure EM cells in the deeper tissue layer were captured. Imaging conditions are as follows: Zeiss880 confocal mode, X40 OIL lens, wavelength 454 nm, 10% laser power, consecutive interval, started at Frame 2, and repeated every frame until the end. Both the ectoderm and the EM cells were imaged with a 568 nm wavelength, 5% laser power, and a digital gain of 700."

We discussed this result in Figure 5J, and SuppFigure 4E and F, and added the following section to the main text:

"Finally, we asked whether the optogenetic response observed ex vivo was also conserved in vivo. We first expressed the Opto-IP system in the entire ectoderm layer of acceptor embryos before grafting aVBI tissue containing Ruby-LifeAct-labelled EM cells from donor embryos lacking the Opto-IP system. We left the embryos to heal and grow to around stage 25 to 27 to ensure that some EM cells have had the time to spread into the ectoderm before imaging

(Figure 5I). Upon photoactivation, the ectoderm visibly contracts at an average speed of 0.6 µm/min, which is also in line with the speed of a naturally moving ectoderm in wildtype embryos (Figure 5J, ia, ib, SuppFigure 4E, i, ii). This contraction biased a pre-existing protrusion and simultaneously induced the formation of a new protrusion toward the contracting ectoderm in the neighbouring EM cell (Figure 5J, ic, SuppVideo 26). Importantly, this response was not observed in the control condition, where the Opto-IP constructs were not expressed and therefore, no ectodermal contraction occurred upon blue light activation (SuppFigure 4F, SuppVideo 27). Measuring the area of protrusion extension and retraction of EM cells over time shows no change in both parameters in the control condition (Figure 5J, ii, dotted lines), while significant increases over time were observed in the photoactivated condition (Figure 5J, ii, solid lines). The angle of alignment α between EM cells' protrusion and the centre of the photoactivation region was similarly decreased from around 80° to around 30° (Figure 5J, iii, green line), while no change was observed in the control embryos (Figure 5J, iii, blue line)."

-In experiments of Fig. 6 how do we know the genetic modification does not change something secreted by the cells? Here it would be relevant to investigate gene expression changes or functionally test supernatants from these cells.

We believe that DD1 does not affect any relevant chemical signalling pathways because in our *ex vivo* grafting experiment shown in Figure 6F, EM cells behave the same between the control and the DD1-injected condition. If DD1 affected some secreted chemical, we would expect to see changes in their behaviour *ex vivo*. The fact that we only observed the effect *in vivo* suggests that it was likely due to the physical change in the embryo morphology instead.

Testing for conditioned media is not trivial in our system, as explained previously.

-The data presentation is generally good but some minor points here: In Fig. 5, some annotations inside images and scale bar are of low resolution and not clearly visible. In Fig. 6 membrane RFP si labelled in blue and can be confusing, perhaps a neutral white colour might be clearer. The videos are very helpful and well-presented, but it would be practical to consolidate some of them into fewer movies.

We thank the reviewers for raising these points. We have now tried to increase the size of some of the labels as best as we could, but since the figures are all quite densely packed to fit the flow of the story, it is not always possible to enlarge everything without compromising the aesthetics and the organisation as a whole.

For Figure 6, the RFP-membrane colour is purely for labelling purposes to distinguish between the acceptor and the donor explant and does not bear any significance in terms of interpreting the result of the experiment. We chose the colour cyan and magenta as this is one of the consensuses in the field for colour-blind readers. We believe that a neutral white colour overlaying a brightfield image would not be a good choice in this case.

In terms of the videos, we have thought about this as well. Due to the large amount of data in our paper, we found that splitting them up and labelling them individually helps readers find the relevant videos faster. Consolidating them into larger videos, in our experience, makes it more difficult to look for the right videos. However, if the reviewers and editor think that consolidating the videos is necessary, we would be happy to do so.

-The authors can discuss some additional points in the discussion section, such as alternative interpretations of the data. For example, is it possible that the fragmentation process could have effect on gene expression and differentiation of the EM cells and how could this be accounted for? And how could other tissue factors like the endoderm, further influence the migration pattern of EM cells?

We thank the reviewers for these interesting points. We have extensively discussed other alternative interpretations of the data, including potential chemotaxis, haptotaxis, and passive dragging effects in the discussion. We also added the following paragraphs:

"It is, however, worth noting that so far, our ex vivo and in vivo experiments have not specifically considered the co-movement of the endoderm or mesoderm. It is currently not possible to visualise beyond 2 or 3 cell layers deep in a Xenopus embryo, so analysing their correlation in vivo is not possible. Ex vivo culture might be a possible approach; however, accurately preserving the anatomical organisation of all 3 germ layers poses another challenge. Another question is whether passive dragging could explain collective-assisted directional migration. Our observations that the ectodermal flow induces an actin-rich protrusion in EM cells suggest that passive dragging is not dominant. Moreover, if passive dragging were a major contribution to EM cell dispersion, we would not expect to detect a 5-minute delay in EM cells' response. Nevertheless, teasing out the relative contributions of passive and active responses is an interesting question for future studies."

"We also noted that while we did not find a strong contribution of chemotaxis on EM cell dispersion ex vivo, we cannot rule out its contribution in vivo. Perhaps, a gradient of secreted or bound chemoattractants[65,107] is present in an intact embryo, or EM cells can create their own path through a self-generated gradient[108–110]. One of the interesting observations is the decrease in both the migration speed and directionality of EM cells when explants were treated with heparanase. Heparanase digests the sugar chain heparan sulfate, which is a typical post-translational modification for cell surface proteins[111,112]. Heparan sulfate has many different roles, including regulating protein functions[113], protein-protein interactions through binding to and patterning of morphogens[114,66], and cell-cell interactions such as during transendothelial migration[64,67]. EM cells seem to respond to the loss of heparan sulfate, which could be an indication of the existence of a haptotactic gradient of an unknown molecule bound to the surface of ectodermal cells. It is also possible that the loss of heparan sulfate on the surface of EM cells or ectodermal cells directly affects their cellular activities or cell-cell interactions through disrupting the functions of glycosylated transmembrane receptors such as syndecan[113]. Combined with the fact that EM cells' migration was impaired when the ectoderm was physically disrupted both ex vivo and in vivo, this result supports the hypothesis that direct physical interactions between EM cells and surrounding ectodermal cells are more relevant to their migration in our system."

The fragmentation could potentially affect gene expression, but this is unlikely to be relevant to our results. For example, in SuppVideo 3, EM cells in both intact and fragmented conditions left the central cluster almost at the same time. These fragmented clusters just mean that the area covered by the ectoderm is smaller, so EM cells will spend less time within the ectoderm, but this does not mean they spend less time in the initial mesoderm cluster. The fragmentation did not trigger premature migration, which would have been an indicator of changes in their development. Secondly, in the native condition, we also see the immediate shape changes when EM cells reach the edge of the explant and escape outside. These cells are more likely to be older than cells that just escaped from the mesoderm, yet they responded in the exact same manner regardless of their developmental stage, and this has been consistent across all our experiments. This indicates that the changes in the shape of EM cells with or without the ectoderm is more likely to be a general physical effect, regardless of gene expression or developmental stages. Therefore, we have no reason to believe that EM cells in our fragmented explants would behave any differently. The *Xenopus laevis* model is also known for its robustness in development, and this is why explant systems from these embryos have been used for decades to model and study development. So, we respectfully do not think this point raised by the reviewers is a major problem.

-In the discussion section, the authors relate their observations to the extravasation process, where cells flatten due to changes in integrins downstream of chemokine activation. Here again, they can consider discussing a link to chemoattractant patterning and the presentation/immobilisation of chemokines on tissue surfaces.

We have now added a paragraph in the discussion:

"On one hand, free EM cells behave somewhat similarly, where they remain round outside of the ectoderm, but will quickly assume a flattened shape once inside. This could potentially be explained by the chemical environment of the ectodermal cells, such as the presence of the glycocalyx coat and integrin receptors, much like those present on blood vessels[132], or by the physical compression from the ectodermal layer."

Reviewer #5 (Remarks to the Author):

I co-reviewed this manuscript with one of the reviewers who provided the listed reports. This is part of the Nature Cell Biology initiative to facilitate training in peer review and to provide appropriate recognition for Early Career Researchers who co-review manuscripts.

Reviewer #1:
Remarks to the Author:
Overall, we believe the authors have adequately addressed our concerns and those of the other reviewers. The theoretical model with the added source term now agrees quantitatively with the experimental data, which substantially improves the impact. For visual clarity the authors may want to consider using open markers for the data in the model-data comparison panels, as we found the combination a bit difficult to parse. The improved statistics and additional optogenetic experiments provide support to the authors claims. We appreciate the expanded discussion on the passive effects of friction vs active polarization and believe the cartoons are much improved. We now recommend publication.
We thank reviewers for their constructive and helpful criticism that has strengthened our manuscript. Figure 4L is now updated with open marker.

Reviewer #2:
Remarks to the Author:
I co-reviewed this manuscript with one of the reviewers who provided the listed reports. This is part of the Nature Cell Biology initiative to facilitate training in peer review and to provide appropriate recognition for Early Career Researchers who co-review manuscripts.

Reviewer #3:
Remarks to the Author:
I appreciate the authors' careful and thorough responses to these comments. The revised version addresses my major concerns in a clear and constructive manner.

In particular, I appreciate the significant effort the authors have made to add new experiments, test alternative approaches, and clarify points that were confusing to me in the original submission. These additions have strengthened the manuscript and made the interpretation of the results more balanced and better supported.

We thank the reviewer for their time and effort in strengthening our manuscript.

Reviewer #4:
Remarks to the Author:
The authors have performed a careful and comprehensive revision that has addressed my comments. The additional experiments and quantifications are helpful in interpreting the findings and the editorial changes have improved the clarity of the manuscript. I also appreciate the additional discussion on alternative interpretations of data.

I only have a few minor comments/suggestions:
-The PTX/anti-CSF treatments had small effects but do not appear insignificant. It is hard to exclude incomplete inhibition in such experiments, so the authors could articulate the limitations of these perturbations.
We want to thank the reviewers for their time and effort that has helped us strengthen our manuscript.
We have now added a small caveat to this section (due to the strict word limitation):

"*While it is possible that there might be incomplete inhibition, the observed effects are small enough to suggest that chemotaxis is likely not a major player controlling the radial dispersion of EM cells ex vivo*."

- The experiments with heparinase revealed a marked influence. The authors can discuss these findings also in relation to haptotactic chemokine gradients that have been shown to be sensitive to heparinase treatment (10.1126/science.1228456; 10.1016/j.cub.2012.11.018).

We have incorporated these references to the discussion:

"*EM cells seem to respond to the loss of heparan sulfate, which could be an indication of the existence of a haptotactic gradient of an unknown molecule bound to the surface of ectodermal cells*[112,113]."

Reviewer #5:

Remarks to the Author:

I co-reviewed this manuscript with one of the reviewers who provided the listed reports. This is part of the Nature Cell Biology initiative to facilitate training in peer review and to provide appropriate recognition for Early Career Researchers who co-review manuscripts.
